# Design and synthesis of constrained bicyclic molecules as candidate inhibitors of influenza A neuraminidase

**DOI:** 10.1371/journal.pone.0193623

**Published:** 2018-02-28

**Authors:** Cinzia Colombo, Črtomir Podlipnik, Leonardo Lo Presti, Masahiro Niikura, Andrew J. Bennet, Anna Bernardi

**Affiliations:** 1 Università degli Studi di Milano, Dipartimento di Chimica, Milano, Italy; 2 Department of Chemistry, Simon Fraser University, Burnaby, British Columbia, Canada; 3 University of Ljubljana, Faculty of Chemistry and Chemical Technology, Ljubljana, Slovenia; 4 Faculty of Health Sciences, Simon Fraser University, Burnaby, British Columbia, Canada; National Cancer Institute at Frederick, UNITED STATES

## Abstract

The rise of drug-resistant influenza A virus strains motivates the development of new antiviral drugs, with different structural motifs and substitution. Recently, we explored the use of a bicyclic (bicyclo[3.1.0]hexane) analogue of sialic acid that was designed to mimic the conformation adopted during enzymatic cleavage within the neuraminidase (NA; sialidase) active site. Given that our first series of compounds were at least four orders of magnitude less active than available drugs, we hypothesized that the new carbon skeleton did not elicit the same interactions as the cyclohexene frameworks used previously. Herein, we tried to address this critical point with the aid of molecular modeling and we proposed new structures with different functionalization, such as the introduction of free ammonium and guanidinium groups and ether side chains other than the 3-pentyl side chain, the characteristic side chain in Oseltamivir. A highly simplified synthetic route was developed, starting from the cyclopropanation of cyclopentenone and followed by an aziridination and further functionalization of the five-member ring. This allowed the efficient preparation of a small library of new bicyclic ligands that were characterized by enzyme inhibition assays against influenza A neuraminidases N1, its H274Y mutant, and N2. The results show that none of the new structural variants synthesized, including those containing guanidinium groups rather than free ammonium ions, displayed activity against influenza A neuraminidases at concentrations less than 2 mM. We conclude that the choice and positioning of functional groups on the bicyclo[3.1.0]hexyl system still need to be properly tuned for producing complementary interactions within the catalytic site.

## Introduction

**Fig 1:**
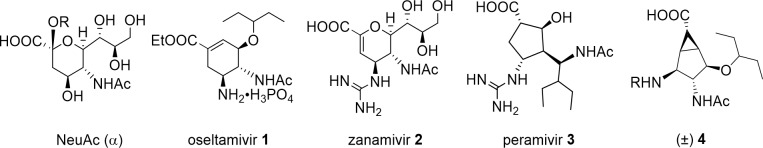
Sialic acid (α configuration), oseltamivir 1, zanamivir 2, peramivir 3 and the bicyclo[3.1.0]hexane scaffold 4.

**Fig 2:**
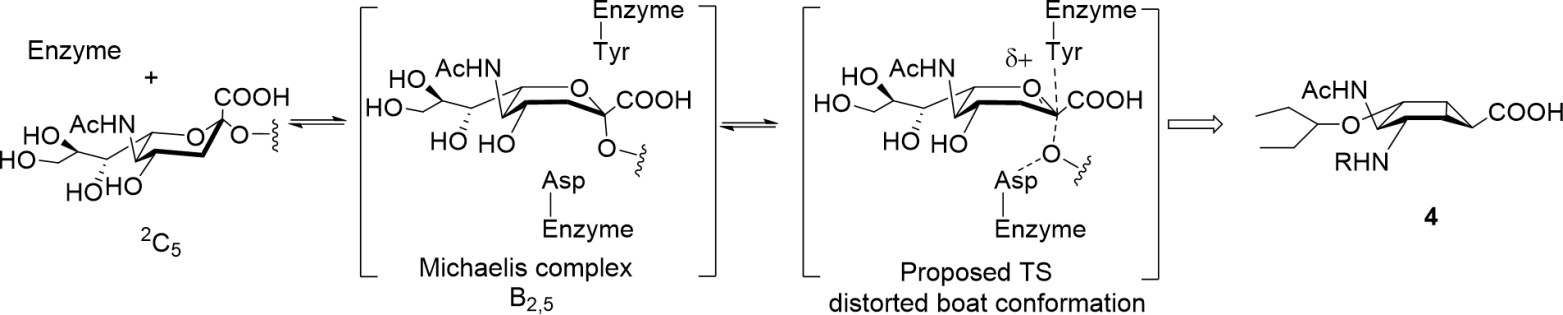
Sialic acid ring distortion during catalysis and mimic 4 in its predicted conformation.

Influenza A viruses are the most virulent human pathogens among the three influenza types A, B, C. The virus uses its neuraminidases (sialidases, NA), expressed on the surface of viral envelope, for mobility through the mucus in the respiratory tract and for spreading the infection.[1–2] After invasion and replication through the host cell machinery, the budded virions are anchored to sialic acid (NeuAc) residues on the host cell membrane via interaction with viral hemagglutinin (HA). The viral NA, at this point, cleaves the sialic acid residues from the anchored glycoconjugates and releases new virus particles. Antiviral drugs, like oseltamivir **1**,[3] zanamivir **2**,[4] and peramivir **3**[5] (Fig 1) have been developed based on an understanding of the neuraminidase mechanism of action, by mimicking sialic acid undergoing cleavage in the binding site. For retaining sialidases, the glycosylated enzyme intermediate generated in the catalytic pocket is subjected to both glycosylation and deglycosylation via transition states (TS) that have an oxacarbenium ion character and feature a distorted six-membered ring (Fig 2).[6–9] Oseltamivir (**1**, Fig 1) uses a cyclohexene ring in place of the sugar pyran to mimic this distortion. The ring is substituted at both C4 and C6 with an amino group, replacing NeuAc hydroxyl groups, and at C-5 with a 3- pentyl ether chain in place of NeuAc glycerol side chain. Zanamivir (**2**, Fig 1) conserves both the NeuAc pyran ring and glycerol side chain at C6, but is modified at C4, where the hydroxyl group is replaced with a guanidino group. Peramivir (**3**, Fig 1), built on a cyclopentane skeleton, maintains the guanidino group and other key elements essential for NA binding.

Molecular modeling studies suggest that the Michaelis complex between influenza NA and its substrate forces the pyran ring into a B_2,5_ or a ^4^S_2_ conformation (Fig 2, B_2,5_ boat shown),[10] whereas a ^6^S_2_ skew-boat conformation has been proposed for bacterial sialidases.[8] The introduction of a double bond into the six-membered ring of **1** and **2** has been used as a general strategy to mimic the flattened geometry of the enzymatic TS. Recently, we synthesized bicyclo[3.1.0]hexane analogues **4** (Fig 1)[11] based on the hypothesis that these molecules should also provide the ring distortion required to mimic the TS structure (Fig 2).

The synthetic approach to access these derivatives involved a photochemical pyridine ring contraction followed by a Johnson-Corey-Chaykovsky cyclopropanation, allowing systematic variation of the relative stereochemistry of the scaffold’s stereocenters.[11] The compounds displayed 'slow-binding' time-dependent inhibition of N1 and N2 sialidases with IC_50_ values in the micromolar range, i.e. four orders of magnitude less efficient than **1–3**. The strongest inhibition exhibited by these compounds (IC_50_ 10 μM) was observed with compound **4a** (R = 4-phenylbenzyl, Fig 1).[11] Despite the low activity observed, the results provided proof of principle for the potential of the bicyclo[3.1.0]hexane scaffold in NA inhibition and offered some insight into the requirements for the configuration and substitution patterns of constrained cyclopropyl sialic acid analogues.

**Fig 3:**
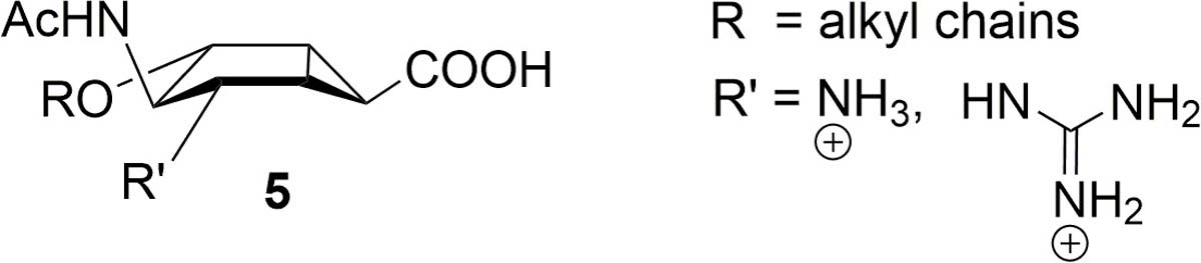
Compounds of general formula 5, described in this paper.

To further develop the structure activity relationship of this new scaffold, we synthesized a second set of compounds in order to analyze the effect of replacing the amino group of **4** with a larger guanidium moiety and probe the role of the ether side chain (Fig 3, compounds of general formula **5**). Indeed, the introduction of a guanidium group as a substituent of the dihydropyran ring was a critical feature in the design of zanamivir.[4] The guanidino unit in zanamivir and peramivir engages in hydrogen-bonding interactions that do not occur in the oseltamivir-neuraminidase complex, as revealed by available cocrystal structures.[4]^,^[5] In particular, the guanidino group in zanamivir and peramivir is involved in charge-based interactions and salt bridges with residues Glu 119 and Glu 227 (N2 numbering), through the replacement of a water molecule. These additional interactions allowed speculation about the lack of resistance of some oseltamivir resistant strains toward inhibitors containing the guanidino function [12] and led to the synthesis and evaluation of NA inhibitors with mixed features from oseltamivir and zanamivir.[13]^,^[14]^,^[15]

It is now well known that the 3-pentyl ether side chain of oseltamivir is a major cause for resistance: neuraminidases must undergo a significant rearrangement to form a pocket for oseltamivir binding and this movement can be affected by mutations, such as H274Y.[16] Resistance to oseltamivir is a growing concern,[17] also considering that permissive secondary mutations have enabled the evolution of resistant strains.[18] The 3-pentyl ether moiety is a key element in the design of oseltamivir, but there is no reason to assume that it would be optimal on the bicyclo[3.1.0]hexane framework. Preliminary modeling data on the complex between N1 and compound **4** (R = H, Fig 1) had suggested that simultaneous binding of isopentyl group and amine cannot be achieved from this scaffold.

In the current study, to carry out the synthesis of new derivatives **5**, a new, much simplified synthetic route for the bicyclo[3.1.0]hexane framework was adopted, starting from cyclopropanation of cyclopentenone followed by aziridination and further functionalization of the five-membered ring. This strategy allowed the efficient preparation of a small library of 10 bicyclic ligands (**5**, Fig 3), densely functionalized. These compounds were tested against various influenza A neuraminidases.

## Results and discussion

**Fig 4:**
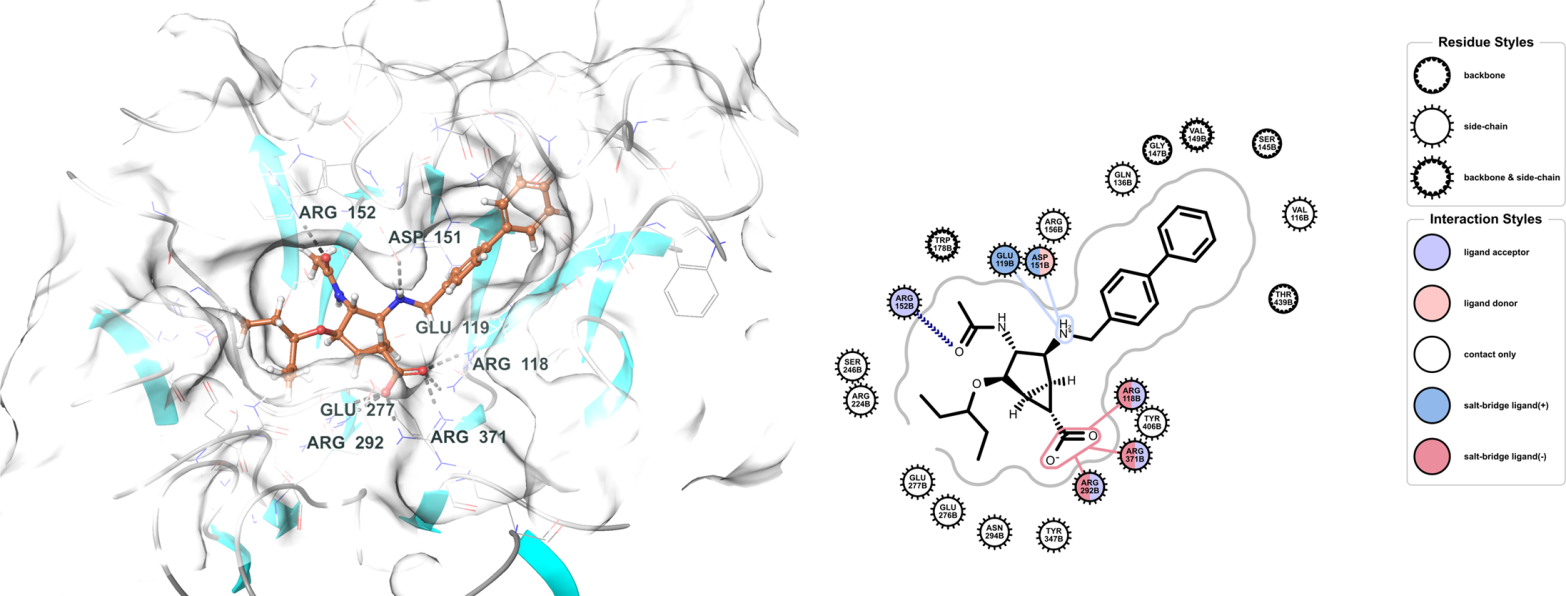
Induced fit docking of ligand 4a (R = 4-phenylbenzyl) with N1 neuraminidase (source: pdb 2HU0).

The results obtained in our previous campaign highlighted **4a** (R = 4-phenylbenzyl, Fig 1) as the most active stereoisomer of the series.[11] Docking using Schrödinger’s Induced Fit Docking (IFD) protocol[19] suggested that compound **4a** could make all the relevant contacts in the carboxylate binding pocket of N1 and that its phenyl-benzyl moiety extended into the 150 cavity, contributing non-polar interactions to the stability of the complex (Fig 4). On the other hand, the distance between the charged amino moiety of **4a** and the side chain of Glu119 is too large (about 5 Å) to establish a solid salt bridge.

**Fig 5:**
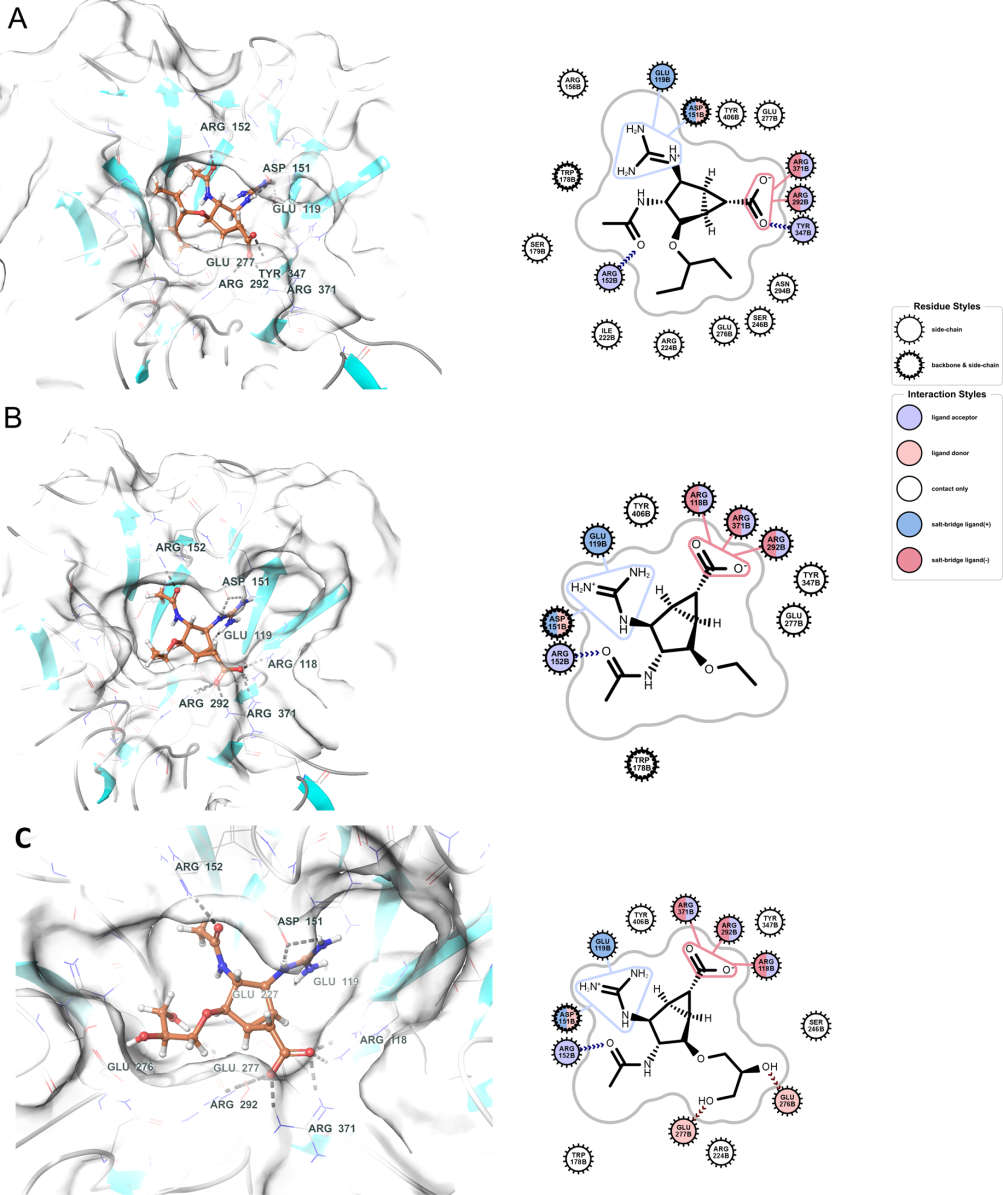
Docking of ligand 5 with various substitutions in N1 neuraminidases (sources: pdb 2HU0 and 2HU4). (A) Docking of **5A** ${R’=NHC({NH}_{2})}_{2}^{+}$ R = CH(CH_2_CH_3_)_2_); (B) Docking of **5B (**${R’=NHC({NH}_{2})}_{2}^{+}$ and R = CH_2_CH_3_); (C) Docking of **5C** ${R’=NHC({NH}_{2})}_{2}^{+}$ and R = CH_2_CH(OH)(CH_2_OH).

As observed during the development of zanamivir,*[4]* some of these issues could be solved by increasing the size and surface of the basic group from an ammonium ion to a guanidinium group. According to IFD calculations, introduction of a guanidinium group in the scaffold (compounds **5**, ${R’=NHC({NH}_{2})}_{2}^{+}$) is expected to provide better orientation of the ligand within the binding site. As can be seen from Fig 5, the guanidino group should be optimally exposed toward the side chains of Glu119 and Asp151 forming salt bridge interactions. We also observe that 3-pentyl moiety of **5A** (${R’=NHC({NH}_{2})}_{2}^{+}$ R = CH(CH_2_CH_3_)_2_) fits nicely into the pocket surrounded by Ser246, Arg224, Glu277, Glu278 and Asn294 (Fig 5A). On the other hand, the substitution of the 3-pentyl moiety with an ethyl group (Fig 5B, compound **5B** with ${R’=NHC({NH}_{2})}_{2}^{+}$ and R = CH_2_CH_3_) does not give maximal stabilization for hydrophobic interactions within the pocket mentioned above. The introduction of a dihydroxypropoxy ether moiety, similar to the glycerol side chain of NeuAc and zanamivir, is expected to produce beneficial interactions within the binding pocket of N1 (Fig 5C, compound **5C** with ${R’=NHC({NH}_{2})}_{2}^{+}$ and R = CH_2_CH(OH)(CH_2_OH)). Compound **5C** is interesting because it contains all of zanamivir’s pharmacophoric groups attached to the bicyclic scaffold. Indeed, the interaction diagrams of compound **5C** (${R’=NHC({NH}_{2})}_{2}^{+}$ and R = CH_2_CH(OH)(CH_2_OH)) and zanamivir are very similar. The major difference is in the contacts of the guanidino moiety which in the case of zanamivir is calculated to engage in an additional contact with Glu227.

**Fig 6:**
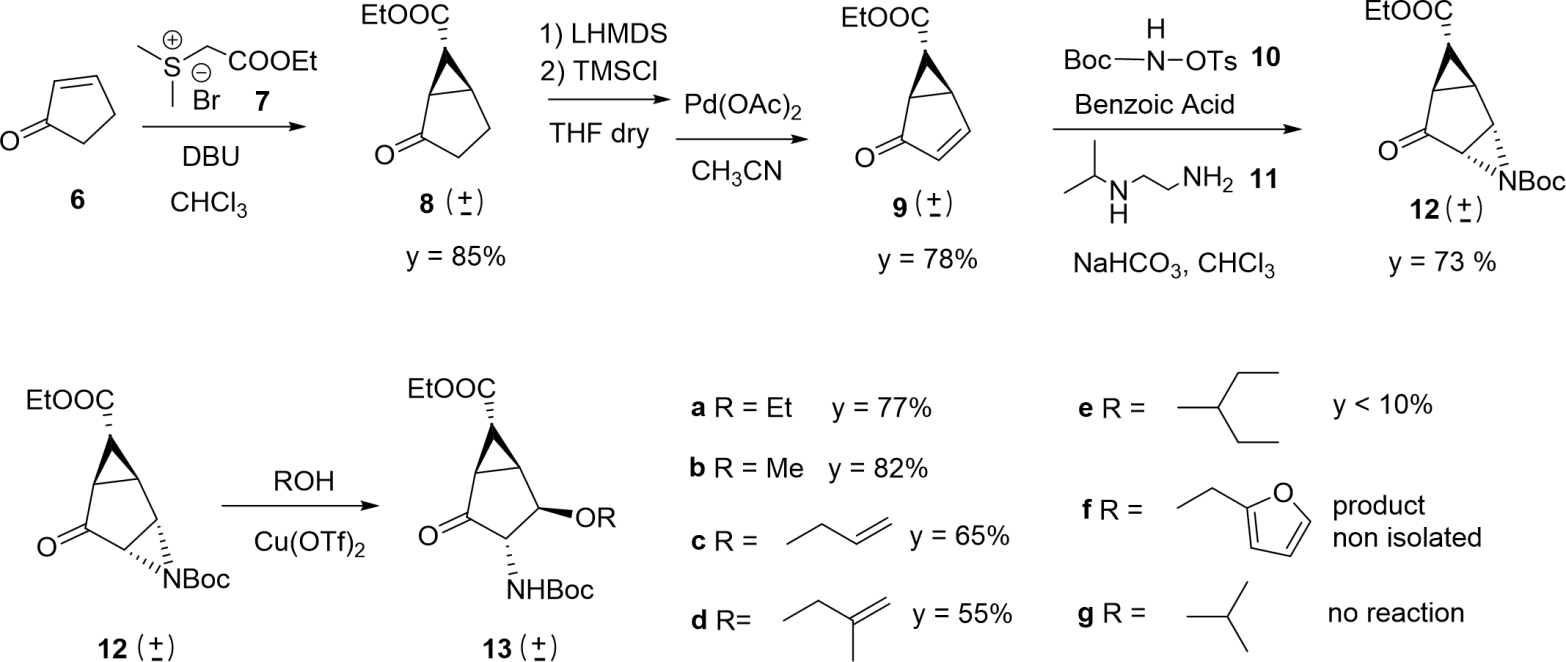
Synthesis of the key aziridine intermediate 12 and aziridine opening reaction.

With these docking hypotheses in hand, we focused our efforts on developing a direct protocol for the synthesis of new derivatives **5**, with the aim of comparing the effect of different substitutions of the bicyclo[3.1.0]hexane framework on NA inhibitory activity. The synthetic strategy was based on the cyclopropanation of cyclopentenone **6** with ethyl-(dimethylsulfuranylidene)-acetate (EDSA), generated in situ from the corresponding sulfonium bromide **7** and DBU in CHCI_3_ at r.t. (Fig 6).[20]^,^[21] The cyclopropane **8** was obtained as a racemic mixture in 85% yield. The second double bond was introduced in the five-membered ring in 78% yield by transforming the ketone in the corresponding silylenolether (LHMDS, TMSCl), which was then oxidized to the enone **9** with Pd(OAc)_2_.[22] The aziridination of **9** was inspired by asymmetric catalytic aziridination protocols[23]^,^[24] that make use of *N*-Boc-sulfonylamide **10**[25] and chiral bisamines, but was actually performed with achiral bis-amino ligand **11**, to afford **12** in 73% yield. Compared to our previous route,[11] this approach afforded the key aziridine intermediate in high yields and in only three synthetic steps, avoiding the use of photochemical reactions that were difficult to scale-up to the required levels.

Opening of the aziridine catalyzed by Copper (II) salts[26] with a variety of alcohols allowed us to introduce variability at the ether function in the final molecules (Fig 6). Primary alcohols reacted in good yields giving one major diastereoisomer (**13a-d**) in good to acceptable yields. Reaction with furfuryl alcohol generated many impurities, mainly due to cross-reactivity of the furan ring, and product **13e** was not isolated. Isopropanol did not react with **12**, even after prolonged heating and addition of more copper triflate. The reaction with 3-pentanol produced a complex mixture of products: opening of the aziridine took place from both faces of the ring and at both positions 3 and 4 (Figure A in S3 File). Elimination of 3-pentyl ether from the primary aziridine opening product was also observed, as described previously for this type of molecules.[27] Different Lewis acids (BF_3_∙Et_2_O, Sn(OTf)_2_, Zn(OTf)_2_, Ti(OEt)_4_, MgCl_2_) and reaction conditions (increasing temperature and concentration) did not improve the desired reactivity. Attempts to convert the *N*-Boc aziridine **12** to *N*-acetyl aziridine, in order to reduce the steric hindrance of the substrate, failed. Despite all efforts, the reaction with 3-pentanol never afforded yields above 10% and was not scalable to an adequate level to complete the synthesis of final compounds bearing the 3-pentyl ether chain.

**Fig 7:**
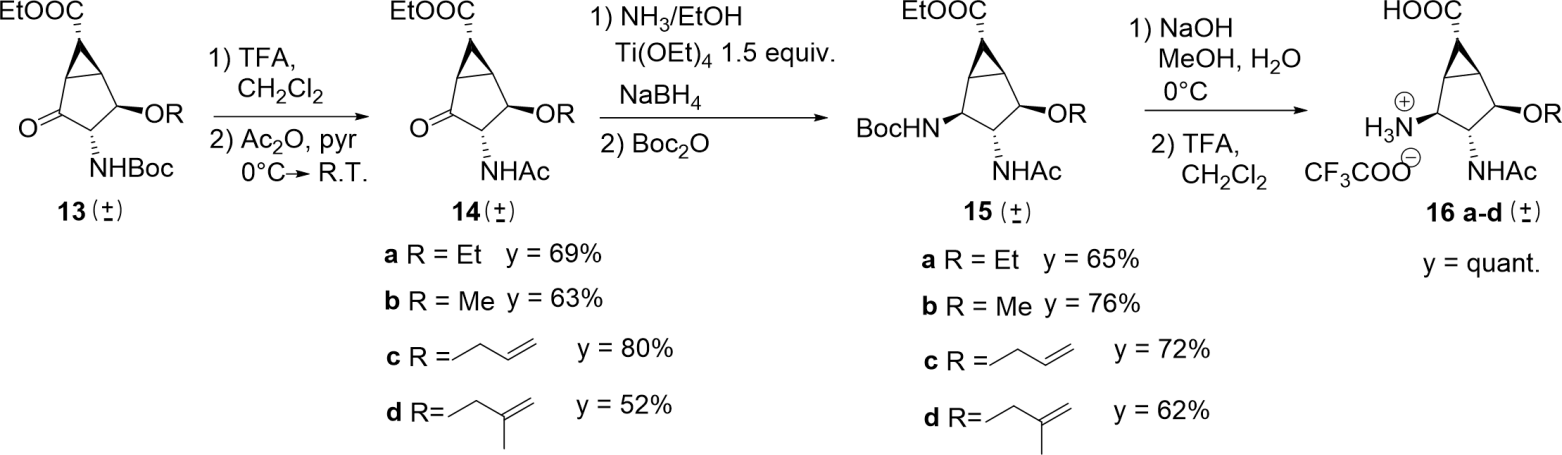
Synthesis of amino acids 16a-d.

Compounds **13a-d** were then subjected to removal of *N*-Boc protection and nitrogen acetylation to give **14a-d** (Fig 7). The ketone in position 2 was converted to the corresponding amines through reductive amination (ammonia, Ti(OEt)_4_, NaBH_4_)[28] followed by *N*-Boc protection, to afford the differentially protected bis-ammino esters **15a-d**. Removal of the protecting groups from **15a-d** (NaOH, then TFA; Fig 7) afforded amino acids **16a-d** in quantitative yields.

**Fig 8:**
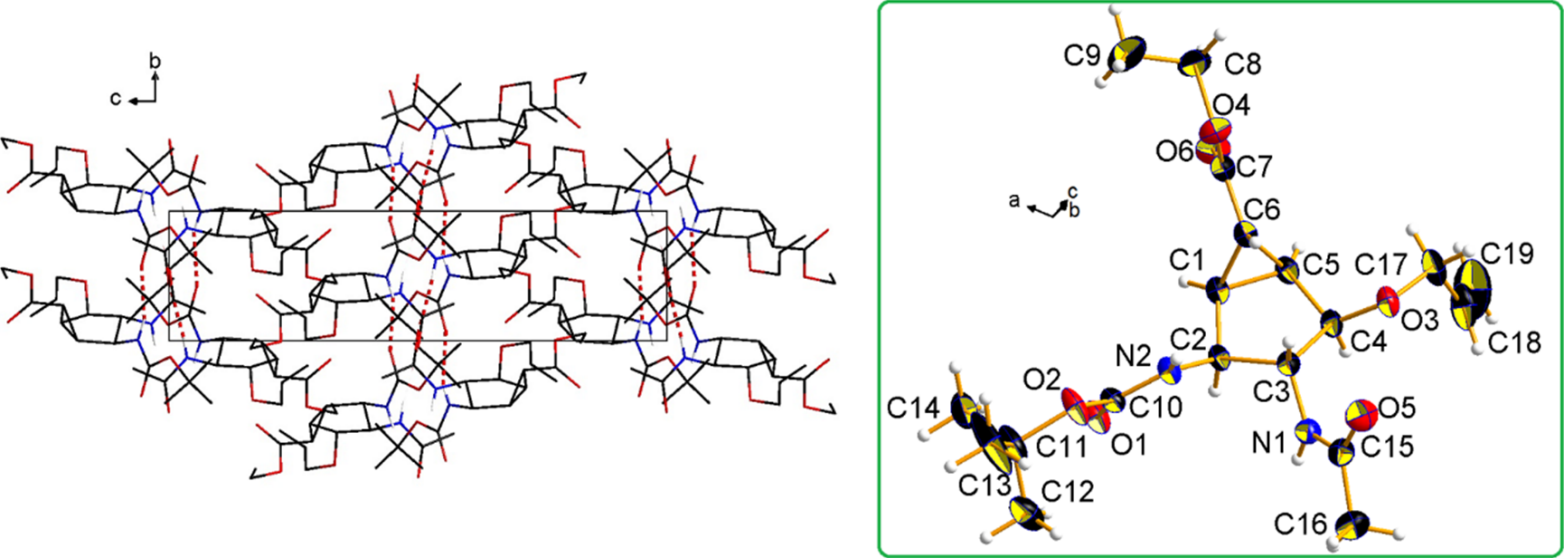
X-ray structure of 15c at room temperature, crystallized from acetone (4°C). Right: crystal packing viewed along *a* is shown, with hydrogen atoms not involved in hydrogen bonds (red dashed lines) omitted for clarity. Left: asymmetric unit of **15c**, with the atom numbering scheme. Thermal ellipsoids are drawn at the 30% probability level.

Compound **15c** was crystallized (acetone, 4°C) allowing the determination of the relative configuration of the products (Fig 8) and the final targets. The compound is chiral and crystallizes in the centric space group P21/c as a racemate, with one molecule per asymmetric unit (for details see Sup. Info). Fig 8 shows the absolute configuration of the stereocentres for the arbitrary choice of one enantiomer. The configurational descriptors of the stereogenic centres are either C1 (R), C2 (S), C3 (R), C4 (R), C5 (S) and C6 (S) or C1 (S), C2 (R), C3 (S), C4 (S), C5 (R) and C6 (R) (see Fig 8 for the atom numbering).

Two significant hydrogen bond (HB) donors are present, namely the amide N1–H1 and N2–H2 groups (Fig 8). These functions are employed to set up supposedly strong H-bonded pillars along the monoclinic *b* axis, which involve the carbamate and the acetamide carbonyl groups (Fig 8, Figure A and B in S1 File, Table A in S1 File). Cumbersome substituents, on the other hand, are allocated in the free space between adjacent pillars and extend mainly in the (*a*,*c*) plane (Fig 8).

**Fig 9:**
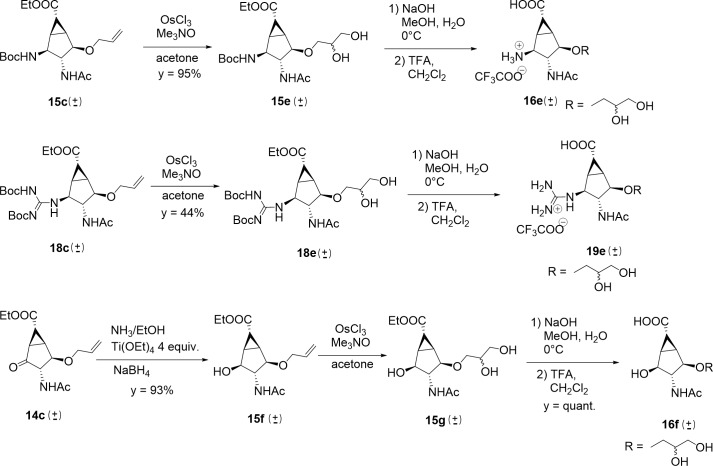
Synthesis of guanidines 19a-d.

Removal of the t-butylcarbamate from **15a-d** (TFA; Fig 9) also set the stage for installation of the guanidino group on the bicyclic framework. To this end, the resulting amines were treated with *N*,*N'*-bis(tert-butoxycarbonyl)thiourea **17** and Mukayiama’s reagent, following Lipton’s guanylation protocol,[29] to afford the corresponding *N*,*N'*-bis(tert-butoxycarbonyl)guanidines **18a-d** in satisfactory yields. Removal of the protecting groups (NaOH, then TFA; Fig 9) afforded the target guanidinium derivatives **19a-d**.

**Fig 10:**
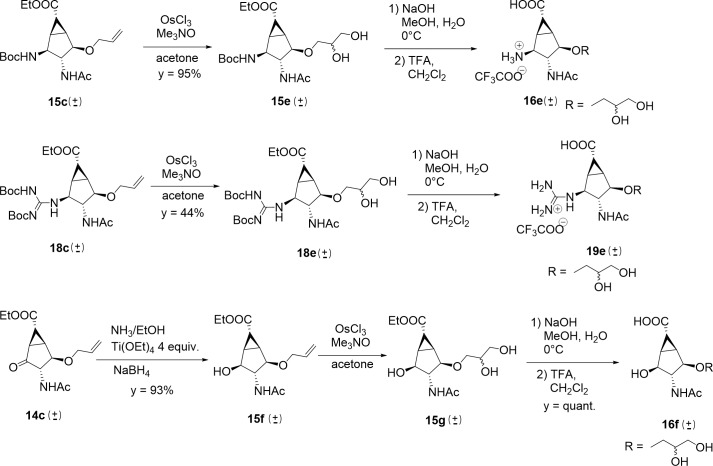
Synthesis of the dihydroxypropoxy side chain derivatives 16e, 19e and 16f.

To introduce the dihydroxypropoxy ether side-chain modification, the allyl ether in **15c** and **18c** was dihydroxylated (OsCl_3_, Me_3_NO, acetone/water) to afford glycerol **15e** and **18e**, as a diastereomeric mixture at the newly generated stereogenic center (Fig 10). The use of Sharpless methodology with AD-mix α and β did not result in better stereoselectivity, as revealed by ^13^C-NMR of final compounds obtained with the three different methods. A derivative with a hydroxy group in position 2 was also prepared (Fig 10, compound **16f**), to systematically compare the effect of ammonium and guanidinium substitution on the compounds activities. By adding 4 equivalents of Ti(OEt)_4_ in the reduction of the ketone with ammonia in EtOH, alcohol **15f** formed preferentially (y = 93%), as revealed by NMR analysis (Supp. Info) and was then subjected to dihydroxylation to afford compound **15g**. Protecting group removal afforded final compounds **16e**, **19e** and **16f** (Fig 10).

### Enzyme inhibition and cell culture assays

**Fig 11:**
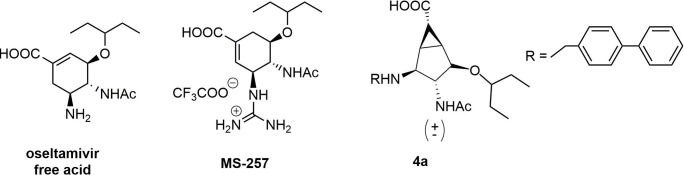
Compounds used for control experiments for enzyme inhibition.

Initially, we measured *K*_m_ values of the three NAs (A/Anhui/1/2005 H5N1 wild-type and H274Y mutant, and A/Chicken/HongKong/G9/1997 H9N2) for the MUNANA substrate in MES buffer (32.5 mM 2-(*N*-morpholino)ethanesulfonic acid, 4 mM CaCl2, pH 6.5). The *K*_m_ values were 12.8 ± 1.9 μM for N1 (H5N1), 26.5 ± 1.7 μM for N1 H274Y mutant, and 70 ± 6.8 μM for N2 (H9N2). The enzymatic activity of the N1 H274Y mutant appears to be comparable to that of wild type viruses with compromised ability to bind oseltamivir.[16]-[30] Indeed, before starting the evaluation of new compounds, we performed control experiments with oseltamivir free acid, MS-257 (Fig 11, a known nanomolar inhibitor of influenza A neuraminidase)[13] and compound **4a** (compound belonging to the first series of compounds and previously tested).[11] The results are listed in Table 1. The inhibition constant (*K*_i_) of oseltamivir free acid for the mutant N1 H274Y showed an increase of 580-fold, compared to the wild-type. The *K*_i_ of MS-257 showed an increase of 95-fold. This is consistent with previously reported data, which showed MS-257 to be more active against an Oseltamivir-resistant virus H1N1 with the H274Y mutation compared to oseltamivir free acid.[31] MS-257 was also recently shown to be less susceptible to drug resistance toward a mutant group 2 influenza Virus NA.[14] Compound **4a** which was already 4 orders of magnitude less active than oseltamivir free acid against both N1 and N2, was not active toward the H274Y mutant in the range of concentrations tested (up to 1 mM). Compound **4a** and all new compounds reported in this manuscript are racemates; as a result, we expect that the IC50 value could be lower than the one measured, if only one of the two enantiomers is active. All new compounds (**16 a-f** and **19 a-e**), were tested with N2, N1 and N1-H274Y and found to be inactive at 2 mM concentration. Inhibition experiments were not carried out at compound concentrations above 2 mM.

**Table 1:** Control experiments with known NAIs. *K*_i_ was determined for N1 wild-type andH274Y (both from virus A/Anhui/1/2005 H5N1) and N2 (A/Chicken/HongKong/G9/1997 H9N2).

Compound	Ki		
	N1	N1-H274Y	N2
**oseltamivir free acid**	0.15 ± 0.08 nM	86.6 ± 7.5 nM	0.67 ± 0.10 nM
**MS-257[13]**	0.17 ± 0.02 nM	16.2 ± 1.9 nM	0.21 ± 0.06 nM
**4a**	9.1 ± 1.3 μM	n.d.	13.2 ± 4.0 μM

In order to investigate the efficacy of the compounds against influenza A virus replication, the compounds were also tested in an in vitro replication inhibition assay using A/Puerto Rico/8/34 (PR8, H1N1) and A/Hong Kong/1/68 (HK1, H3N2) strains. Compound **MS-257** (Fig 11), previously tested in cell culture assays,[31] was used as a positive control. None of the new compounds showed replication inhibition efficacy.

## Conclusion

In conclusion, we presented herein the successful synthesis of new derivatives of a bicyclo[3.1.0]hexane scaffold containing six stereocenters and a variety of functional groups, designed with the aid of molecular modelling to engage in productive interactions within the NA binding site. A simplified synthetic route was developed, starting from the cyclopropanation of cyclopentenone and followed by an aziridination to achieve the common precursor that was then functionalized with various ether side chains other than the 3-pentyl side chain, the characteristic side chain in Oseltamivir. Some of these chains were installed as negative controls, like the methylether and the ethylether, while the allyloxy, the 2-methylallyloxy and the dihydroxypropoxy side chains were expected by induced fit docking calculations to positively engage in interactions in the NA binding site. However, none of the new structural variants that we could synthesize, including those containing guanidinium groups rather than free ammonium ions displayed activity against influenza A neuraminidases at concentrations less than 2 mM. Unfortunately, we could not install the 3-pentyl side chain in this new set of compounds. Overall, the information collected, together with the previously and here observed confirmed activity of compound **4a,** that features a 3-pentyl side chain moiety allowed us to speculate that this side chain could be of crucial importance for productive interactions of the bicyclo[3.1.0]hexane scaffold with influenza A neuraminidases. However, it appears that overall, docking calculations are overestimating the quality of the interactions generated by the bicyclo[3.1.0]hexyl systems within the NA binding site. This may also depend on the rather extreme flattening of the bicyclic ring system, best appreciated in the X-ray structure of **15c**, which distorts it away from the TS-like structure needed for optimal binding. We conclude that the choice and positioning of functional groups on the bicyclo[3.1.0]hexyl system still need to be properly tuned in order to elicit complementary interactions within the catalytic site.

## Materials and methods

General information: all chemicals were of analytical grade or better and were purchased from Sigma-Aldrich unless noted otherwise. ^1^H- and ^13^C-NMR spectra were acquired on a Bruker instrument and recorded at 600 or 400 MHz and 150 or 100 MHz, respectively. Spectra are reported as follows: chemical shift (δ ppm), multiplicity (s = singlet, d = doublet, t = triplet, q = quartet, quint = quintet, m = multiplet, bs = broad singlet), coupling constants (Hz). All assignments were confirmed with the aid of two-dimensional ^1^H-^1^H (COSY), ^1^H-^13^C (HSQC) and/or ^1^H-^13^C (HMBC) experiments using standard pulse programs. Processing of the spectra was performed using MestReNova software. Products numbering for spectral assignment is clarified in the Appendix A in S3 File. Analytical thin-layer chromatography (TLC) was performed on aluminum plates pre-coated with silica gel 60F-254. The developed plates were air dried, exposed to UV light, and/or sprayed with a solution containing molybdic reagents or permanganate reagents, and heated. Column chromatography was performed with an automated flash chromatography system. High resolution mass spectra were obtained by the electrospray ionization method, using a TOF LC/MS high-resolution magnetic sector mass spectrometer.

### Synthesis

Compounds **8** and **9** were prepared accordingly to Reference 21 and 22, respectively. Compound **10** was prepared accordingly to Reference 25. The analytical data were consistent with those reported in the literature.

Compound **12.** To a stirred solution of benzoic acid (730 mg, 6 mmol), sodium bicarbonate (2.545 g, 30 mmol), *N*-Boc-sulfonylamide **10** (3.272 g, 12 mmol) in CHCl_3_ (60 mL), *N*-isopropylethylendiamine was added (375 μL, 3 mmol) at room temperature, under nitrogen atmosphere. Compound **9** (1.0 g, 6 mmol) was added in one portion and the resulting mixture was stirred at room temperature overnight. Water was added (60 mL) and the aqueous solution was extracted with CHCl_3_ (3 x 30 mL) and the combined organic layers were washed once with saturated NaCl, dried over NaSO_4_ and evaporated. The resulting oily residue was purified by column chromatography (Hex:EtOAc; gradient: 5–10% EtOAc in 2 CV, 10–35% EtOAc in 8 CV) to afford compound **12** as a white solid (1.234 g, 73%). ^1^H NMR (400 MHz, CDCl_3_) δ 4.15 (q, *J* = 7.1 Hz, 2H, CH_2Et_), 3.45 (t, *J* = 2.5 Hz, 1H, H_4_), 3.00 (ddd, *J* = 4.8, 3.4, 1.2 Hz, 1H, H_1_), 2.91–2.85 (m, 1H, H_3_), 2.11–2.01 (m, 2H, H_5_, H_6_), 1.45 (s, 9H, CH_3Boc_), 1.25 (t, *J* = 7.1 Hz, 3H, CH_3Et_).^13^C NMR (101 MHz, CDCl_3_) δ 199.4 (C_2_), 168.4 (CO_OEt_), 157.6 (CO_Boc_), 83.1 (C_Boc_), 61.8 (CH_2Et_), 42.9 (C_4_), 41.2 (C_3_), 30.7 (C_6_), 29.4 (C_1_), 29.4 (C_5_), 27.9 (CH_3Boc_), 14.2 (CH_3Et_). ^1^H NMR (400 MHz, Acetone-*d*_6_) δ 4.14 (q, *J* = 7.1 Hz, 2H, CH_2Et_), 3.58 (t, *J* = 2.6 Hz, 1H, H_4_), 2.92 (ddd, *J* = 4.9, 3.3, 1.1 Hz, 1H, H_1_), 2.89 (dt, *J* = 2.6, 0.9 Hz, 1H, H_3_), 2.38–2.31 (t, *J* = 3.3, 1H, H_6_), 1.97–1.92 (m, 1H, H_5_), 1.45 (s, 9H, CH_3Boc_), 1.23 (t, *J* = 7.1 Hz, 3H, CH_3Et_). HRMS (ESI) m/z calculated for [C14H19NNaO5]^+^ 304.11554; found 304.11603.

#### General procedure for aziridine opening with alcohols

To a stirred solution of compound 12 (250 mg, 0.89 mmol) dissolved in alcohol (6 mL) under nitrogen atmosphere, copper triflate (160 mg, 0.44 mmol) was added and the reaction mixture was stirred at room temperature for 8 h (TLC Hex:EtOAc 7:3). The mixture was evaporated and residue purified by column chromatography (Hex:EtOAc; gradient: 5–35% EtOAc in 10 CV).

Compound **13a.** y = 77%. ^1^H NMR (400 MHz, CDCl_3_) δ 5.29 (d, *J* = 8.2 Hz, 1H, NH_Boc_), 4.28–4.16 (m, 1H, H_4_), 4.06 (m, 2H, CH_2Et_), 3.67–3.44 (m, 3H, H_3_, H_7_), 2.67–2.63 (m, 1H, H_5_), 2.45–2.29 (m, 2H, H_6_, H_1_), 1.33 (s 9H, CH_3Boc_), 1.26–1.06 (m, 6H, CH_3_(8), CH_3Et_). ^13^C NMR (101 MHz, CDCl_3_) δ 203.1 (C_2_)169.8 (CO_OEt_), 155.4 (CO_Boc_), 80.1 (C_Boc_), 77.9 (C_4_), 64.8 (CH_2Et_), 61.5 (C_7_), 58.6 (C_3_), 35.4 (C_1_), 30.2 (C_5_), 28.2 (CH_3Boc_), 26.4 (C_6_), 15.3 (CH_3Et_), 14.0 (C_8_). MS (ESI) m/z calculated for [C16H25NNaO6]^+^ 350.16; found 350.35.

Compound **13b.** y = 82%. ^1^H NMR (400 MHz, CDCl_3_) δ 4.99 (bs, 1H, NH_Boc_), 4.23–4.03 (m, 3H, CH_2Et_, H_4_), 3.74–3.52 (m, 1H, H_3_), 3.45 (s, 3H, CH_3-7_), 2.75 (q, *J* = 5.3 Hz, 1H, H_5_), 2.53–2.34 (m, 2H, H_1_, H_6_), 1.41 (s, 9H, CH_3Boc_), 1.26 (t, *J* = 7.1 Hz, 3H, CH_3Et_). ^13^C NMR (101 MHz, CDCl_3_) δ 203.1 (C_2_), 169.8 (CO_OEt_), 155.5 (CO_Boc_), 80.7 (C_Boc_), 80.0 (C_4_), 61.8 (CH_2Et_), 58.7 (C_3_), 57.2 (C_7_), 35.6 (C_1_), 30.0 (C_5_), 28.3 (CH_3Boc_), 24.6 (C_6_), 14.2 (CH_3Et_). MS (ESI) m/z calculated for [C15H23NNaO6]^+^ 336.14; found 336.18.

Compound **13c.** y = 65% ^1^H NMR (400 MHz, CDCl_3_) δ 5.99–5.86 (m, 1H, H_8_), 5.31 (dd, *J* = 17.2, 1.5 Hz, 1H, H_9a_), 5.22 (d, *J* = 10.4 Hz, 1H, H_9b_), 4.83 (s, 1H, NH_Boc_), 4.34 (dd, *J* = 6.9, 5.2 Hz, 1H, H_4_), 4.24–4.10 (m, 4H, H_7_, CH_2Et_), 3.65 (bs, 1H, H_3_), 2.78–2.71 (m, 1H, H_5_), 2.51–2.43 (m, 2H, H_6_, H_1_), 1.44 (s, 9H, CH_3Boc_), 1.28 (t, *J* = 7.1 Hz, 3H, CH_3Et_). ^13^C NMR (101 MHz, CDCl_3_) δ 202.9 (C_2_), 169.8 (CO_OEt_, CO_Boc_), 134.4 (C_8_), 117.9 (C_9_), 77.8 (C_4_), 70.6 (C_7_), 61.8 (CH_2Et_), 58.9 (C_3_), 35.7 (C_6_), 30.5 (C_5_), 28.4 (C_1_), 24.8 (CH_3Boc_), 14.3 (CH_3Et_). MS (ESI) m/z calculated for [C17H25NNaO6]^+^ 362.16; found 362.35.

Compound **13d.** y = 55% ^1^H NMR (400 MHz, CDCl_3_) δ 4.99 (s, 1H, H_9a_), 4.92 (bs, 2H, H_9b_, NH_Boc_) 4.30 (dd, *J* = 7.1, 5.1 Hz, 1H, H_4_), 4.16 (m, 2H, CH_2Et_), 4.04 (d, *J* = 2.6 Hz, 2H, H_7_), 3.68 (bs, 1H, H_3_), 2.73 (q, *J* = 5.1 Hz, 1H, H_5_), 2.57–2.37 (m, 2H, H_1_, H_6_), 1.74 (s, 3H, H_10_), 1.42 (s, 9H, Boc), 1.27 (t, *J* = 7.1 Hz, 3H, CH_3Et_). ^13^C NMR (101 MHz, CDCl_3_) δ 203.0 (C_2_), 169.9 (CO_OEt_), 155.5 (CO_Boc_), 141.8 (C_8_), 113.3 (C_9_), 77.5 (C_4_), 73.4 (C_7_), 61.8 (CH_2Et_), 58.7 (C_3_), 35.7 (C_1_), 30.4 (C_5_), 28.3 (CH_3Boc_), 24.8 (C_6_), 19.5 (C_10_), 14.2 (CH_3Et_). MS (ESI) m/z calculated for [C18H27NNaO6]^+^ 376.17; found 376.31.

Compound **13e.** ^1^H NMR (400 MHz, CDCl_3_) δ 4.76 (s, 1H, NH_Boc_), 4.19 (q, *J* = 7.1 Hz, 2H, CH_2Et_), 3.99 (s, 1H, H_4_), 3.53–3.47 (m, 1H, H_pent_), 3.39 (s, 1H, H_3_), 2.72 (bs, 1H, H_5_), 2.49–2.31 (m, 2H, H_1_, H_6_), 1.56 (s, 9H, CH_3Boc_), 1.47 (m, 4H, CH_2pent_), 1.30 (t, *J* = 7.1 Hz, 3H, CH_3Et_), 0.93 (t, *J* = 7.4 Hz, 6H, CH_2pent_), 0.87 (t, *J* = 7.5 Hz, 3H). MS (ESI) m/z calculated for [C19H31NNaO6]^+^ 392.20; found 392.50.

#### General procedure for *N*-Boc removal and *N*-acetylation starting from compounds of general formula 13

Compound **13** was dissolved in dry CH_2_Cl_2_ (conc. 0.5 M) under nitrogen atmosphere, then trifluoroacetic acid (5 equiv.) was added and the reaction mixture stirred for 5 h, until starting material consumption was observed by TLC. The reaction was cooled at 0°C and pyridine (7.5 equiv.) was added slowly, then acetic anhydride (2 equiv.) was added and the reaction mixture was stirred overnight at room temperature. The mixture was evaporated and residue purified by column chromatography to afford compound of general formula 14.

Compound **14a.** y = 69%. ^1^H NMR (400 MHz, CDCl_3_) δ 6.72 (d, *J* = 8.0 Hz, 1H, NH_Ac_), 4.27 (dd, *J* = 7.0, 5.1 Hz, 1H, H_4_), 4.22–3.99 (m, 2H, CH_2Et_), 3.79 (t, *J* = 7.0 Hz, 1H, H_3_), 3.67–3.43 (m, 2H, H_7_), 2.72 (ddd, *J* = 6.1, 5.1, 3.7 Hz, 1H, H_5_), 2.51–2.36 (m, 2H, H_1_, H_6_), 1.96 (s, 3H, CH_3Ac_), 1.18 (t, *J* = 7.1 Hz, 3H, CH_3Et_), 1.15 (t, *J* = 7.1 Hz, 3H, CH_3-8_). ^13^C NMR (101 MHz, CDCl_3_) δ 202.8 (C_2_), 170.8 (CO_Ac_), 169.8 (CO_OEt_), 77.9 (C_4_), 64.8 (CH_2Et_), 61.6 (C_7_), 57.9 (C_3_), 35.7 (C_1_), 30.4 (C_5_), 24.5 (C_6_), 22.8 (CH_3Ac_), 15.3 (CH_3Et_), 14.1 (C_8_). MS (ESI) m/z calculated for [C13H19NNaO5]^+^ 292.12; found 292.59.

Compound **14b.** y = 63%. ^1^H NMR (400 MHz, CDCl_3_) δ 7.02 (d, *J* = 8.0 Hz, 1H, NH_Ac_), 4.19–4.00 (m, 3H, H_4_, CH_2Et_), 3.82 (t, *J* = 7.5 Hz, 1H, H_3_), 3.35 (s, 3H, CH_3-7_), 2.77–2.65 (m, 1H, H_5_), 2.44–2.41 (m, 2H, H_1_, H_6_), 1.94 (s, 3H, CH_3Ac_), 1.19 (t, *J* = 7.1 Hz, 3H, CH_3Et_). ^13^C NMR (101 MHz, CDCl_3_) δ 202.8 (C_2_), 171.1 (CO_Ac_), 169.7 (CO_OEt_), 79.5 (C_4_), 61.6 (CH_3Et_), 57.5 (C_3_), 56.8 (C_7_), 35.6 (C_1_), 29.8 (C_5_), 24.4 (C_6_), 22.6 (CH_3Ac_), 14.0 (CH_3Et_). MS (ESI) m/z calculated for [C12H17NNaO5]^+^ 278.10; found 278.19.

Compound **14c.** Purification by column chromatography (Hex:EtOAc; gradient: 10–70% EtOAc in 7 CV, 7–100% EtOAc in 8 CV). y = 80%. ^1^H NMR (400 MHz, CDCl_3_) δ 6.03 (s, 1H, NH_Ac_), 5.91 (m, 1H, H_8_), 5.29 (dd, *J* = 17.2, 1.5 Hz, 1H, H_9a_), 5.25–5.17 (m, 1H, H_9b_), 4.35 (dd, *J* = 7.2, 5.1 Hz, 1H, H_4_), 4.24–4.04 (m, 4H, H_7_, CH_2Et_), 3.87 (t, *J* = 7.2 Hz, 1H, H_3_), 2.80–2.69 (m, 1H, H_5_), 2.53–2.45 (m, 2H, H_6_, H_1_), 2.03 (s, 3H, CH_3Ac_), 1.27 (t, *J* = 7.1 Hz, 3H, CH_3Et_). ^13^C NMR (101 MHz, CDCl_3_) δ 202.5 (C_2_), 170.5 (CO_Ac_), 169.8 (CO_OEt_), 134.4 (C_8_), 117.9 (C_9_), 77.9 (C_4_), 70.5 (C_7_), 61.8 (CH_2Et_), 58.1 (C_3_), 36.0 (C_1_), 30.5 (C_5_), 24.8 (C_6_), 23.0 (CH_3Ac_), 14.2 (CH_3Et_). MS (ESI) m/z calculated for [C14H19NNaO5]^+^ 304.12; found 304.21.

Compound **14d.** y = 52%. ^1^H NMR (400 MHz, CDCl_3_) δ 6.51 (d, *J* = 7.9 Hz, 1H, NH_Ac_), 4.94 (s, 1H, H_9a_), 4.88 (s, 1H, H_9b_), 4.30 (dd, *J* = 7.0, 5.1 Hz, 1H, H_4_), 4.20–4.05 (m, 2H, CH_2Et_), 4.02–3.93 (m, 2H, H_7_), 3.87 (t, *J* = 7.0 Hz, 1H, H_3_), 2.73 (ddd, *J* = 6.1, 5.1, 3.0 Hz, 1H, H_5_), 2.49 (t, *J* = 3.0 Hz, 1H, H_6_), 2.45 (dd, *J* = 6.1, 3.0 Hz, 1H, H_1_), 1.99 (s, 3H, CH_3Ac_), 1.70 (s, 3H, H_10_), 1.24 (t, *J* = 7.1 Hz, 3H, CH_3Et_). ^13^C NMR (101 MHz, CDCl_3_) δ 202.6 (C_2_), 170.8 (CO_Ac_), 169.8 (CO_OEt_), 141.8 (C_8_), 113.2 (C_9_), 77.2 (C_4_), 73.3 (C_7_), 61.7 (CH_2Et_), 57.8 (C_3_), 35.9 (C_1_), 30.4 (C_5_), 24.7(C_6_), 22.8 (CH_3Ac_), 19.4 (C_10_), 14.1 (CH_3Et_). MS (ESI) m/z calculated for [C15H21NNaO5]^+^ 318.13; found 318.37.

#### General procedure for reductive amination of compounds of general formula 14 and *N*-Boc protection

Compound **14** was dissolved in a solution of 2M NH_3_ in EtOH (5 equiv.), then Ti(OEt)_4_ (1.5 equiv.) was added and the reaction mixture stirred for 45 min at room temperature. NaBH_4_ (3 equiv.) was added in one portion and the reaction mixture was diluted to a concentration of 0.2 M with EtOH and stirred for 20 min. Boc_2_O (5 equiv.) was added and the reaction mixture was stirred for 2 h at room temperature. The mixture was quenched with water, diluted with CH_2_Cl_2_ and transferred in a separatory funnel. Saturated NaCl was added and the aqueous solution was extracted with CH_2_Cl_2_. The organic phase was dried over NaSO_4_ and evaporated. The residue purified by column chromatography to afford compound of general formula **15**.

Compound **15a.** y = 65%. ^1^H NMR (400 MHz, CDCl_3_) δ 6.43 (d, *J* = 8.6 Hz, 1H, NH_Ac_), 5.43 (d, *J* = 8.2 Hz, 1H, NH_Boc_), 4.13–4.06 (m, 3H, H_2_, CH_2Et_), 3.97–3.95 (m, 1H, H_4_), 3.72–3.68 (m, 1H, H_3_), 3.62–3.40 (m, 2H, H_7_), 2.13–2.10 (m, 3H, H_1_, H_5_, H_6_), 1.94 (s, 3H, CH_3Ac_), 1.40 (s, 9H, CH_3Boc_), 1.17 (t, *J* = 7.1 Hz, 3H, CH_3Et_), 1.14 (t, *J* = 7.1 Hz, 3H, CH_3-8_). ^13^C NMR (101 MHz, CDCl_3_) δ 172.5 (CO_Ac_), 170.9 (CO_OEt_), 156.3 (CO_Boc_), 80.7 (C_4_), 79.8 (C_Boc_), 64.9 (C_7_), 60.9 (CH_2Et_), 55.6 (C_3_), 55.0 (C_2_), 28.5 (CH_3Boc_), 28.2 (C_1_), 27.2 (C_5_), 23.4 (CH_3Ac_), 18.4 (C_6_), 15.5 (CH_3Et_), 14.2 (C_8_). MS (ESI) m/z calculated for [C16H26N2NaO6]^+^ 365.17; found 365.44.

Compound **15b.** y = 76%. ^1^H NMR (400 MHz, CDCl_3_) δ 6.41 (s, 1H, NH_Ac_), 1.40 (s, 9H, CH_3Boc_), 1.17 (t, *J* = 7.1 Hz, 3H, CH_3Et_), 1.14 (t, *J* = 7.1 Hz, 3H, CH_3-8_), 5.41 (d, *J* = 7.8 Hz, 1H, NH_Boc_), 4.23–3.99 (m, 3H, H_2_, CH_2Et_), 3.94–3.81 (m, 1H, H_4_), 3.81–3.66 (m, 1H, H_3_), 3.36 (s, 3H, H_7_), 2.23–2.06 (m, 3H, H_1_, H_5_, H_6_), 1.95 (s, 3H, CH_3Ac_), 1.41 (s, 9H, CH_3Boc_), 1.24 (t, *J* = 7.1 Hz, 3H, CH_3Et_). ^13^C NMR (101 MHz, CDCl_3_) δ 172.3 (CO_Ac_), 171.0 (CO_OEt_), 156.2 (CO_Boc_), 82.5 (C_4_), 80.0 (C_Boc_), 61.0 (CH_2Et_), 57.3 (C_7_), 55.6 (C_3_), 54.9 (C_2_), 29.8 (CH_3Boc_), 28.5 (C_1_), 26.5 (C_5_), 23.5 (CH_3Ac_), 18.3 (C_6_), 14.3 (CH_3Et_).

Compound **15c.** y = 72%. ^1^H NMR (400 MHz, CDCl_3_) δ 6.71 (d, *J* = 8.8 Hz, 1H, NH_Ac_), 5.90–5.84 (m, 1H, H_8_), 5.55 (d, *J* = 8.5 Hz, 1H, NH_Boc_), 5.22 (dd, *J* = 17.2, 1.6 Hz, 1H, H_9a_), 5.16–5.08 (m, 1H, H_9b_), 4.22–4.11 (m, 1H, H_2_), 4.11–3.93 (m, 5H, CH_2Et_, H_4_, H_7_), 3.78–3.74 (m, 1H, H_3_), 2.23–2.02 (m, 3H, H_1_, H_5_, H_6_), 1.92 (s, 3H, CH_3Ac_), 1.39 (s, 9H, CH_3Boc_), 1.21 (t, *J* = 7.1 Hz, 3H, (CH_3Et_). ^13^C NMR (101 MHz, CDCl_3_) δ 172.4 (CO_Ac_), 170.7 (CO_OEt_), 156.2 (CO_Boc_), 134.8 (C_8_), 117.1 (C_9_), 80.3 (C_4_), 79.6 (C_Boc_), 70.3 (C_7_), 60.7 (CH_2Et_), 55.4 (C_3_), 54.7 (C_2_), 29.6 (CH_3Boc_), 28.4 (C_1_), 27.2 (C_5_), 23.2 (CH_3Ac_), 18.3 (C_6_), 14.1 (CH_3Et_). MS (ESI) m/z calculated for [C19H31N2O6]^+^ 383.22; found 383.04; m/z calculated for [C19H30N2NaO6]+ 405.20; found 405.20.

Compound **15d.** y = 62%. ^1^H NMR (400 MHz, CDCl_3_) δ 6.76 (d, *J* = 8.6 Hz, 1H, NH_Ac_), 5.54 (d, *J* = 8.4 Hz, 1H), 4.91 (s, 1H, H_9a_), 4.83 (s, 1H, H_9b_), 4.20–4.11 (m, 1H, H_2_), 4.11–4.02 (m, 2H, CH_2Et_), 4.01–3.83 (m, 3H, H_4_, H_7_), 3.77 (m, 1H, H_3_), 2.17–2.02 (m, 3H, H_1_, H_5_, H_6_), 1.91 (s, 3H, CH_3Ac_), 1.67 (s, 3H, H_10_), 1.38 (s, 9H, CH_3Boc_), 1.21 (t, *J* = 7.1 Hz, 3H, CH_3Et_). ^13^C NMR (101 MHz, CDCl_3_) δ 172.5 (CO_Ac_), 170.8 (CO_OEt_),156.3 (CO_Boc_), 142.3 (C_8_), 112.6 (C_9_), 80.3 (C_4_), 79.6 (C_Boc_), 73.4 (C_7_), 60.8 (CH_2Et_), 55.4 (C_3_), 54.6 (C_2_), 28.4 (CH_3Boc_), 27.2 (C_1_, C_5_), 23.3 (C_6_), 19.4 (CH_3Ac_), 18.4 (C_10_), 14.2 (CH_3Et_). MS (ESI); m/z calculated for [C20H32N2NaO6]^+^ 419.22; found 419.34.

Compound **15f**. Compound **14** was dissolved in a solution of 2 M NH_3_ in EtOH (5 equiv.), then Ti(OEt)_4_ (4 equiv.) was added and the reaction mixture stirred for 45 min at room temperature. NaBH_4_ (3 equiv.) was added in one portion and the reaction mixture was diluted to a concentration of 0.2 M with EtOH and stirred for 1h at room temperature. The mixture was quenched with water, diluted with CH_2_Cl_2_ and transferred in a separatory funnel. Saturated NaCl was added and the aqueous solution was extracted with CH_2_Cl_2_. The organic phase was dried over NaSO_4_ and evaporated. The residue purified by column chromatography to afford compound **15f**. ^1^H NMR (400 MHz, CDCl_3_) δ 6.36 (d, *J* = 3.0 Hz, 1H, NH_Ac_), 5.88-5-82 (m 1H, H_8_), 5.32–5.22 (m, 1H, H_9a_), 5.22–5.14 (m, 2H, H_9b_, OH), 4.24–4.17 (m, 1H, H_2_), 4.16–4.05 (m, 3H, CH_2Et_, H_7a_), 4.05–3.95 (m, 2H, H_4_; H_7b_), 3.30 (td, *J* = 7.3, 4.1 Hz, 1H, H_3_), 2.16–2.12 (m, 3H; H_1_, H_5_, H_6_), 1.98 (s, 3H, CH_3Ac_), 1.23 (t, *J* = 7.1 Hz, 3H, CH_3Et_). ^13^C NMR (101 MHz, CDCl_3_) δ 173.2 (CO_Ac_), 172.6 (CO_OEt_), 134.5 (C_8_), 117.8 (C_9_), 80.3 (C_4_), 75.0 (C_2_), 70.4 (C_7_), 61.2 (C_3_), 61.0 (CH_2Et_), 30.1 (C_5_), 27.6 (C_1_), 23.0 (CH_3Ac_), 18.7 (C_6_), 14.2 (CH_3Et_). MS (ESI) m/z calculated for [C14H22NO5]^+^ 284.15; found 284.15; m/z calculated for [C14H21NNaO5]^+^ 306.13; found 306.23.

#### General procedure for osmium-catalyzed dihydroxylation

Compound **15c** (or **18c**) (1 equiv.) was dissolved in dry acetone (c = 0.08 M) under nitrogen. Trimethylamine n-oxide dihydrate (1.8 equiv.) and osmium(III) chloride (0.05 equiv.) were added and the reaction mixture stirred for 2 h at room temperature. Completion of reaction was monitored by ^1^H-NMR. Na_2_S_2_O_3_ was added and the reaction mixture was stirred for 5 minutes, then filtered on a celite pad. The solvent was evaporated and the residue was purified by C18 column chromatography (water:acetonitrile; gradient: 10–70% acetonitrile in 15 CV).

Compound **15e.** y = 95%. ^1^H NMR (400 MHz, Methanol-*d*_4_) δ 4.20–4.09 (m, 3H, CH_2Et_, H_2_), 4.07 (dd, *J* = 7.5, 3.8 Hz, 1H, H_4_), 3.78–3.70 (m, 1H, H_8_), 3.70–3.38 (m, 5H, H_3_, H_7_, H_9_), 2.24–2.15 (m, 2H, H_1_, H_6_), 2.14–2.07 (m, 1H, H_5_), 1.95 (s, 3H, CH_3Ac_), 1.45 (s, 9H, CH_3Boc_), 1.28 (t, *J* = 7.1 Hz, 3H, CH_3Et_). ^13^C NMR (101 MHz, Methanol-*d*_4_) δ 174.2 (CO_Ac_),173.5 (CO_OEt_), 158.1 (CO_Boc_), 83.0 (C_4_), 82.9 (C_4_), 80.3 (C_Boc_), 72.3 (C_8_), 72.2 (C_8_), 72.1 (C_9_), 71.9 (C_9_), 64.4 (C_7_), 64.3 (CH_2Et_), 62.0 (C_3_), 56.3 (C_2_), 55.5 (C_2_), 29.6 (C_5_), 28.7 (CH_3Boc_), 28.3 (C_1_), 22.8 (CH_3Ac_), 19.3 (C_6_), 14.5 (CH_3Et_).

Compound **15g.** y = 95%. ^1^H NMR (400 MHz, Methanol-*d*_4_) δ 4.18 (dd, *J* = 7.8, 4.7 Hz, 1H, H_2_), 4.15–4.07 (m, 2H, CH_2Et_), 4.00 (dd, *J* = 7.7, 3.6 Hz, 1H, H_4_), 3.75–3.68 (m, 1H, H_8_), 3.64–3.47 (m, 5H, H_7_, H_9_, H_3_), 2.20–2.15 (m, 2H, H_1_, H_6_), 2.10–2.04 (m, 1H, H_5_), 1.96 (s, 3H, CH_3Ac_), 1.25 (t, *J* = 7.1 Hz, 3H, CH_3Et_). ^13^C NMR (101 MHz, Methanol-*d*_4_) δ 174.4 (CO_Ac_), 173.8 (CO_OEt_), 82.6 (C_4_), 74.8 (C_2_), 72.3 (C_8_), 71.9 (C_9_), 64.4 (C_7_), 64.3 (CH_2Et_), 59.3 (C_3_), 31.1 (C_5_), 28.5 (C_1_), 22.8 (CH_3Ac_), 19.5 (C_6_), 14.5 (CH_3Et_).

#### General procedure for the synthesis of amino acids of general formula 16

Compound **15** (1 equiv.) was dissolved in a 1:5 mixture of water and methanol (at a compound concentration of 0.05 M) at 0°C. Next, 5 equivalents of a cold solution of NaOH 6 M was added and the mixture was kept at 4°C overnight. The solution was diluted with water and extracted once with EtOAc. The water layer was acidified with 0.5 M HCl and extracted with EtOAc (3 times). Organic extracts were dried over NaSO_4_, evaporated at reduced pressure. The obtained solid was dissolved in 1:3 mixture of TFA and dichloromethane (at a compound concentration of 0.05 M) and stirred at room temperature for 2 h. Cold diethylether was added to allow the precipitation of the product, which was triturated, precipitated by centrifugation and the solution removed (the procedure was repeated 3 times). The white solid was then dissolved in water and freeze-dried to give a white foam.

Compound **16a**. y = quant. ^1^H NMR (400 MHz, D_2_O) δ 4.32 (dd, *J* = 7.6, 4.6 Hz, 1H, H_4_), 3.89–3.75 (m, 2H, H_2_, H_3_), 3.75–3.67 (m, 1H, H_7a_), 3.65–3.52 (m, 1H, H_7b_), 2.44 (ddd, *J* = 7.5, 4.6, 3.3 Hz, 1H, H_5_), 2.30–2.21 (m, 2H, H_1_, H_6_), 1.99 (s, 3H, CH_3Ac_), 1.17 (t, *J* = 7.1 Hz, 3H, CH_3-8_). ^13^C NMR (101 MHz, D_2_O) δ 175.6 (COOH), 175.0 (CO_Ac_), 80.2 (C_4_), 65.8 (C_7_), 54.9 (C_2_), 54.0 (C_3_), 27.7 (C_5_), 25.5 (C_1_), 21.7 (CH_3Ac_), 18.7 (C_6_), 14.3 (C_8_). HRMS (ESI) m/z calculated for [C11H18N2NaO4]^+^ 265.11588; found 265.11633.

Compound **16b**. y = quant. ^1^H NMR (400 MHz, D_2_O) δ 4.14 (dd, *J* = 7.6, 4.6 Hz, 1H, H_4_), 3.82–3.67 (m, 2H, H_2_, H_3_), 3.34 (s, 3H, CH_3-7_), 2.42–2.33 (m, 1H, H_5_), 2.20–2.16 (m, 1H, H_1_), 2.14 (t, *J* = 3.1 Hz, 1H, H_6_), 1.91 (s, 3H, CH_3Ac_). ^13^C NMR (101 MHz, D_2_O) δ 175.5 (COOH), 175.0 (CO_Ac_), 81.9 (C_4_), 57.0 (C_7_), 54.9 (C_2_), 53.9 (C_3_), 27.1 (C_5_), 25.5 (C_1_), 21.7 (CH_3Ac_), 18.6 (C_6_). HRMS (ESI) m/z calculated for [C10H15N2O4]^-^ 227.10373; found 227.10360.

Compound **16c**. y = quant. ^1^H NMR (400 MHz, D_2_O) δ 5.99–5.87 (m, 1H, H_8_), 5.34 (dq, *J* = 17.3, 1.5 Hz, 1H, H_9a_), 5.27 (dd, *J* = 10.4, 1.4 Hz, 1H, H_9b_), 4.37 (dd, *J* = 6.8, 5.2 Hz, 1H, H_4_), 4.22–4.07 (m, 2H, H_7_), 3.89–3.77 (m, 2H, H_3_, H_2_), 2.47–2.40 (m, 1H, H_5_), 2.30–2.22 (m, 2H, H_1_, H_6_), 2.01 (s, 3H, CH_3Ac_). ^13^C NMR (101 MHz, D_2_O) δ 176.2 (COOH), 175.6 (CO_Ac_), 134.3 (C_8_), 119.3 (C_9_), 80.8 (C_4_), 71.6 (C_7_), 55.5 (C_2_), 54.7 (C_3_), 28.5 (C_5_), 26.4 (C_1_), 22.5 (CH_3-Ac_), 19.4 (C_6_). HRMS (ESI) m/z calculated for [C12H17N2O4]^-^ 253.11938; found 253.11922.

Compound **16d**. y = quant. ^1^H NMR (400 MHz, D_2_O) δ 4.94–4.88 (m, 2H, H_9_), 4.28–4.19 (m, 1H, H_4_), 3.99–3.95 (m, 2H, H_7_), 3.79–3.72 (m, 2H, H_2_, H_3_), 2.43–2.32 (m, 1H, H_1_), 2.23–2.15 (m, 2H, H_5_, H_6_), 1.91 (s, 3H, CH_3Ac_), 1.62 (s, 3H, CH_3-10_). ^13^C NMR (101 MHz, D_2_O) δ 175.2 (COOH), 174.8 (CO_Ac_), 141.8 (C_8_), 113.7 (C_9_), 81.3 (C_4_), 74.0 (C_7_), 54.6 (C_3_), 53.9 (C_2_), 27.9 (C_1_, C_5_), 25.7 (CH_3Ac_), 21.7 (C_6_), 18.4 (C_10_). HRMS (ESI) m/z calculated for [C13H20N2NaO4]^+^ 291.13153; found 291.13212.

Compound **16e.** y = quant. ^1^H NMR (400 MHz, D_2_O) δ 4.19 (ddd, *J* = 7.3, 4.6, 2.6 Hz, 1H, H_4_)), 3.79–3.64 (m, 3H, H_2_, H_3_; H_8_), 3.59 (dd, *J* = 10.4, 4.0 Hz, 1H, H_7_), 3.54–3.34 (m, 3H, H_9_, H_7_), 2.34 (dt, *J* = 7.3, 4.0 Hz, 1H, H_5_), 2.22–2.10 (m, 2H, H_1_, H_6_), 1.86 (s, 3H, CH_3Ac_). ^13^C NMR (101 MHz, D_2_O) δ 175.3 (COOH), 175.0 (CO_Ac_), 81.2 (C_4_), 81.1 (C_4_), 70.6 (C_8_), 70.5 (C_8_), 70.4 (C_9_), 70.4 (C_9_), 62.4 (C_7_), 62.4 (C_7_), 54.8 (C_2_), 54.7 (C_2_), 54.1 (C_3_), 27.7 (C_5_), 27.6 (C_5_), 25.7 (C_1_), 21.7 (CH_3Ac_), 18.5 (C_6_). HRMS (ESI) m/z calculated for [C12H19N2O6]^-^ 287.12486; found 287.12487.

Compound **16f.** y = quant. ^1^H NMR (400 MHz, D_2_O) δ 4.27 (dd, *J* = 8.0, 4.4 Hz, 1H, H_4_), 4.11 (dd, *J* = 7.8, 4.4 Hz, 1H, H_2_), 3.89–3.78 (m, 1H, H_3_), 3.73–3.45 (m, 5H, H_7_, H_9_, H_8_), 2.37–2.27 (m, 1H, H_5_), 2.27–2.17 (m, 2H, H_1_, H_6_), 2.00 (s, 3H, CH_3Ac_). ^13^C NMR (101 MHz, D_2_O) δ 176.5 (COOH), 174.3 (CO_Ac_), 80.9 (C_4_), 73.2 (C_2_), 73.1 (C_8_), 70.2 (C_9_), 62.5 (C_7_),56.7 (C_3_), 29.5 (C_5_)27.4 (C_1_), 22.1 (CH_3Ac_), 18.1 (C_6_). HRMS (ESI) m/z calculated for [C12H18N1O7]^+^ 288.10888; found 288.10854.

#### General procedure for guanylation starting from compounds 16

To a solution of compound 16 in CH_2_Cl_2_ (0.2M), trifluoroacetic acid (4.4 equiv) was added. After 6 h, the reaction mixture was cooled at 0°C and triethylamine (6.8 equiv) was added dropwise, followed by the addition of *N*,*N'-*bis(tert-butoxycarbonyl)thiourea (1.2 equiv) and Mukaiyama’s reagent (1.2 equiv). The reaction was allowed to stir at 25°C until completion, then the solvent was evaporated. The crude material was dissolved in diethyl ether and washed with water. The organic layer was dried over NaSO_4_ and evaporated. The crude was purified by flash chromatography.

Compound **18a.** y = 60%. ^1^H NMR (400 MHz, CDCl_3_) δ 11.31 (s, 1H, NH_Boc_), 8.58 (d, *J* = 7.8 Hz, 1H, NH_(H2)_), 6.37 (d, *J* = 8.5 Hz, 1H, NH_Ac_), 4.68 (ddd, *J* = 8.5, 7.8, 4.2 Hz, 1H, H_2_), 4.13–4.00 (m, 2H, CH_2Et_), 3.97–3.92 (m, 1H, H_4_), 3.65 (q, *J* = 8.5 Hz, 1H, H_3_), 3.59–3.40 (m, 2H, H_7_), 2.20–2.06 (m, 3H, H_1_, H_5_, H_6_), 1.90 (s, 3H, CH_3Ac_), 1.45 (s, 9H, CH_3Boc_), 1.41 (s, 9H, CH_3Boc_), 1.25–1.18 (m, 3H, CH_3Et_), 1.18–1.07 (m, 3H, CH_3-8_). ^13^C NMR (101 MHz, CDCl_3_) δ 172.2 (CO_Ac_), 170.8 (CO_OEt_), 163.1(C_N_), 156.7 (CO_Boc_), 152.7 (CO_Boc_), 83.5 (C_Boc_), 81.3 (C_4_), 79.3 (C_Boc_), 64.9 (C_7_), 60.9 (CH_2Et_), 56.5 (C_3_), 53.9 (C_2_), 28.3 (CH_3Boc_), 28.1 (CH_3Boc_), 27.7(C_1_), 27.4 (C_5_), 23.4 (CH_3Ac_), 18.4 (C_6_), 15.4 (CH_3Et_), 14.1(CH_3-8_). MS (ESI) m/z calculated for [C24H41N4O8]^+^ 513.29; found 513.46.

Compound **18b**. y = 65%. ^1^H NMR (400 MHz, CDCl_3_) δ 11.33 (s, 1H, NH_Boc_), 8.61 (d, *J* = 7.5 Hz, 1H, NH_(H2)_), 6.37 (d, *J* = 7.8 Hz, 1H, NH_Ac_), 4.75–4.71 (m, 1H, H_2_), 4.11 (q, *J* = 7.2 Hz, 2H, CH_2Et_), 3.85 (dd, *J* = 7.8, 3.3 Hz, 1H, H_4_), 3.67 (q, *J* = 7.8 Hz, 1H, H_3_), 3.36 (s, 3H, CH_3-7_), 2.26–2.08 (m, 3H, H_1_, H_5_; H_6_), 1.92 (s, 3H, CH_3Ac_), 1.47 (s, 9H, CH_3Boc_), 1.44 (s, 9H, CH_3Boc_), 1.25 (t, *J* = 7.2 Hz, 3H, CH_3Et_). ^13^C NMR (101 MHz, CDCl_3_) δ 172.2 (CO_Ac_), 170.9 (CO_OEt_), 163.0 (C_N_), 156.7 (CO_Boc_), 152.7 (CO_Boc_), 83.6 (C_Boc_), 83.2 (C_4_), 79.5 (C_Boc_), 61.1 (CH_2Et_), 57.3 (C_7_), 56.4 (C_3_), 53.8 (C_2_), 28.3 (CH_3Boc_), 28.1 (CH_3Boc_), 27.3 (C_1_), 27.0 (C_5_), 23.4 (CH_3Ac_), 18.4 (C_6_), 14.2 (CH_3Et_). MS (ESI) m/z calculated for [C23H39N4O8]+ 499.27; found 499.58.

Compound **18c.** y = 66%. ^1^H NMR (400 MHz, CDCl_3_) δ 11.33 (s, 1H, NH_Boc_), 8.60 (d, *J* = 7.5 Hz, 1H, NH_(H2)_), 6.26 (d, *J* = 8.0 Hz, 1H, NH_Ac_), 5.89–5.84 (m, 1H, H_8_), 5.24 (dd, *J* = 17.3, 1.6 Hz, 1H, H_9a_), 5.17–5.11 (m, 1H, H_9b_), 4.74–4.72 (m, 1H, H_2_), 4.14–3.96 (m, 5H, H_7_, CH_2Et_, H_4_), 3.69 (q, *J* = 8.0 Hz, 1H, H_3_), 2.23–2.08 (m, 3H, H_1_, H_5_, H_6_), 1.93 (s, 3H, CH_3Ac_), 1.48 (s, 9H, CH_3Boc_), 1.45 (s, 9H, CH_3Boc_), 1.25 (t, *J* = 7.1 Hz, 3H, CH_3Et_). ^13^C NMR (101 MHz, CDCl_3_) δ 172.2 (CO_Ac_), 170.8 (CO_OEt_), 163.1 (C_N_), 156.8 (CO_Boc_), 152.8 (CO_Boc_), 134.9 (C_8_), 117.2 (C_9_), 83.6 (C_Boc_), 81.1 (C_4_), 79.4 (C_Boc_), 70.5 (C_7_), 61.0 (CH_2Et_), 56.8 (C_3_), 53.9 (C_2_), 28.4 (CH_3Boc_), 28.2 (CH_3Boc_), 27.7 (C_1_), 27.5 (C_5_), 23.5 (CH_3Ac_), 18.5 (C_6_), 14.2 (CH_3Et_).

Compound **18d.** y = 53%. ^1^H NMR (400 MHz, CDCl_3_) δ 11.35 (s, 1H, NH_Boc_), 8.65 (d, *J* = 7.3 Hz, 1H, NH_(H2)_), 6.22 (d, *J* = 6.9 Hz, 1H, NH_Ac_), 4.94 (s, 1H, H_9a_), 4.87 (s, 1H, H_9b_), 4.84–4.73 (m, 1H, H_2_), 4.19–4.07 (m, 2H, CH_2Et_), 4.01–3.93 (m, 3H, H_7_, H_2_), 3.74–3.70 (m, 1H, H_3_), 2.24–2.12 (m, 3H, H_1_, H_5_, H_6_), 1.94 (s, 3H, CH_3-10_), 1.71 (s, 3H, CH_3Ac_), 1.48 (s, 9H, CH_3Boc_), 1.46 (s, 9H, CH_3Boc_), 1.27 (t, *J* = 7.2 Hz, 3H, CH_3Et_). ^13^C NMR (101 MHz, CDCl_3_) δ 172.2 (CO_Ac_), 172.0 (CO_OEt_), 170.7 (C_N_), 156.6 (CO_Boc_), 152.7 (CO_Boc_), 142.3 (C_8_), 113.3 (C_9_), 80.8 (C_4_), 73.5 (C_7_), 61.0 (CH_2Et_), 56.6 (C_3_), 53.6 (C_2_), 28.3 (CH_3Boc_), 28.1 (CH_3Boc_), 27.5 (C_1_), 27.3 (C_5_), 23.4 (CH_3Ac_), 19.4 (C_6_), 18.4 (C_10_), 14.2 (CH_3Et_). MS (ESI) m/z calculated for [C26H42N4NaO8]^+^ 561.29; found 561.76.

Compound **18e.** y = 44%. ^1^H NMR (400 MHz, CDCl_3_) δ 11.33 (s, 1H, NH_Boc_), 8.66 (d, *J* = 6.4 Hz, 1H, NH_(H2)_), 7.62 (d, *J* = 5.4 Hz, 1H, NH_Ac_), 7.51 (d, *J* = 6.0 Hz, 1H, NH_Ac_), 4.82–4.71 (m, 1H, H_2_), 4.14 (q, *J* = 7.1 Hz, 2H, CH_2Et_), 4.02–3.98 (m, 1H, H_4_), 3.87–3.74 (m, 2H, H_8_; H_7a_), 3.74–3.36 (m, 4H, H_7b_, H_9_, H_3_), 2.24–2.10 (m, 3H, H_1_; H_5_, H_6_), 1.95 (s, 3H, CH_3Ac_), 1.49 (s, 9H, CH_3Boc_), 1.46 (s, 9H, CH_3Boc_), 1.31–1.20 (m, 3H, CH_3Et_). ^13^C NMR (101 MHz, CDCl_3_) δ 172.0 (CO_Ac_), 171.9 (CO_Ac_), 171.8 (CO_OEt_), 162.7 (C_N_), 162.6 (C_N_), 157.0 (CO_Boc_), 157.0 (CO_Boc_), 152.8 (CO_Boc_), 84.1 (C_4_), 84.0 (C_Boc_), 84.0 (C_Boc_), 83.3 (C_4_), 79.9 (C_Boc_), 70.6 (C_9_), 70.2 (C_8_), 63.6 (C_7_), 63.5 (C_7_), 61.3 (CH_2Et_), 58.4 (C_3_), 57.9 (C_3_), 53.6 (C_2_), 28.3 (CH_3Boc_), 28.2 (CH_3Boc_), 27.8 (C_1_), 27.7 (C_1_), 27.4 (C_5_), 27.2 (C_5_), 23.5 (CH_3Ac_), 18.7 (C_6_)14.2 (CH_3Et_).

#### General procedure for the synthesis of compounds 19

Compound 18 (1 equiv.) was dissolved in a 1:5 mixture of water and methanol (at a compound concentration of 0.05 M) at 0°C. Next, 5 equivalents of a cold solution of NaOH 6 M was added and the mixture was kept at 4°C overnight. The water layer was diluted with water and acidified with a solution of HCl 6 M (5 equiv.), while stirring at 0°C. The solution was freeze-dried and the obtained solid was dissolved in 1:3 mixture of TFA and dichloromethane (at a compound concentration of 0.05 M) and stirred at room temperature for 2 h. Cold diethylether was added to allow the precipitation of the product, which was triturated, precipitated by centrifugation and the solution removed (the procedure was repeated 3 times). The white solid was then dissolved in water and freeze-dried to give a white foam.

Compound **19a.** y = 90%. ^1^H NMR (400 MHz, D_2_O) δ 4.24 (dd, *J* = 8.1, 4.5 Hz, 1H, H_4_), 4.12 (dd, *J* = 8.7, 4.4 Hz, 1H, H_2_), 3.78–3.65 (m, 2H, H_3_; H_7a_), 3.64–3.49 (m, 1H, H_7b_), 2.40–2.32 (m, 1H, H_5_), 2.32–2.25 (m, 1H, H_1_), 2.17 (t, *J* = 3.0 Hz, 1H, H_6_), 1.97 (s, 3H, CH_3Ac_), 1.16 (t, *J* = 7.1 Hz, 3H, CH_3Et_). ^13^C NMR (101 MHz, D_2_O) δ 176.1 (COOH), 174.4 (CO_Ac_),156.7 (C_N_), 79.7 (C_4_), 65.7 (C_7_), 55.3 (C_2_), 55.2 (C_3_), 27.3 (C_5_), 27.1 (C_1_), 21.9 (CH_3Ac_), 18.3 (C_6_), 14.3 (C_8_). HRMS (ESI) m/z calculated for [C12H19N4O4]^-^ 283.14118; found 283.14102.

Compound **19b.** y = 94%. ^1^H NMR (400 MHz, D_2_O) δ 4.07–4.03 (m, 2H, H_4_; H_2_), 3.65 (t, *J* = 8.3 Hz, 1H, H_3_), 3.32 (s, 3H, CH_3-7_), 2.30 (ddd, *J* = 7.3, 4.4, 3.2 Hz, 1H; H_5_), 2.21 (ddd, *J* = 7.3, 4.4, 3.2 Hz, 1H, H_1_), 2.06 (t, *J* = 3.2 Hz, 1H, H_6_), 1.88 (s, 3H, CH_3Ac_). ^13^C NMR (101 MHz, D_2_O) δ 176.1 (COOH), 174.4 (CO_Ac_), 156.7 (C_N_), 81.4 (C_4_), 56.8 (CH_3-7_), 55.3 (C_2_), 55.1 (C_3_), 27.3 (C_1_), 26.5 (C_5_), 21.9 (CH_3Ac_), 18.2 (C_6_). HRMS (ESI) m/z calculated for [C11H17N4O4]^-^ 269.12553; found 269.12539.

Compound **19c.** y = 88%. ^1^H NMR (400 MHz, D_2_O) δ 6.04–5.83 (m, 1H, H_8_), 5.45–5.19 (m, 2H, H_9_), 4.33 (dd, *J* = 8.1, 4.5 Hz, 1H, H_4_), 4.25–4.04 (m, 3H, H_7_, H_2_), 3.78 (t, *J* = 8.1 Hz, 1H, H_3_), 2.41–2.37 (m, 1H, H_5_), 2-36-2.30 (m 1H, H_1_), 2.21 (t, *J* = 3.0 Hz, 1H, H_6_), 2.00 (s, 3H, CH_3Ac_). ^13^C NMR (101 MHz, D_2_O) δ 176.7 (COOH), 175.1 (CO_Ac_),157.4 (C_N_), 134. 4 (C_8_), 119.2 (C_9_), 80.3 (C_4_), 71.4 (C_7_), 56.0 (C_2_, C_3_), 28.2 (C_5_), 27.9 (C_1_), 22.6 (CH_3Ac_), 19.0 (C_6_). HRMS (ESI) m/z calculated for [C13H21N4O4]^+^ 297.15573; found 297.15603.

Compound **19d**. y = 82%. ^1^H NMR (400 MHz, CDCl_3_) δ 11.35 (s, 1H, NH), 8.64 (s, 1H, NH_(H2)_), 6.21 (s, 1H, NH_Ac_), 4.95 (s, 1H, H_9a_), 4.87 (s, 1H, H_9b_), 4.82–4.72 (m, 1H, H_4_), 4.20–4.06 (m, 1H, H_2_), 4.02–3.88 (m, 2H, H_7_), 3.79–3.69 (m, 1H, H_3_), 2.26–2.11 (m, 3H, H_1_; H_5_, H_6_), 1.94 (s, 3H, CH_3Ac_), 1.71 (s, 3H, CH_3-10_). ^13^C NMR (101 MHz, CDCl_3_) δ 172.3 (COOH), 170.8 (CO_Ac_), 156.7 (C_N_), 142.4 (C_8_), 112.7 (C_9_), 80.9 (C_4_), 73.6 (C_7_), 56.7 (C_2_), 53.7 (C_3_), 27.6 (C_5_), 27.4 (C_1_), 23.5 (CH_3Ac_), 19.5 (C_6_), 18.6 (CH_3-10_).

Compound **19e**. y = 77% ^1^H NMR (400 MHz, D_2_O) δ 4.15 (ddd, *J* = 7.9, 4.5, 1.5 Hz, 1H, H_4_), 4.05 (dd, *J* = 8.7, 4.4 Hz, 1H, H_2_), 3.81–3.72 (m, 1H, H_8_), 3.72–3.58 (m, 2H; H_3_; H_7a_), 3.58–3.48 (m, 2H, H_7b_, H_9_), 3.48–3.37 (m, 2H; H_9_), 2.37–2.25 (m, 1H; H_5_), 2.25–2.16 (m, 1H, H_1_), 2.12 (t, *J* = 3.1 Hz, 1H, H_6_), 1.89 (s, 3H, CH_3Ac_). ^13^C NMR (101 MHz, D_2_O) δ 175.9 (COOH), 174.4 (CO_Ac_), 156.7 (C_N_), 80.7 (C_4_), 80.7 (C_4_), 70.4 (C_8_), 70.4 (C_9_), 62.4 (C_7_), 62.4 (C_7_), 55.2 (C_2_), 55.2 (C_3_), 27.4 (C_5_), 27.1 (C_1_), 21.9 (CH_3Ac_), 18.1 (C_6_). HRMS (ESI) m/z calculated for [C13H23N4O6]^+^ 331.16121.; found 331.16121.

### Computational studies

For modeling complexes between ligands and neuraminidase N1 we used two models, one based on PDB entry 2HU4 with absence of the 150 cavity and one based on PDB entry 2HU0 with open access to the 150 cavity.[32] The Protein Preparation Wizard (PPW)^16^ was used for curating original PDB entries.[33] The first pre-processing step PPW automatically assigned bond orders of the ligand and add hydrogens to the protein. Second, the Epik tool was used for estimation of the optimal protonation of the bounded ligand. In the final refinement step of PPW the protein’s sidechains in ligand’s neighborhood are flipped so to optimize the number of hydrogen bonding contacts. The complex was finally subjected to restrained minimization using force field OPLS_2005.[34] In the case of PDB entry 2HU4, the Glide XP docking protocol with standard settings was used. The Schrödinger’s Induced Fit docking protocol has been used in the case of initial docking of compound **4**, (R = 4-phenylbenzyl). The model of the receptor was based on PDB entry 2HU0,[32] which represents a complex between neuraminidase N1 and oseltamivir. The Induced Fit docking provided by Schrödinger Inc. was started by docking of the ligand with Glide XP.[35]–[36] In order to provide the receptor site plasticity, the procedure used reduced van der Waals radii, which were scaled by factor 0.5 for both ligand and receptor. Up to 20 “soft docked” poses were then forwarded to the second step, which used a Prime structure prediction for fine tuning ligand pose within receptor by reorienting nearby side chains and subsequent minimization. In third step, each ligand is re-docked into its corresponding low energy protein structures and the resulting complexes were ranked according to Glide Score. Maestro GUI of the Schrödinger’s Suite has also been used for depiction of representation poses of ligands within binding pocket of N1. Python script complex2img.py based on OpenEye’s OEChem Toolkit has been used for creating interaction diagrams between N1 and ligands.[37]

### Crystal structure of 15c

Large crystals of compound **15c** were obtained in a sample tube by slow evaporation from acetone at *T* = 4°C. Crystallization took approximately 24 h, after which a colorless transparent prism with dimensions ≈ 0.425 x 0.125 x 0.075 mm was selected for the single crystal X-ray diffraction analysis.

The sample showed pleochroism (from colorless to purple) under polarized light. It was cut from a greater crystal using a blade and polished by mechanical ablation in a drop of perfluorinated oil. Then, it was mounted on a glass capillary fiber with perfluorinated oil as glue. The X-ray data collection was performed with a three-circle Bruker Smart APEX diffractometer equipped with an APEXII CCD detector. Graphite-monochromated Mo Kα radiation was employed throughout in conjunction with a nominal X-ray power of 50 kV x 30 mA. Raw data were processed by means of the SAINT+ software[38] and corrected for beam anisotropy by SADABS.[39] Finally, data were scaled and analyzed by XPREP[40] to determine the correct space group symmetry. Eventually, a 99.8% complete set of 26608 diffraction amplitudes (4883 unique) was measured at room temperature up to a maximum Bragg angle of 54.9 deg. The spherical atom formalism as implemented in SHELXL[41] was exploited to extract from the diffraction data a sensible molecular model through nonlinear least-square refinements. The agreement factors for the final least-squares model were R1(F) = 0.0660 for 2647 intense reflections (F_o_> 4σ(F_o_)), and 0.1261 for all the measured data, whereas the maximum and minimum Fourier residuals in the unit cell were as low as +0.35 /–0.20 e·Å^–3^. Crystal data of **15c**: C_19_H_30_N_2_O_6_, M = 382.45 amu, monoclinic, space group P2_1_/c, n° 14, centric, a = 21.2478(8) Å, b = 5.1264(2) Å, c = 21.9154(8) Å, β = 116.550(2) deg, V = 2135.4(2) Å^3^, Z = 4, Z’ = 1; ρ_calcd_ = 1.190 g·cm^–3^, μ = 0.09 mm^–1^. CCDC 1562937 contains the supplementary crystallographic data for this paper.[42] Full details of the X-ray structure determination are provided in the Supporting Information.

### Determination of NA Km and compounds Ki values in enzyme inhibition experiments

A/Chicken/HongKong/G9/1997 H9N2 and A/Anhui/1/2005 H5N1 (wild-type and H274Y mutation) were purchased from SinoBiochemical. Oseltamivir free acid and the substrate 2-(4-methylumbelliferyl) α-d-acetylneuraminic acid sodium salt hydrate (MUNANA) were purchased from Toronto Research Chemicals. The Michaelis constant, *K*_m_, for MUNANA was obtained by measuring the initial slopes (Fluorescent Unit/min; FU/min) plotted as a function of substrate concentration ranging from 2 μM to 1 mM in MES buffer (32.5 mM 2-(*N*-morpholino)ethanesulfonic acid, 4 mM CaCl_2_, pH 6.5) and fit using a nonlinear regression function in GraphPad Prizm. The NA inhibition assay was performed according to a standard method,[43] with minor modifications. Accordingly, the substrate MUNANA is cleaved by the enzyme to yield a quantifiable fluorescent product, measured during the assay. The fluorescence was assessed using an excitation wavelength of 365 nm and an emission wavelength of 450 nm and substrate blanks were subtracted from the sample readings. Stock solutions of influenza viruses were freshly prepared by dissolution in MES buffer (32.5 mM 2-(*N*-morpholino)ethanesulfonic acid, pH 6.5) at a concentration of 10 U/mL. Oseltamivir free acid, MS-257 and all of the new compounds described herein were dissolved in water at 20 mM initial concentration. Dilutions of compounds were performed in MES buffer (32.5 mM 2-(*N*-morpholino)ethanesulfonic acid, 4 mM CaCl_2_, pH 6.5), ranging from 2 mM to 5 pM and 6–10 serial dilutions were used; the concentrations depended on the appropriate range for IC_50_ determination, assessed for each compound (inhibitor). Compound 4a[11] was dissolved in DMSO at 20 mM initial concentration. Dilution of inhibitor 4a was performed in MES buffer, ranging from 1 μM to 1 mM, not to exceed 5% DMSO in the final experiment solution. For the inhibition assay, 100 μL of inhibitor solution (in MES buffer, 4 mM CaCl_2_, pH 6.5),) and 5 μL of neuraminidase stock solution (10 U/mL) were preincubated for 45 min at 37°C. The reaction was initiated by the addition of the substrate MUNANA (8 μL of 2.5 mM solution in MES buffer, 4 mM CaCl_2_, pH 6.5) and fluorescence was measured for 5–15 min after addition of the substrate. Graphs of log_10_ concentration of inhibitor versus percent enzyme inhibition compared to the control were plotted to calculate IC_50_. The IC_50_ is the ligand concentration leading to 50% inhibition of the rate of the reaction, compared to the control rate with no inhibitor, calculated using a nonlinear regression function in GraphPad Prism. Inhibition constant *K*_i_ values were calculated using the formula *K*_i_ = IC_50_ / (1 + ([S]/Km)). The mean ± SD of *K*_m_ and *K*_i_ values were obtained from two independent experiments in which the values were calculated by fitting to dose-response curves.

### Virus inhibition assay

Influenza A virus strains A/Puerto Rico/8/32 (PR8, H1N1) and A/Hong Kong/1/68 (HK1, H3N2), both of which are mouse-adapted, were obtained from Dr. E. Brown (University of Ottawa). The replication inhibition assay was performed according to Reference.[31] Briefly, MDCK cells were infected with either PR8 or HK1 strain at 50 tissue culture infectious dose 50 (TCID50) in 96 wells with DMEM containing 0.0075% of trypsin (DMEM−trypsin) in the presence or absence of test compounds. After 2 to 3 days of incubation virus replication was visually determined either by cytopathic effects under light microscope or by immunofluorescent assay with chicken antiserum specific to PR8 strain (Charles River). The compounds were initially dissolved in DMSO and then diluted at least 100 times in DMEM−trypsin. The test was performed in quadruplicate. The inhibition effect was regarded positive when the virus spread was not observed.

## Supporting information

S1 File Crystal structure of compound 15c. (PDF)

S2 File Crystallographic information of compound 15c. (CIF)

S3 File ^1^H- and ^13^C- NMR spectra of compounds. (PDF)
