# Supplementary material for: Design and synthesis of constrained bicyclic molecules as candidate inhibitors of influenza A neuraminidase
Source: PLoS One. 2018 Feb 28;13(2):e0193623. doi: 10.1371/journal.pone.0193623 (PMC5831633; doi:10.1371/journal.pone.0193623)
Supplement: S1 File — (PDF) [file pone.0193623.s001.pdf]

S1 File. Crystal structure of compound 15c.

Design and synthesis of constrained bicyclic molecules as candidate inhibitors of influenza A neuraminidase

Cinzia Colombo[1,2], Črtomir Podlipnik[3], Leonardo Lo Presti[1], Masahiro Niikura[4], Andrew J. Bennet[2], Anna Bernardi[1]*

1 Università degli Studi di Milano, Dipartimento di Chimica, Milano, Italy

2 Department of Chemistry, Simon Fraser University, Burnaby, British Columbia, Canada

3 University of Ljubljana, Faculty of Chemistry and Chemical Technology, Ljubljana, Slovenia

4 Faculty of Health Sciences, Simon Fraser University, Burnaby, British Columbia, Canada

*Address correspondence to Cinzia Colombo, Università degli Studi di Milano, Dipartimento di Chimica, Via Golgi 19, I-20133 Milano, Italy. E-mail: cinzia.colombo@unimi.it;

Table of contents

Table A	Geometric parameters for compound **15c**
Figure A	Crystal Structure**:** Hydrogen-bonded pillars formed by **15c** in the solid state
Figure B	Crystal packing of **15c** as see along the b cell axis
Appendix A	Crystal Structure Comments

Table A.

Geometric parameters for symmetry-independent relevant hydrogen-bonded contacts N-H···O with $d_{H\cdots A}$ ≤ 3.0 Å and α_{DHA} ≥ 120 deg in compound **15c** at room temperature. Values in Å e deg, with sensible estimated standard deviations reported in parentheses.

D–H···A	D-H	H···A	D···A	D-H···A	Symmetry operation
N1–H1···O5	0.860	2.251	3.080(3)	162	x, −1+y, z
N2–H2···O1	0.860	2.141	2.952(3)	157	x, +1+y, z

Figure A. Crystal Structure: Hydrogen-bonded pillars formed by 15c in the solid state

Hydrogen-bonded pillars formed by **15c** in the solid state. HB contacts shown in Table S1 are highlighted as red dotted lines.

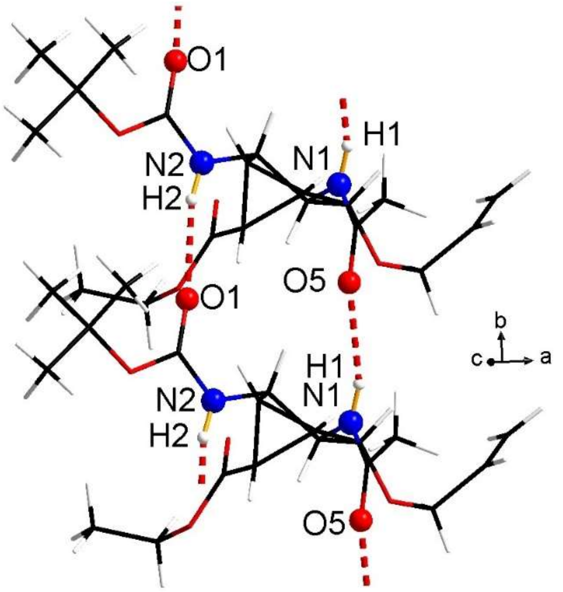

Figure B. Crystal packing of 15c as see along the b cell axis

Crystal packing of **15c** as see along the b cell axis, with the symmetry-related voids highlighted as green circles.

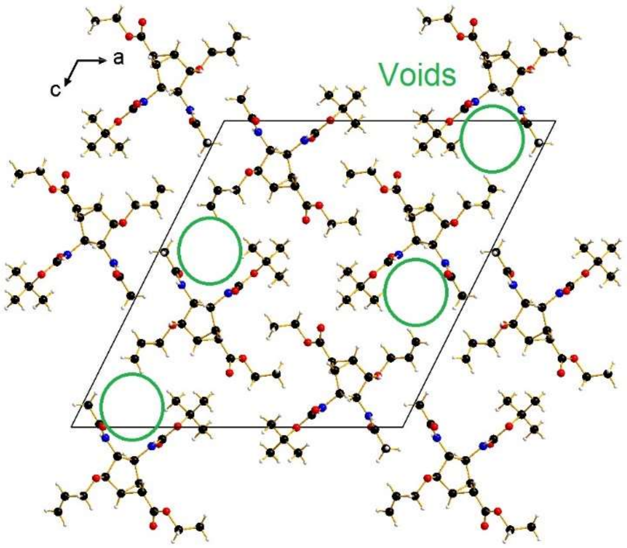

Appendix A. Crystal Structure Comments

Two significant hydrogen bond (HB) donors are present in **15c**, namely the amide N1–H1 and N2–H2 groups. These functions are employed to set up allegedly strong H-bonded pillars along the monoclinic *b* axis, which involve the carbamate and the acetamide carbonyl groups (Figure 6 in the main text and Figure S2). Cumbersome substituents, on the other hand, are allocated in the free space between adjacent pillars and extend mainly in the (*a*,*c*) plane. This arrangement is likely related to the observed anisotropy of the lattice constants, *i.e.* to the fact that $a \approx c >> b$. The large apparent displacement detected for the terminal C13 methyl group in the t-butyl moiety (Figure 6 in the main text) might imply some kind of either static or dynamic disorder affecting this group; measurements as function of temperature are required to clarify the issue.

Four symmetry-related voids were found in the unit cell, each ≈ 31 $Å^3$ large (Figure S3, symmetry-independent crystallographic coordinates: 0.126, -0.141, 0.893). They are likely too small to accommodate disordered solvent molecules (to the sake of comparison, the molecular volume of water is ≈ 40 $Å^3$).

Actually, the SQUEEZE procedure implemented in Platon (Spek, A. L., Acta Cryst. 2015, C71, 9-18) does not imply a significant gain in the model quality. Moreover, the Fourier residual maps in the void regions are essentially featureless, with maximum (minimum) residual densities being as large as +0.27 e·Å^3 (–0.19 e·Å^3). It can be concluded that the small voids present in this structure are due to the intrinsic mutual arrangement of bulky substituents in the (*a*,*c*) plane (Figure S3 above), while the crystal is stabilized through H-bonded pillars running along the perpendicular *b* direction (Fugure 6 in the main text and S2 above). It is interesting to note that the terminal =CH_2 group is close to voids and this could explain the large thermal ellipsoid associated to the C19 atom (Figure 6 in the main text).
