# Supplementary material for: Design and synthesis of constrained bicyclic molecules as candidate inhibitors of influenza A neuraminidase
Source: PLoS One. 2018 Feb 28;13(2):e0193623. doi: 10.1371/journal.pone.0193623 (PMC5831633; doi:10.1371/journal.pone.0193623)

S3 File. ^{1}H- and ^{13}C- NMR spectra of compounds.

Design and synthesis of constrained bicyclic molecules as candidate inhibitors of influenza A neuraminidase

Cinzia Colombo[1,2], Črtomir Podlipnik[3], Leonardo Lo Presti[1], Masahiro Niikura[4], Andrew J. Bennet[2], Anna Bernardi[1]*

[1] Università degli Studi di Milano, Dipartimento di Chimica, Milano, Italy

[2] Department of Chemistry, Simon Fraser University, Burnaby, British Columbia, Canada

[3] University of Ljubljana, Faculty of Chemistry and Chemical Technology, Ljubljana, Slovenia

[4] Faculty of Health Sciences, Simon Fraser University, Burnaby, British Columbia, Canada

[*]Address correspondence to Cinzia Colombo, Università degli Studi di Milano, Dipartimento di Chimica, Via Golgi 19, I-20133 Milano, Italy. E-mail: cinzia.colombo@unimi.it;

Table of contents

Appendix A	Numbering of the bicyclo[3.1.0]hexanes **15** for spectral assignment	page S2
Figure A	^{1}H NMR of crude reaction mixture of compound **12** with 3-pentanol	page S2
	^{1}H- and ^{13}C- NMR spectra of compounds	page S3-S34

Appendix A. Product numbering

Numbering of the bicyclo[3.1.0]hexanes **15** for spectral assignment.

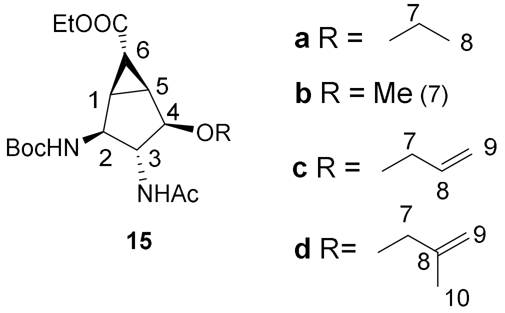

15

Figure A. ^{1}H-NMR of crude reaction mixture of compound 12 with 3-pentanol.

^{1}H NMR of crude reaction mixture for the reaction of compound **12** with 3-pentanol (on top) and products isolated by chromatography.

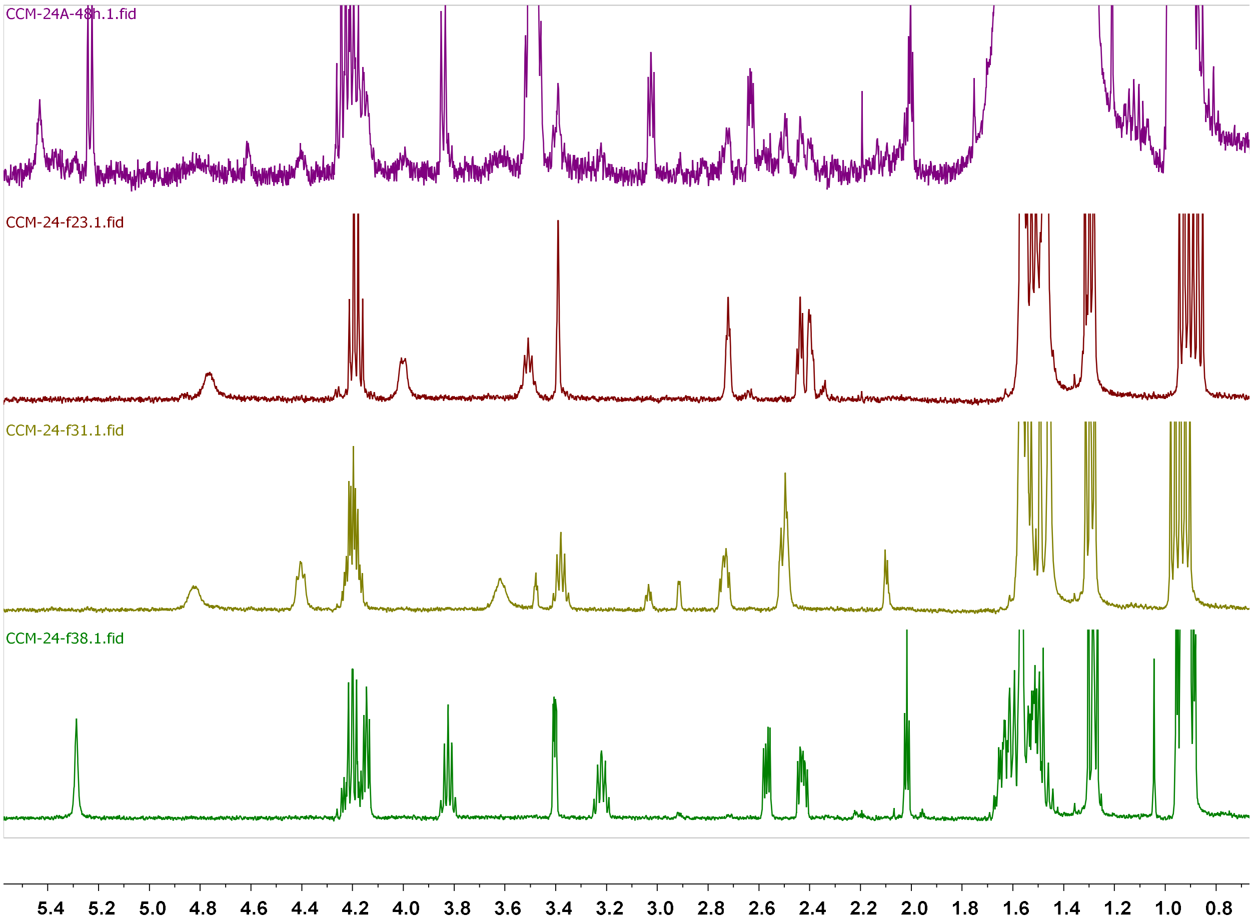

^{1}H- and ^{13}C- NMR spectra of compounds

Compound 12

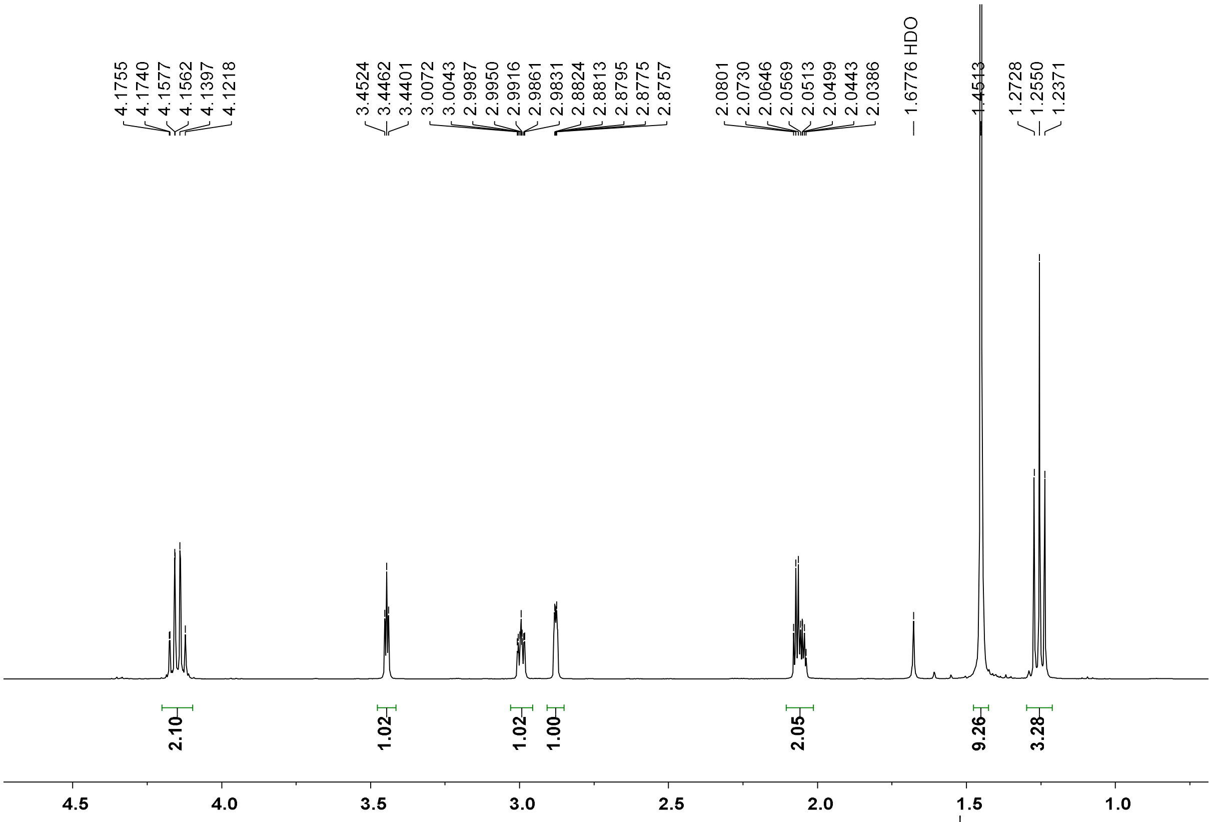

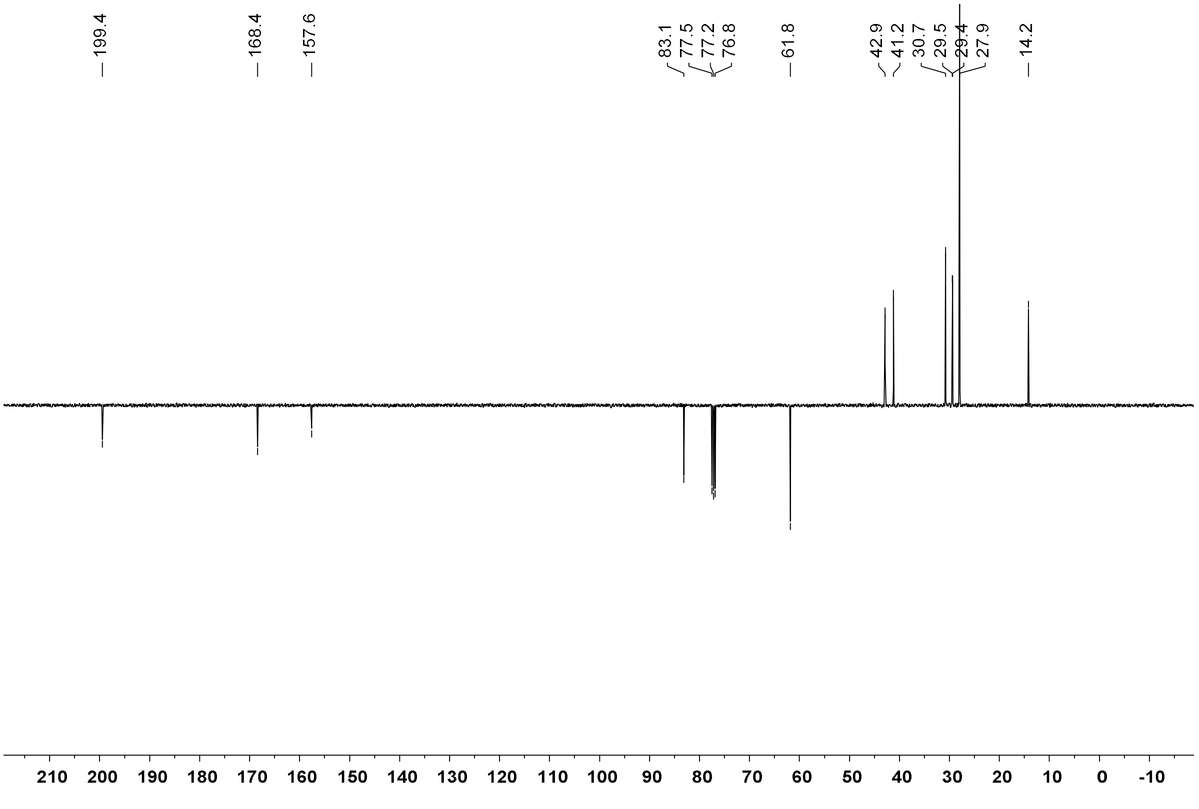

Compound 13a

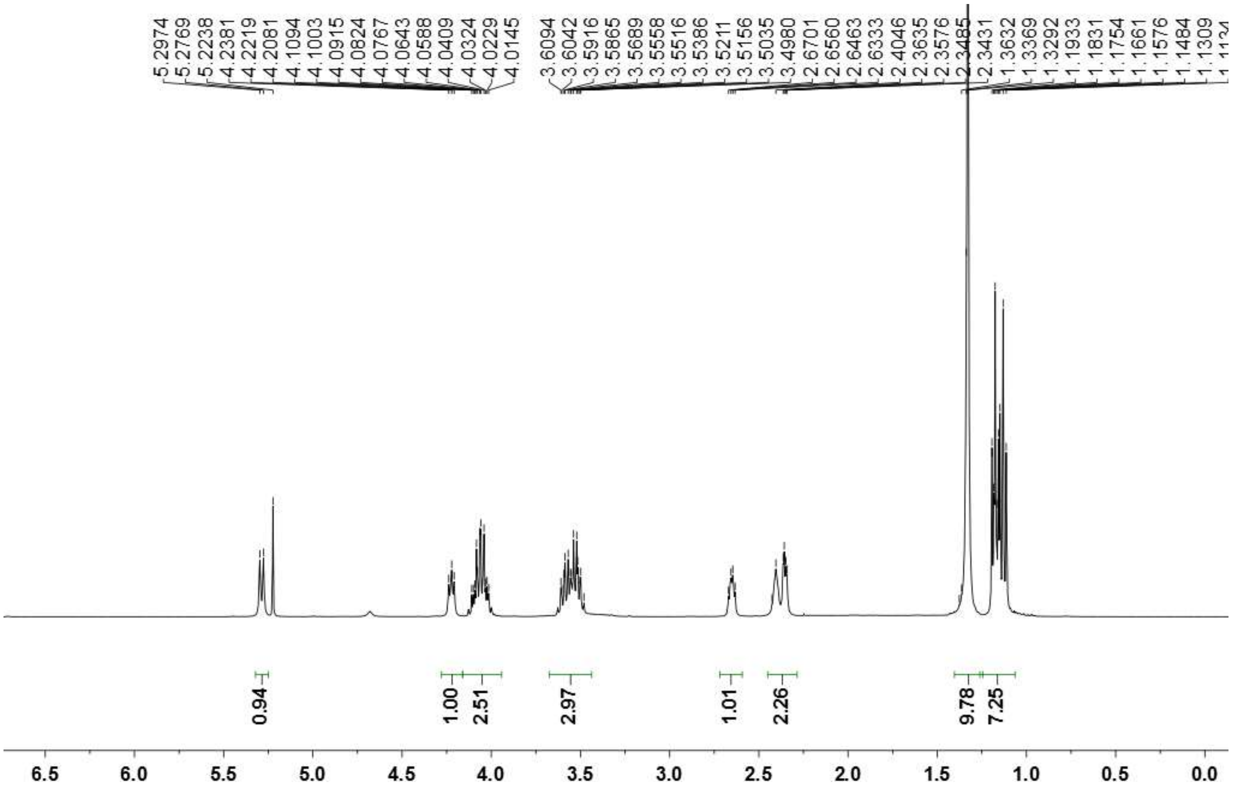

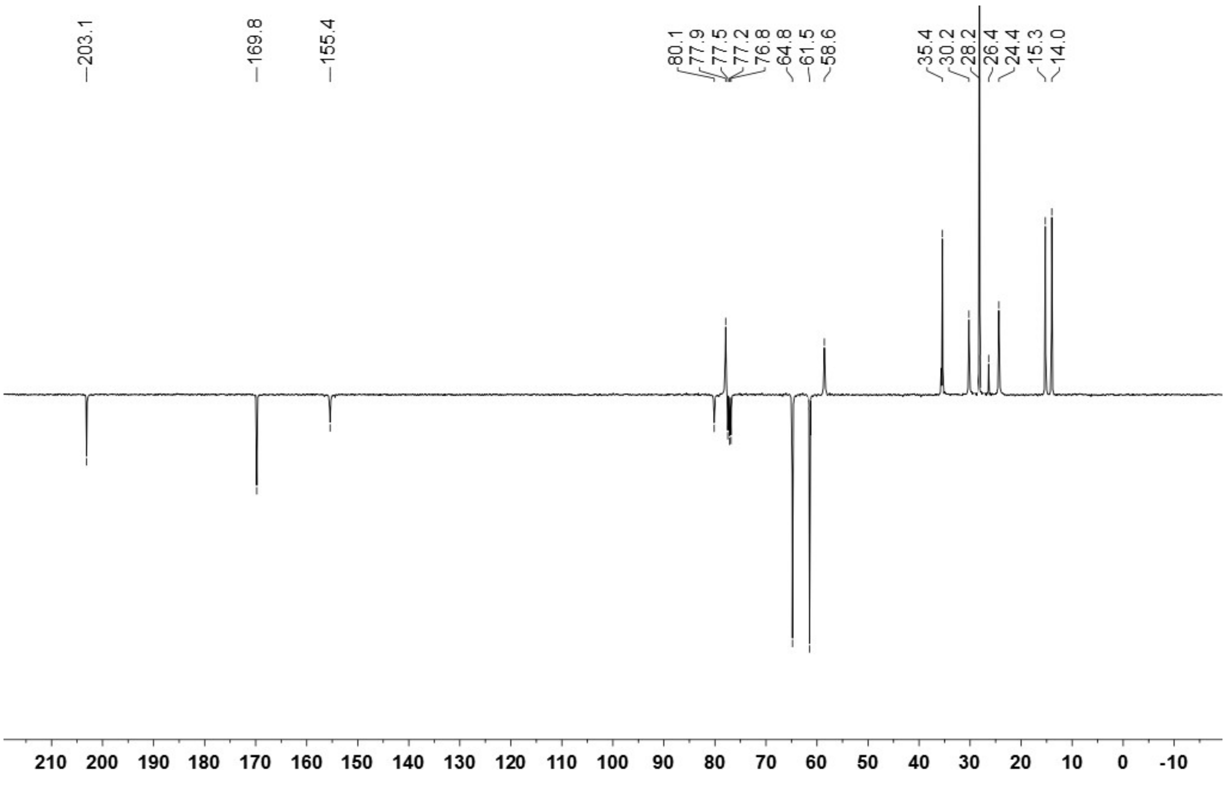

Compound 13b

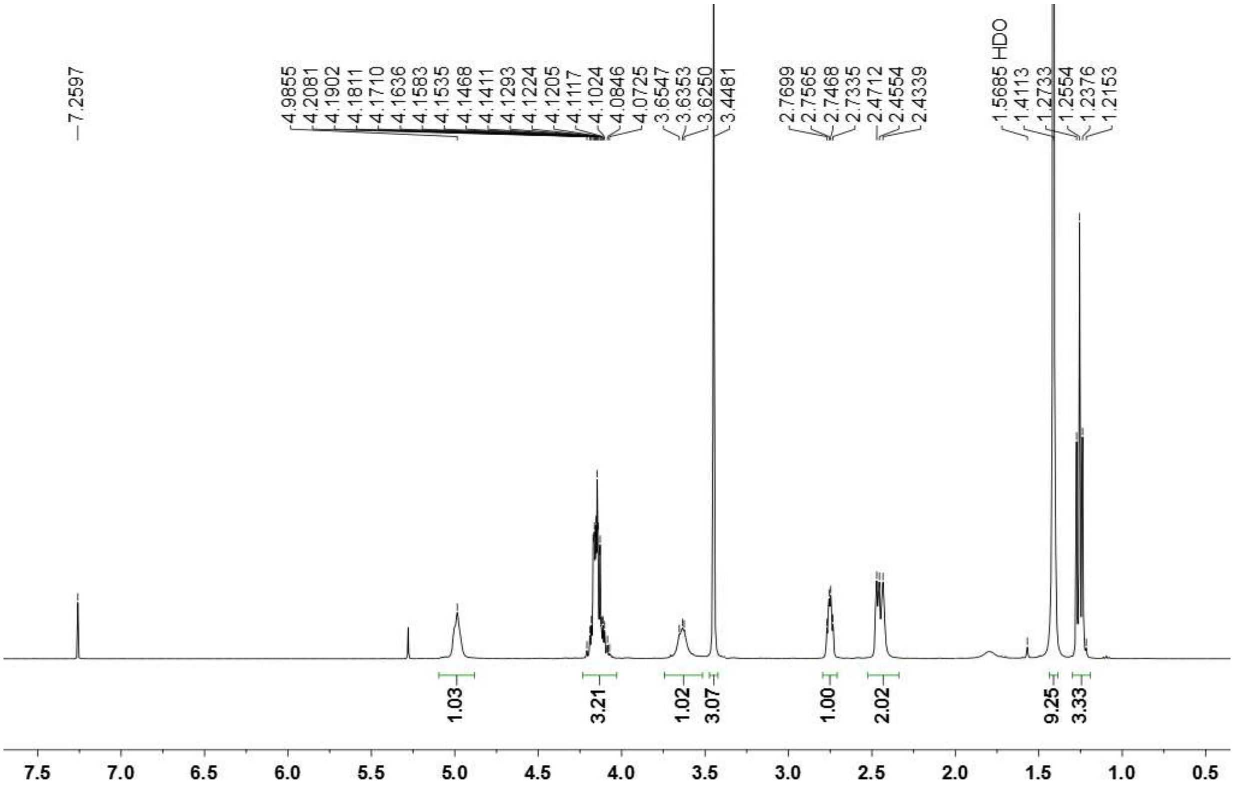

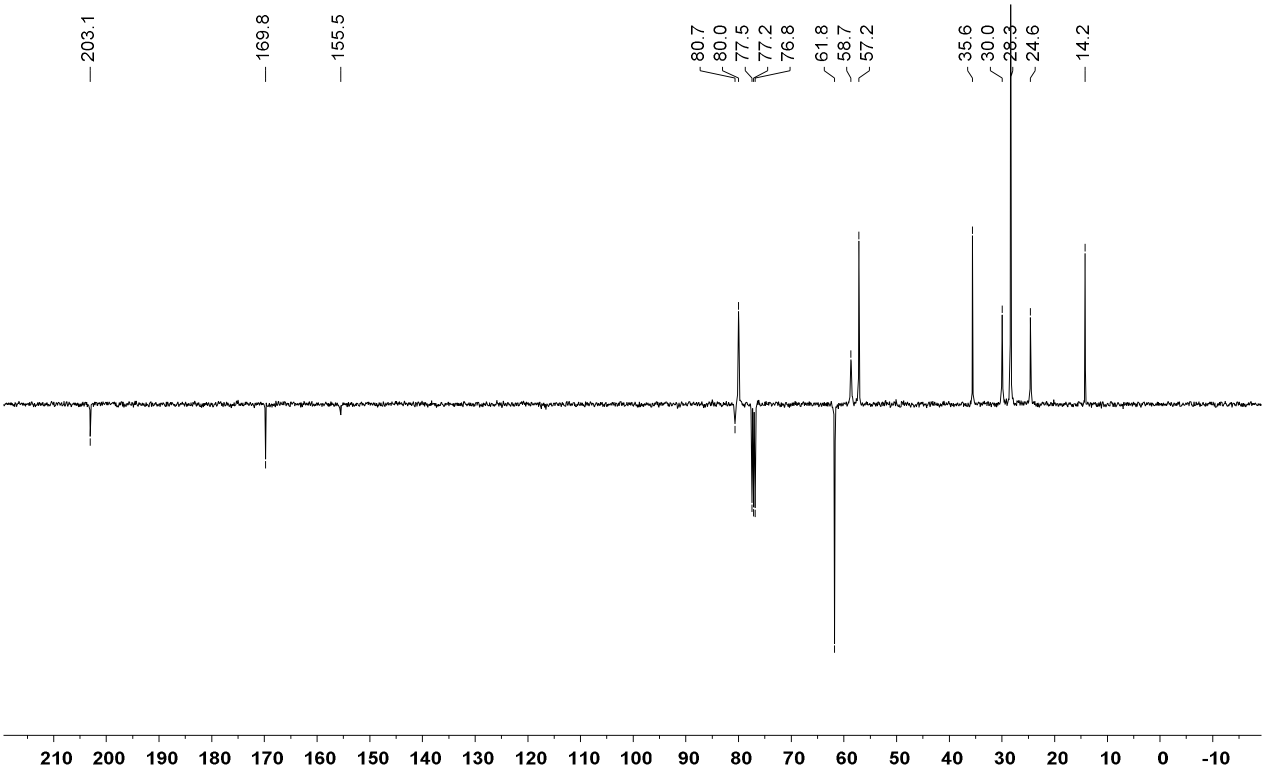

Compound 13c

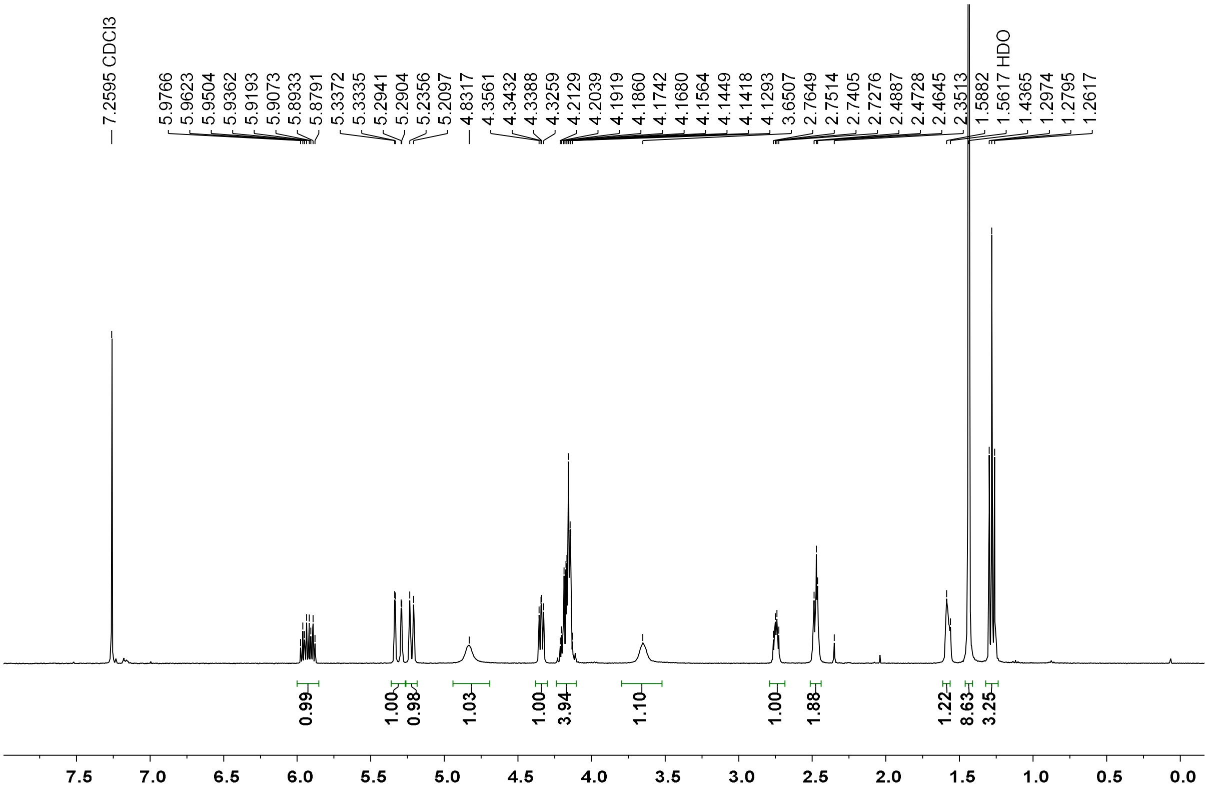

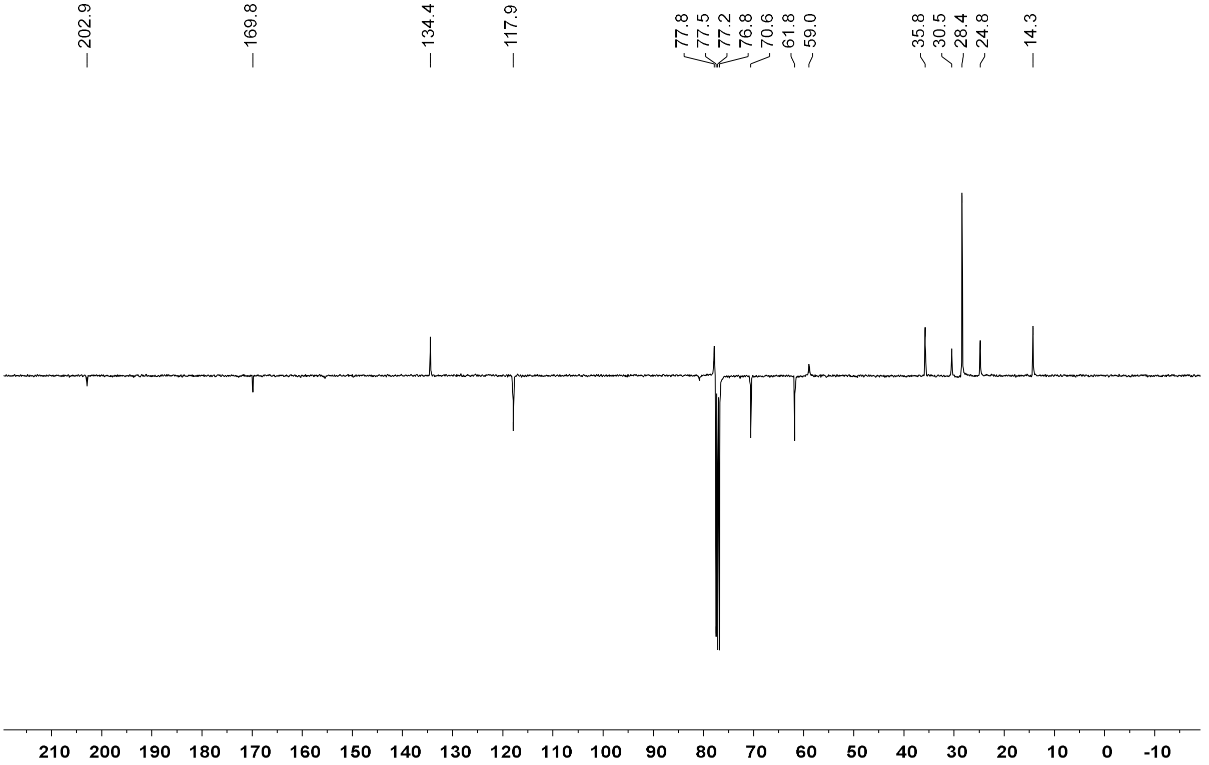

Compound 13d

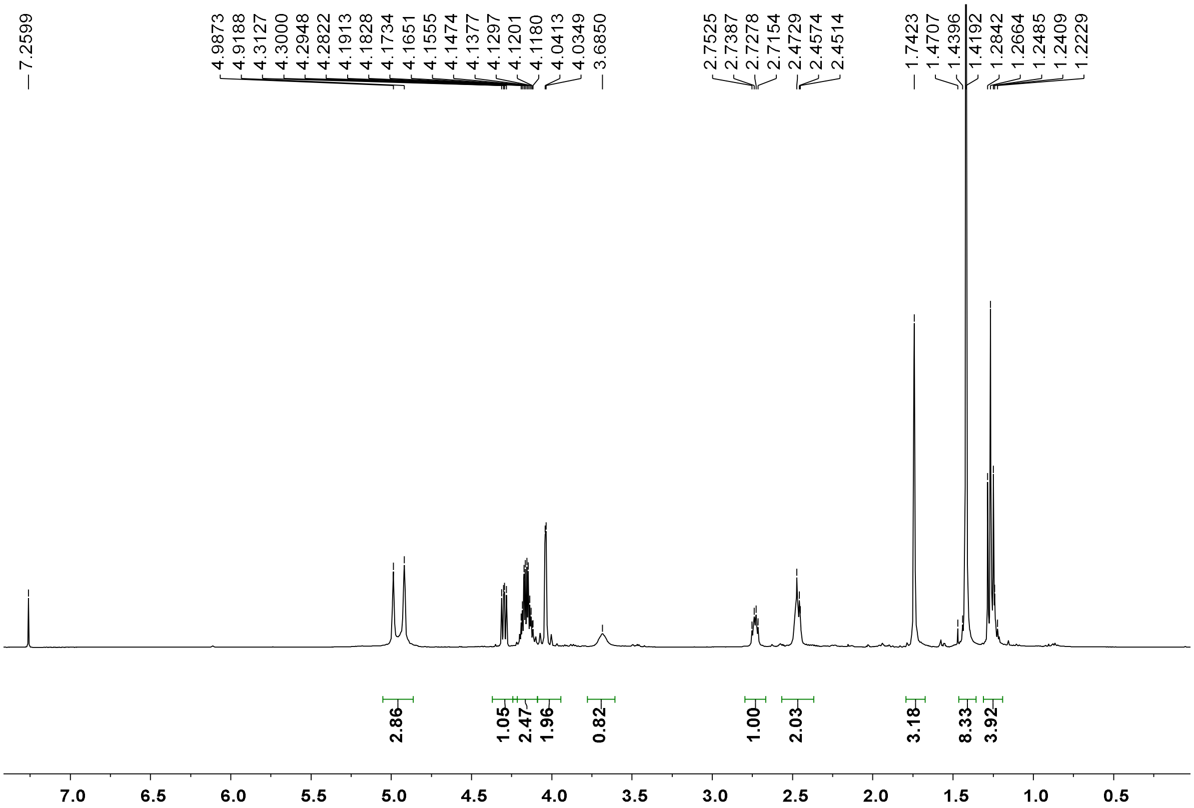

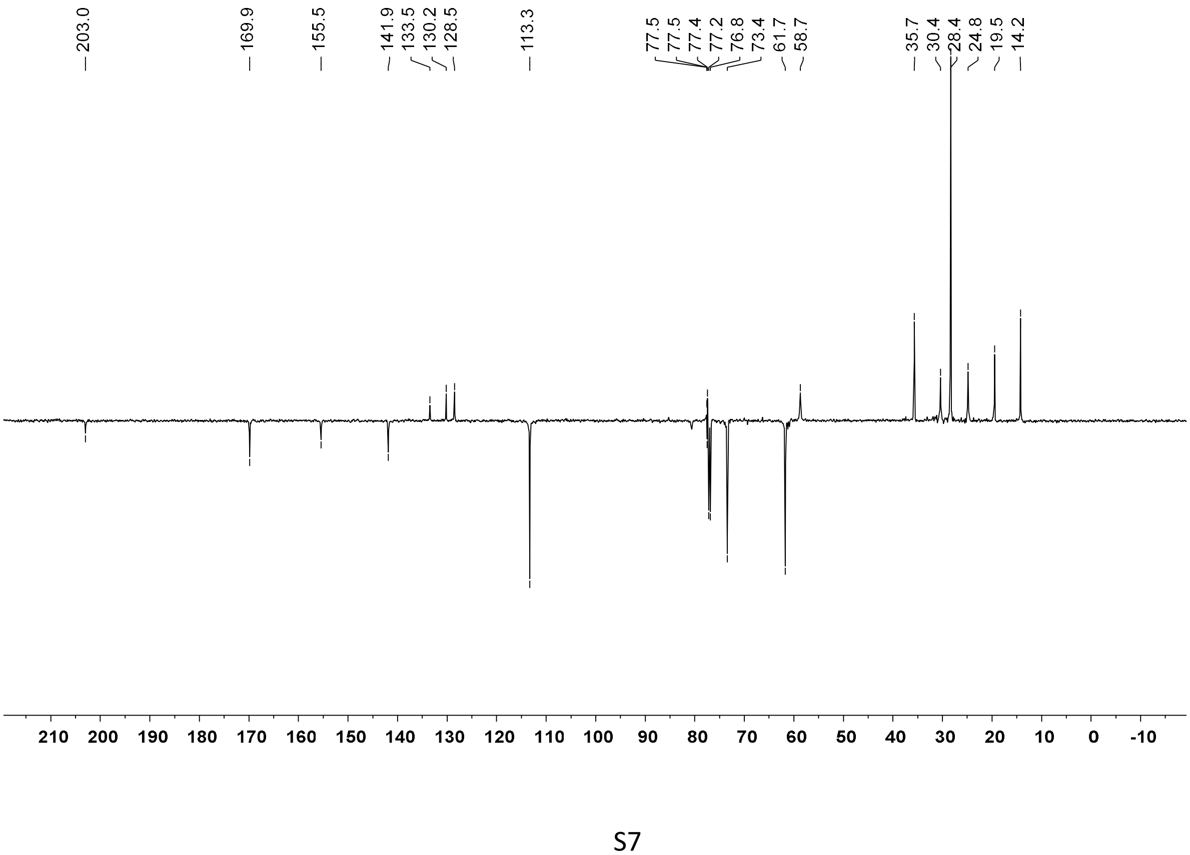

Compound 14a

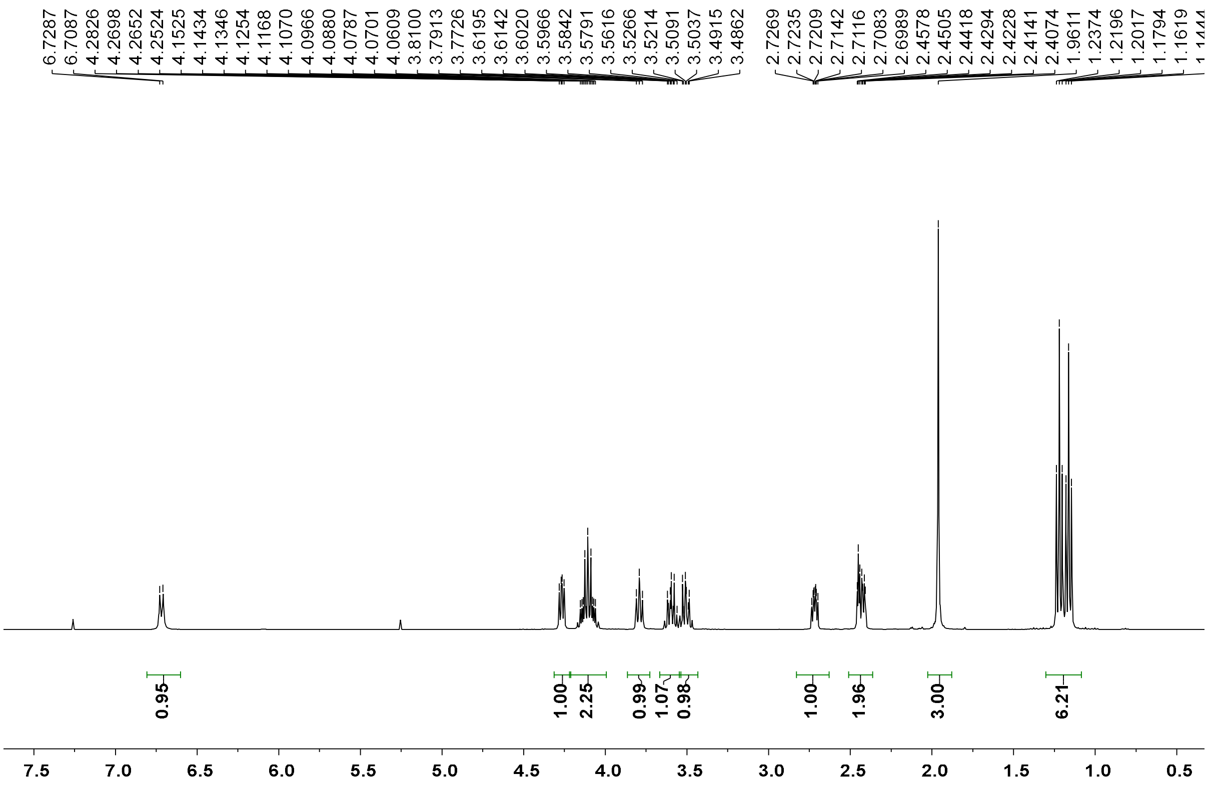

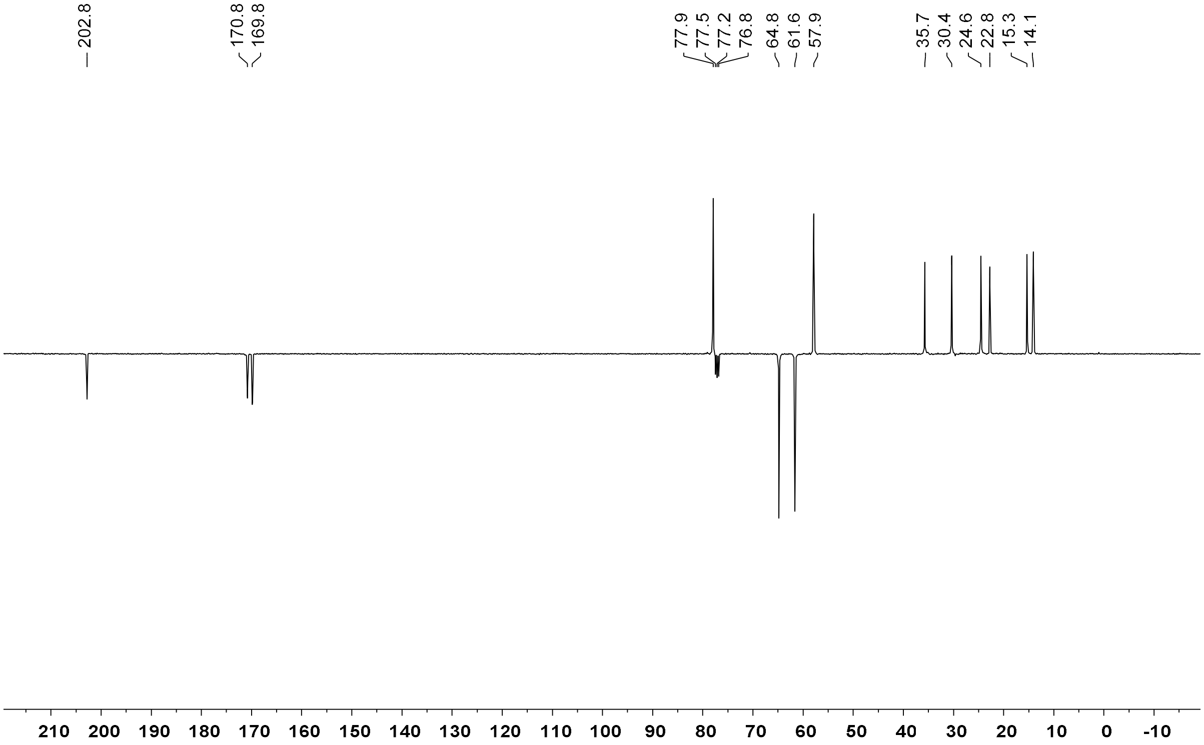

Compound 14b

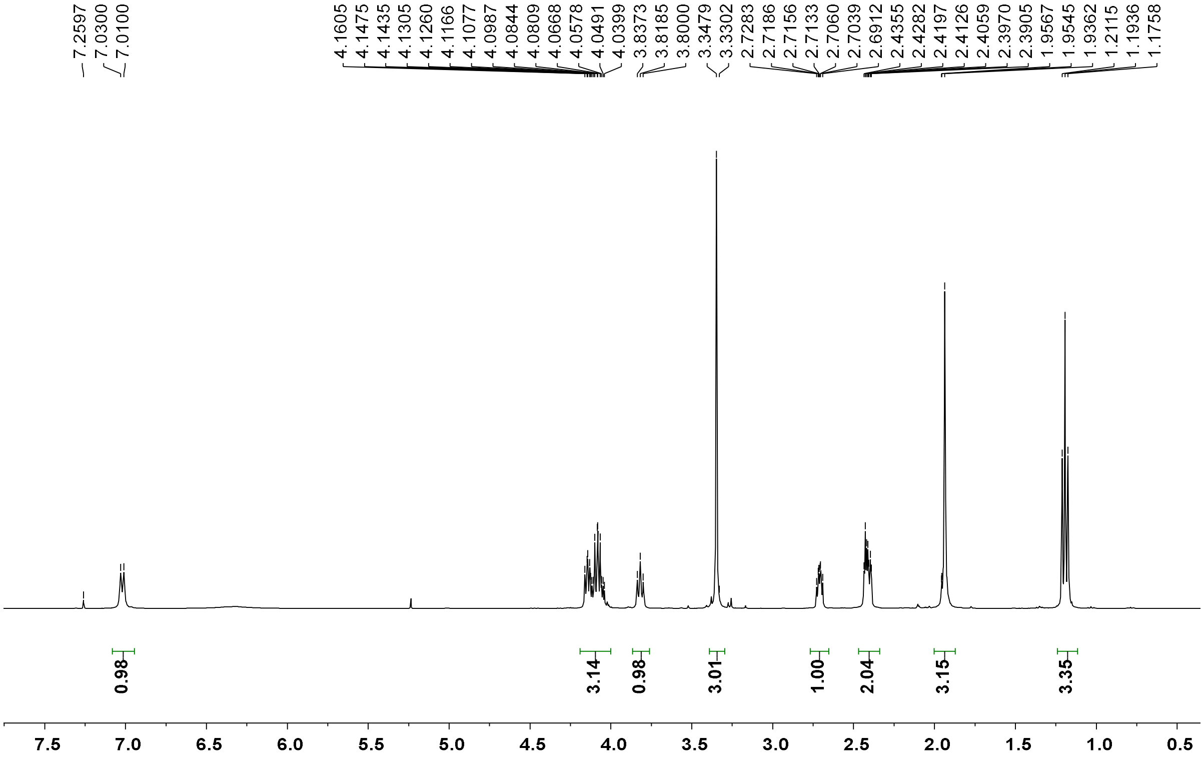

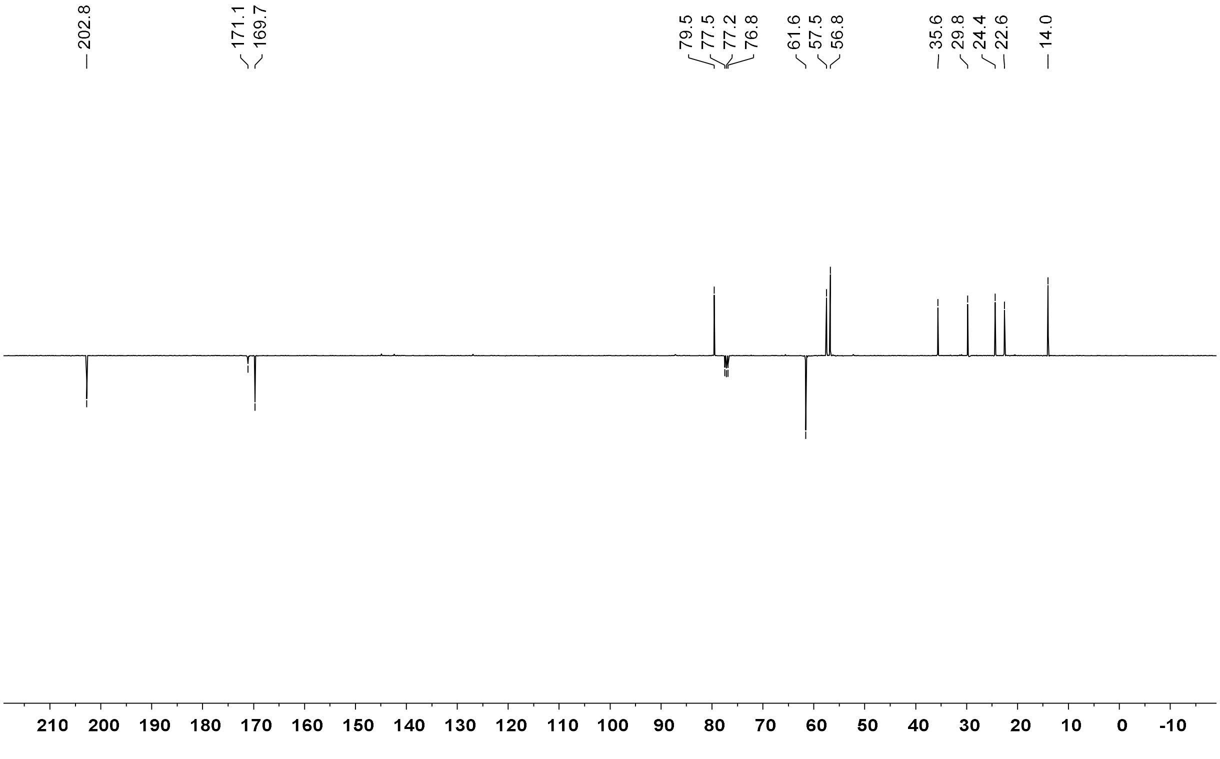

Compound 14c

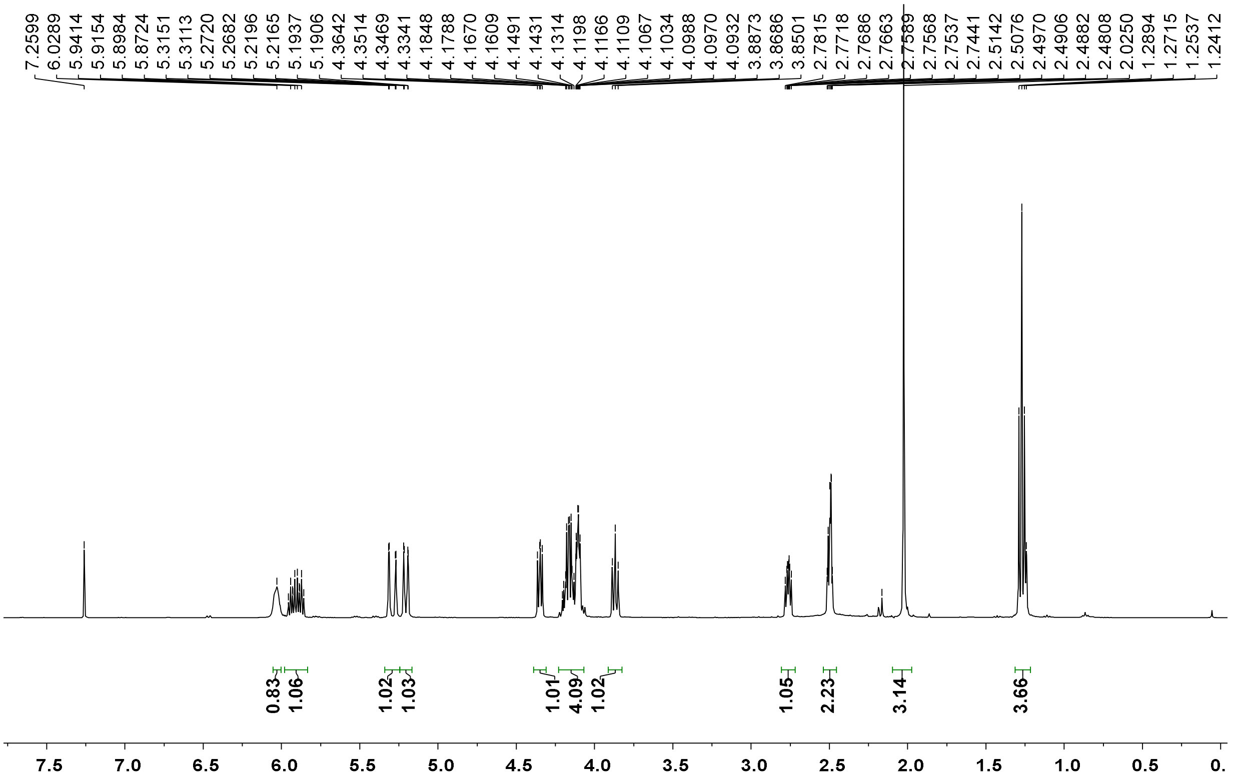

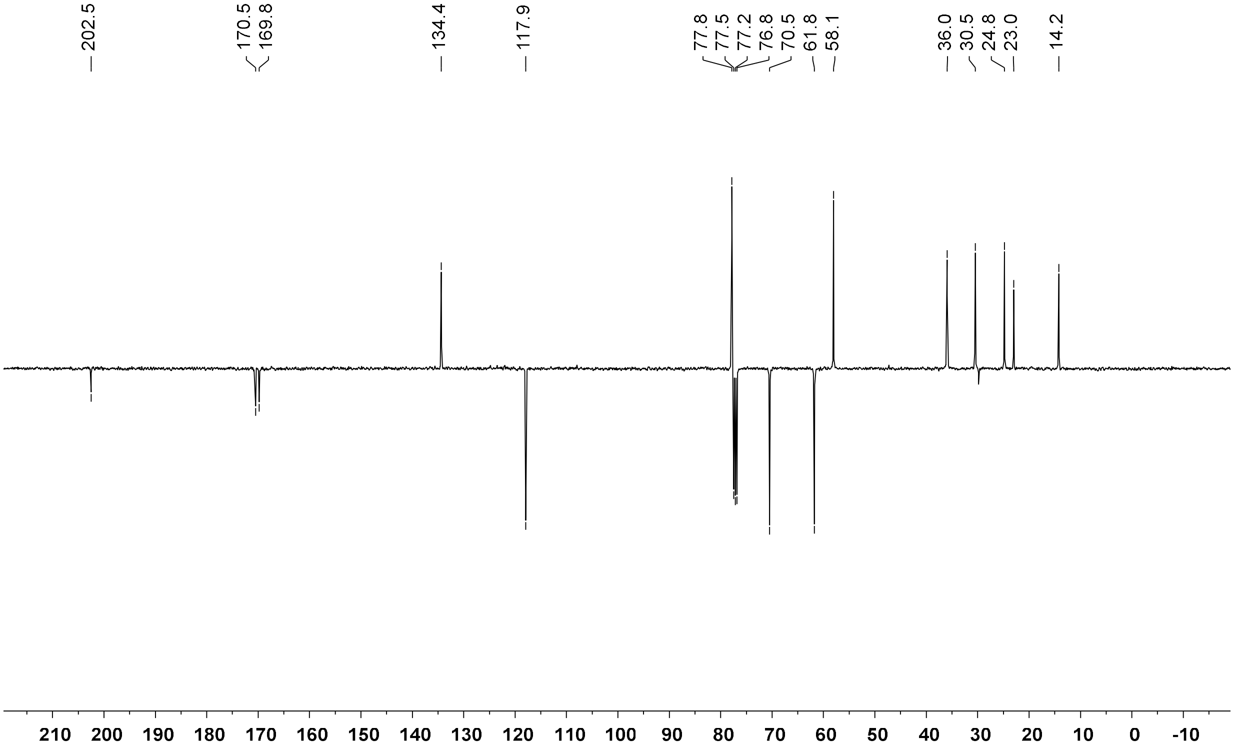

Compound 14d

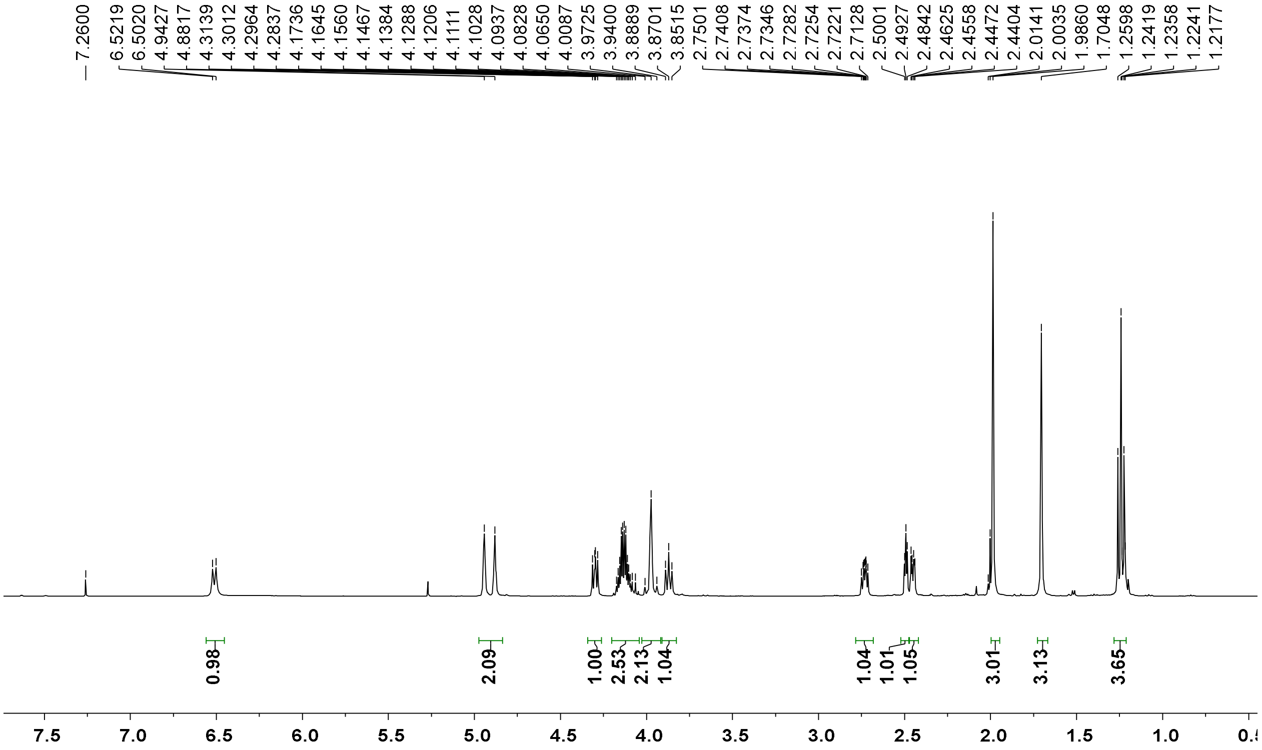

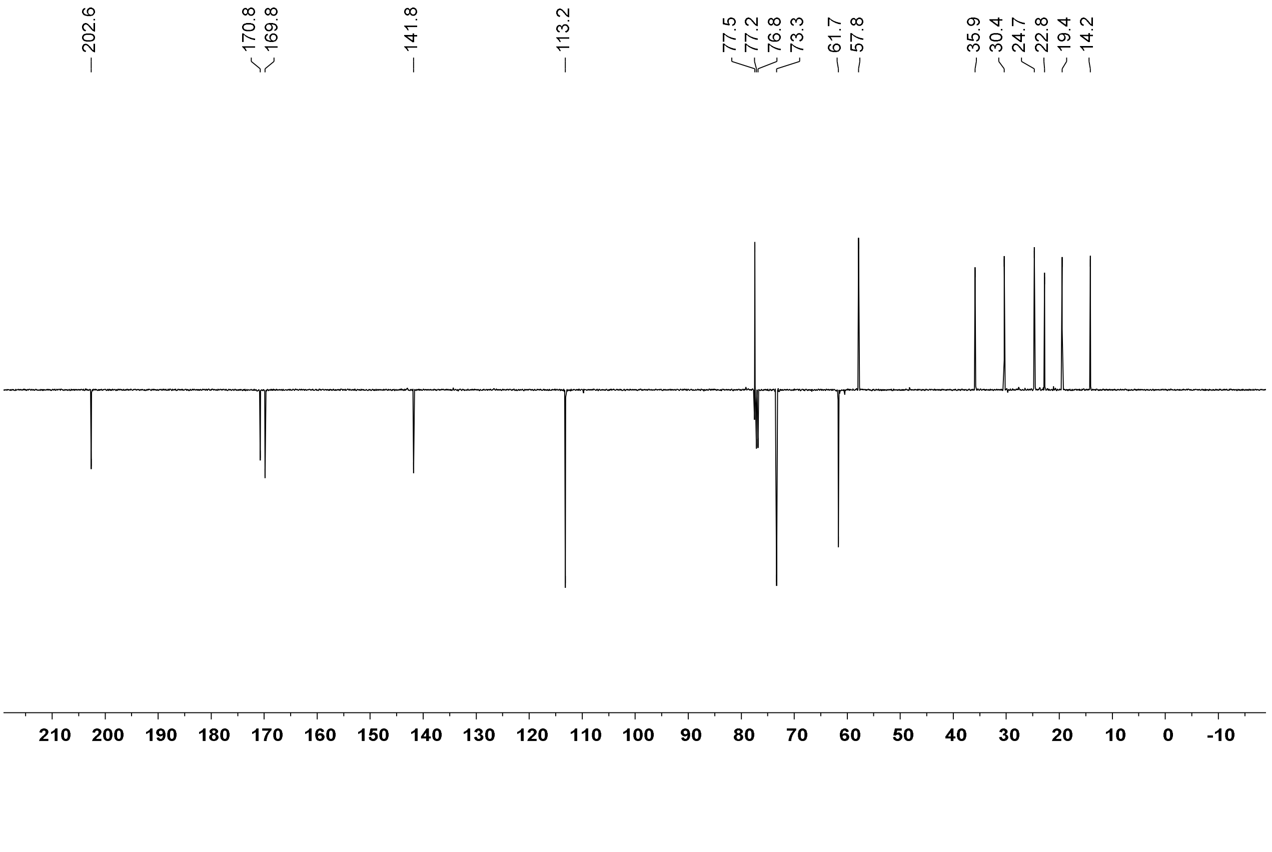

Compound 15a

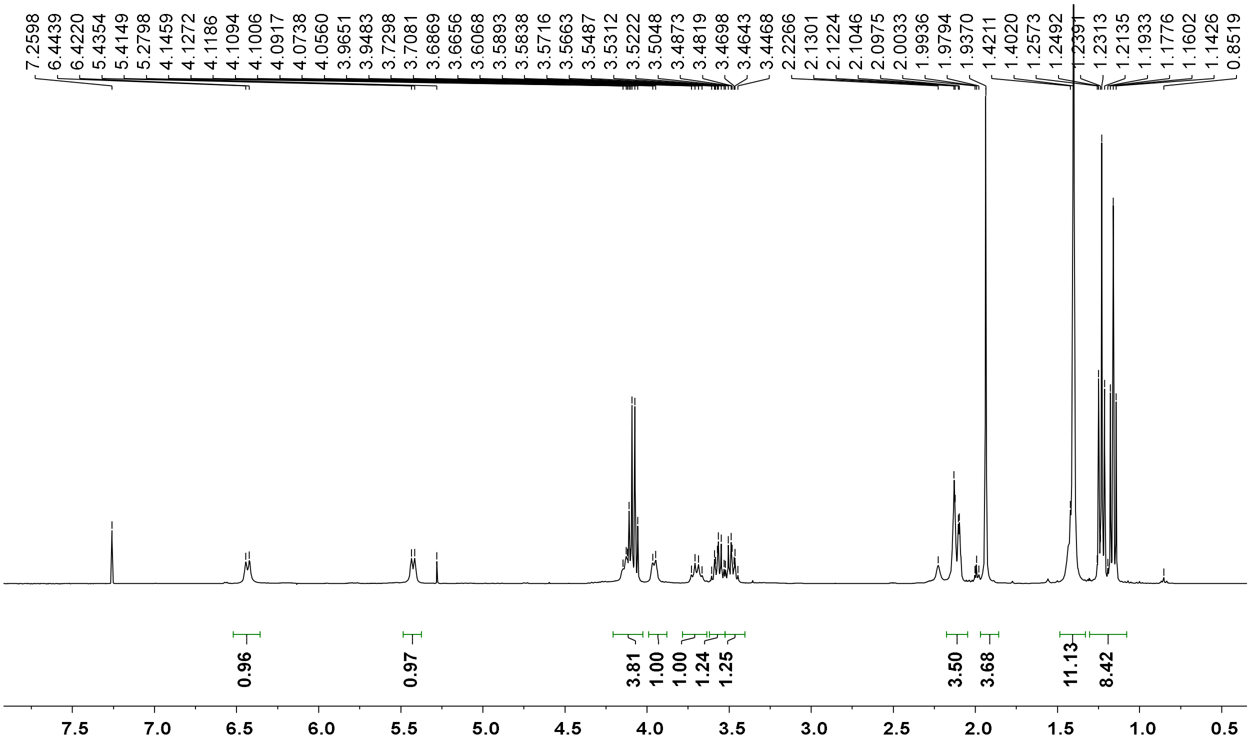

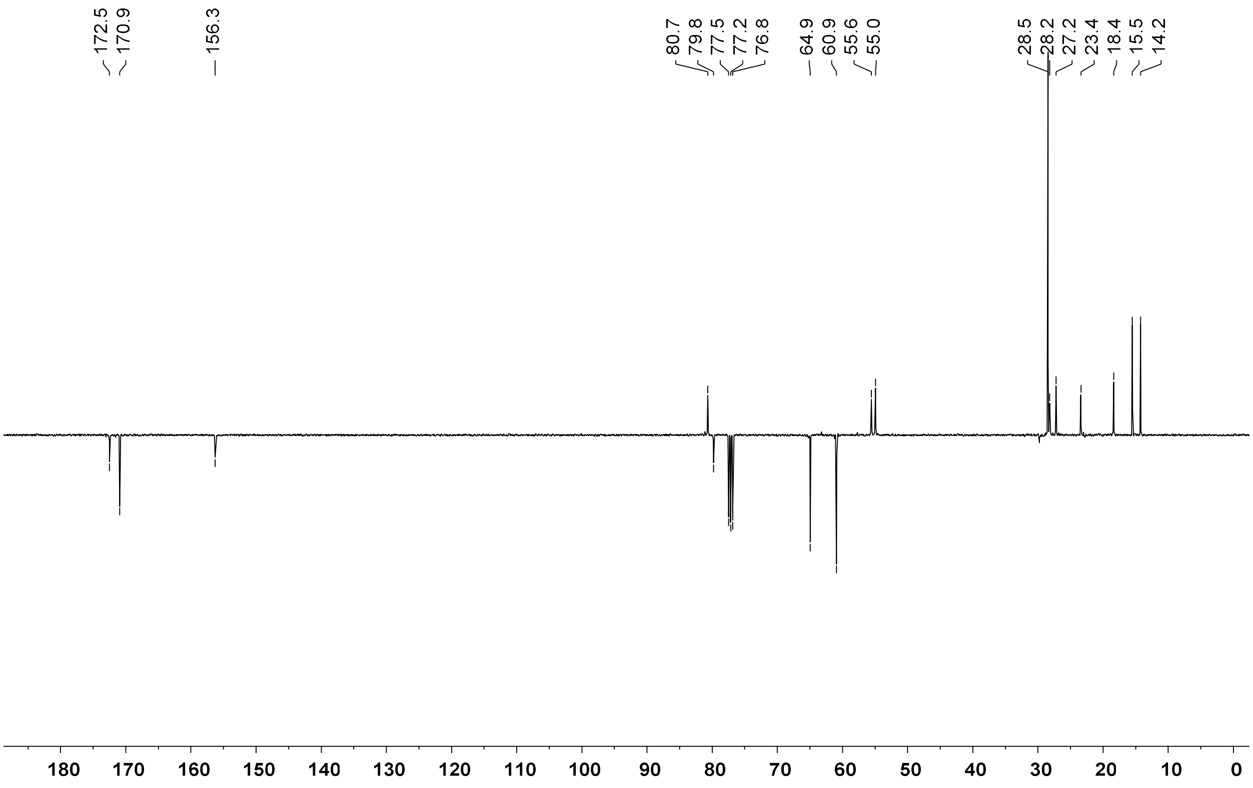

Compound 15b

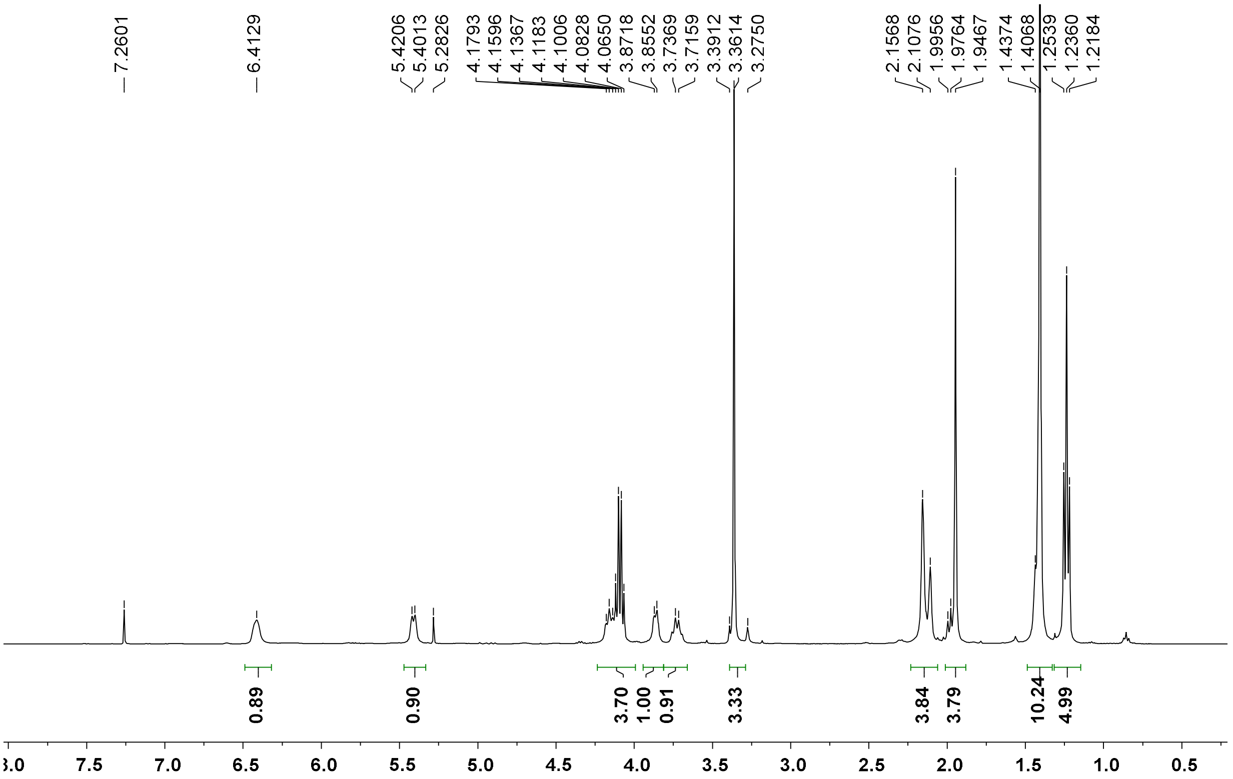

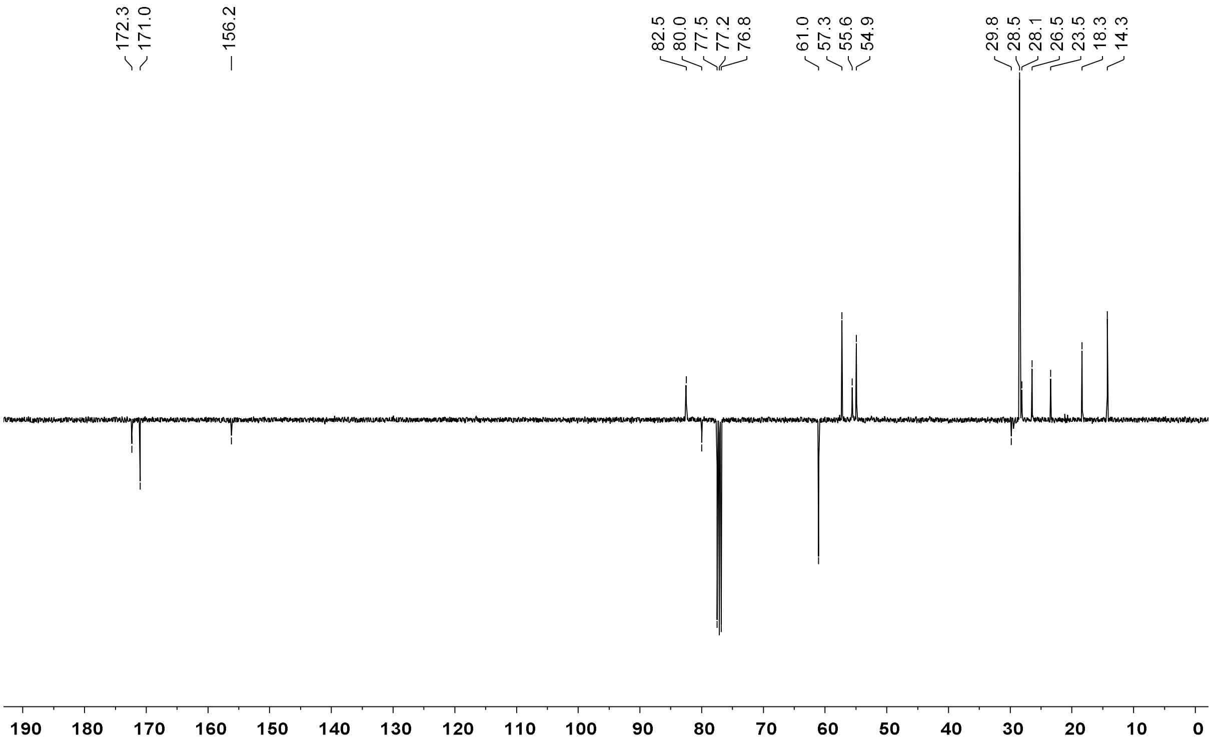

Compound 15c

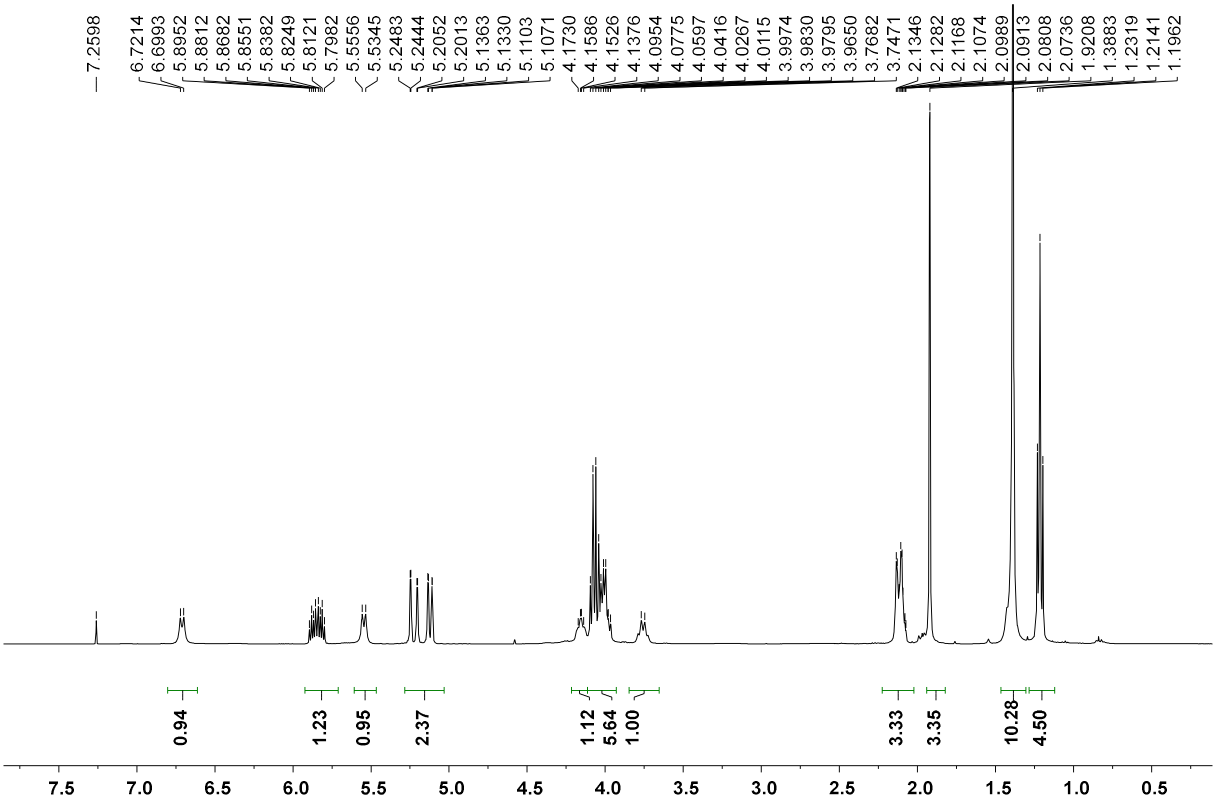

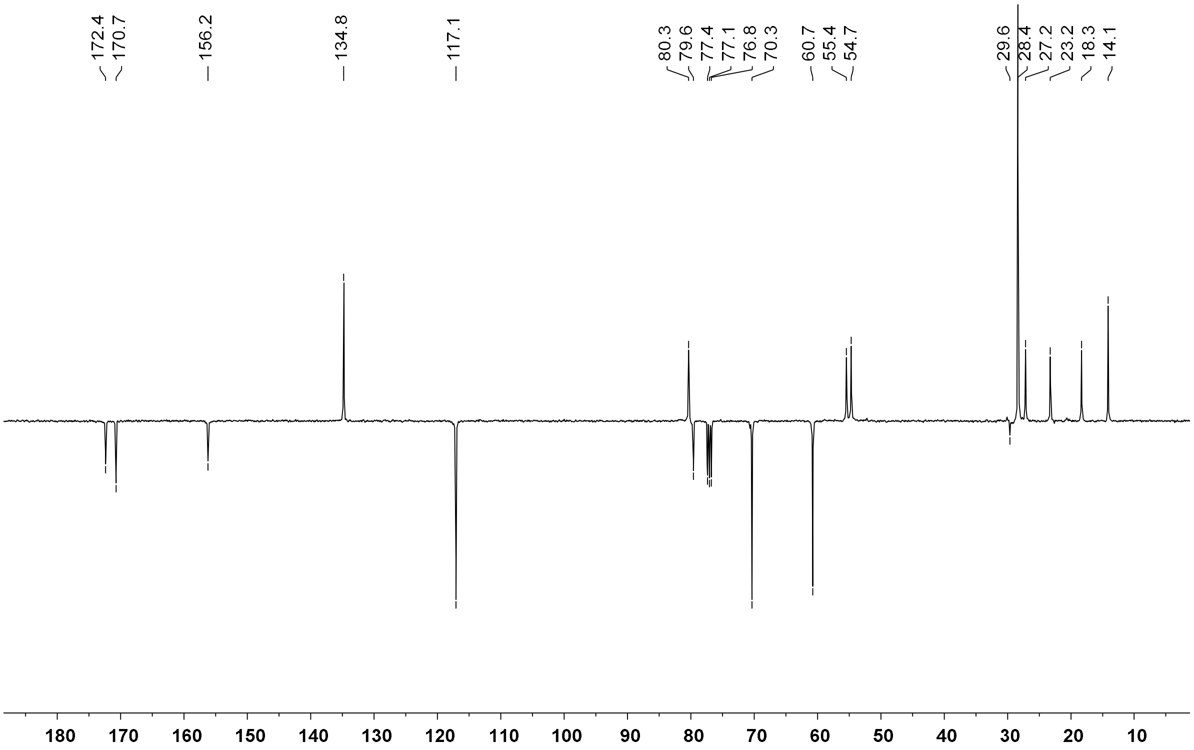

Compound 15d

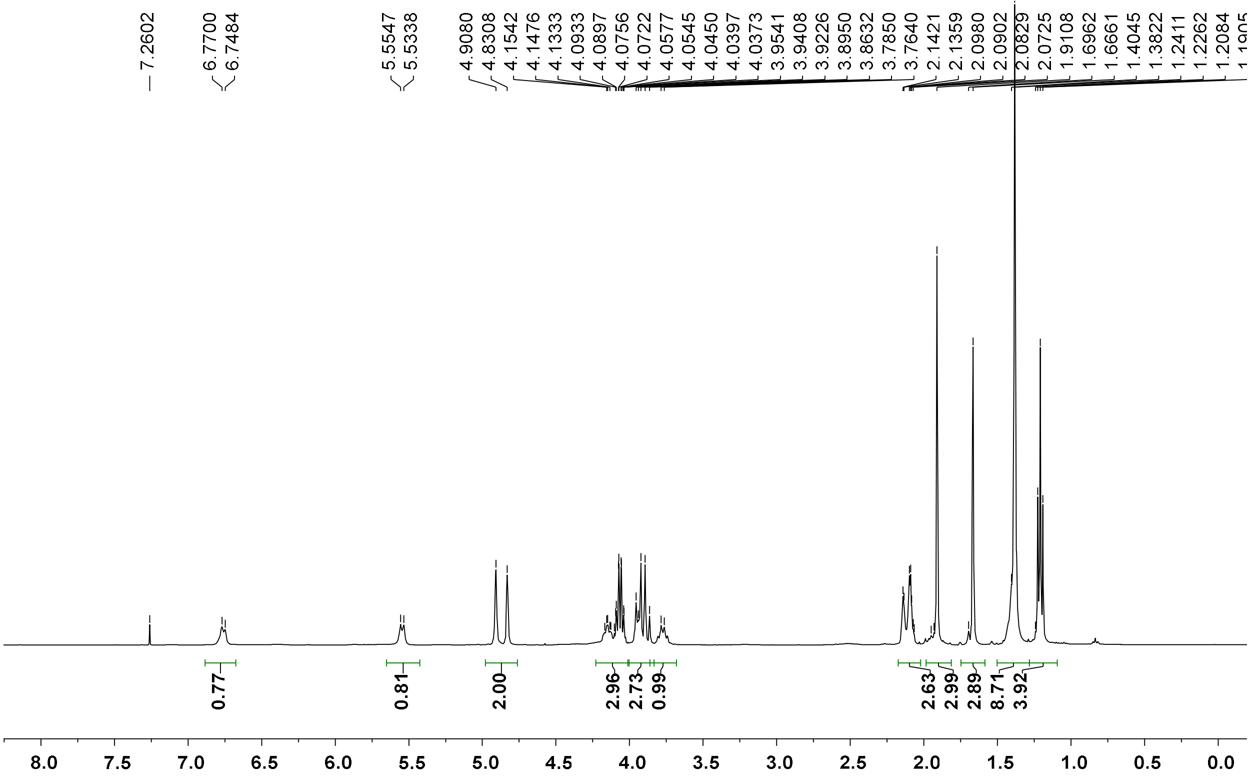

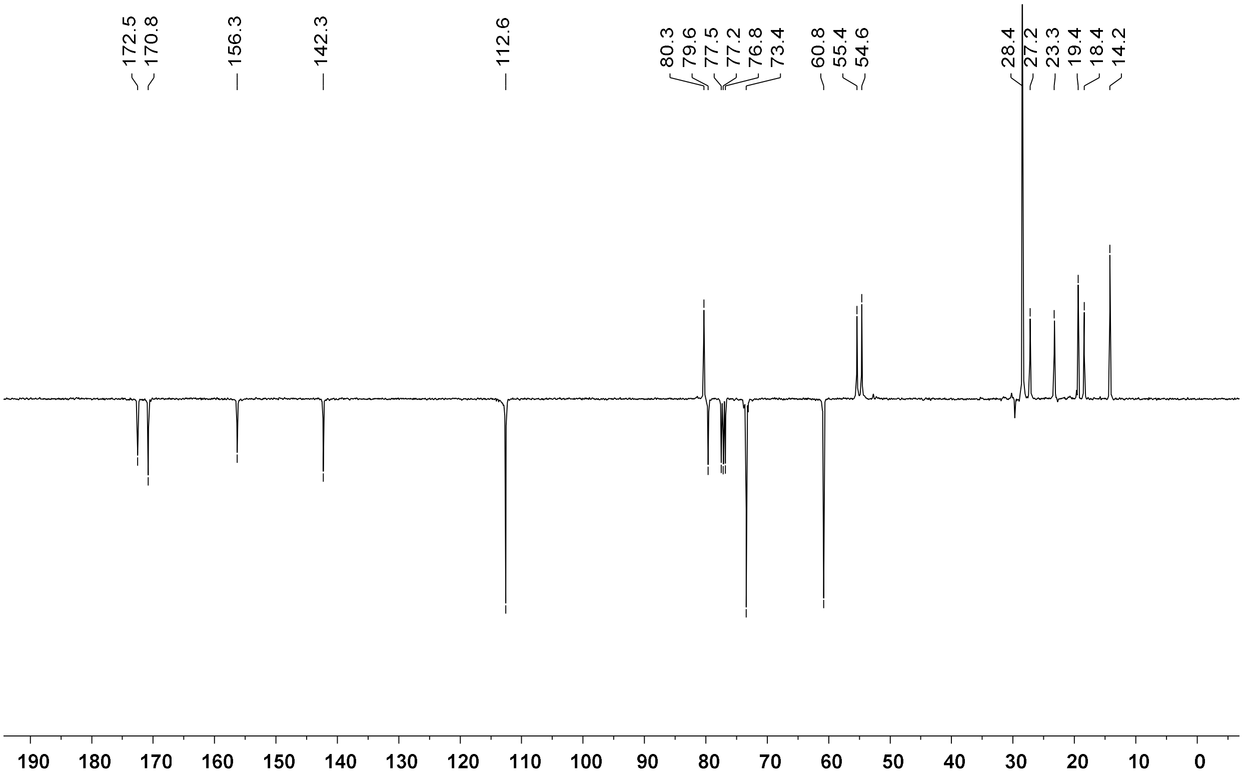

Compound 15e

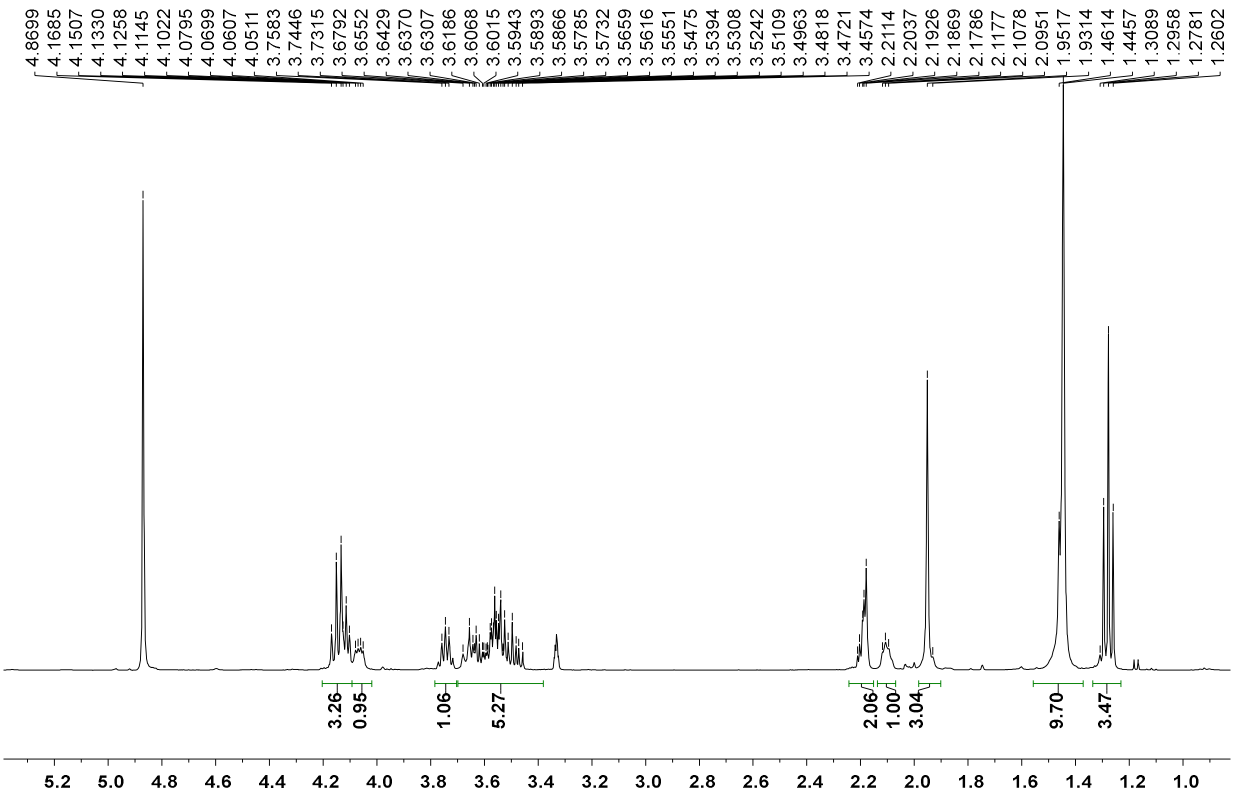

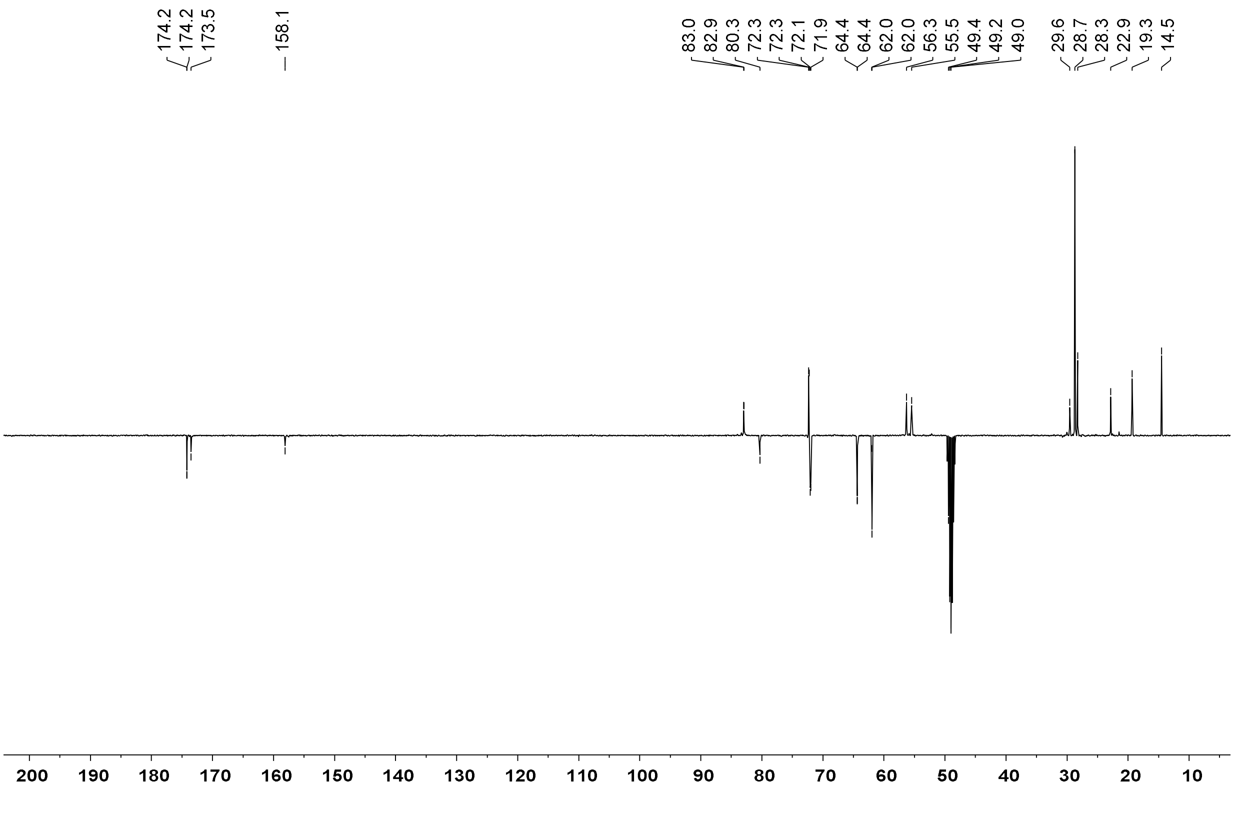

Compound 15f

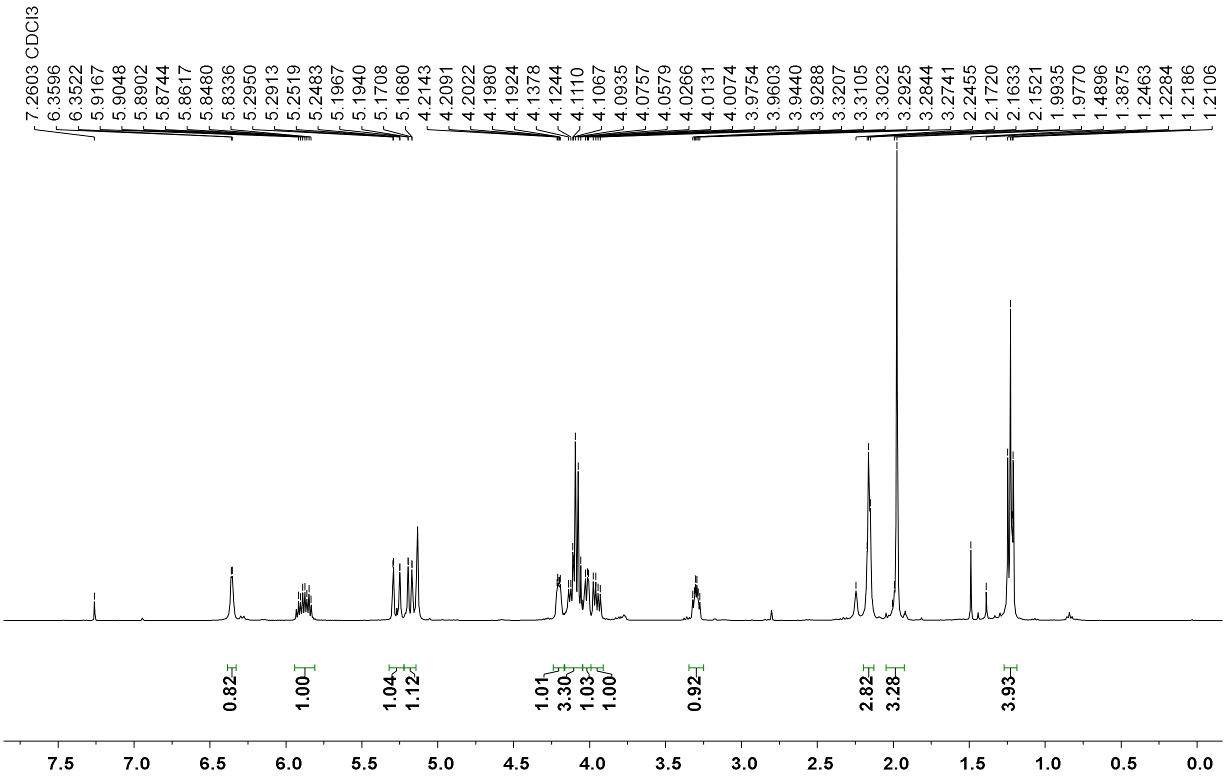

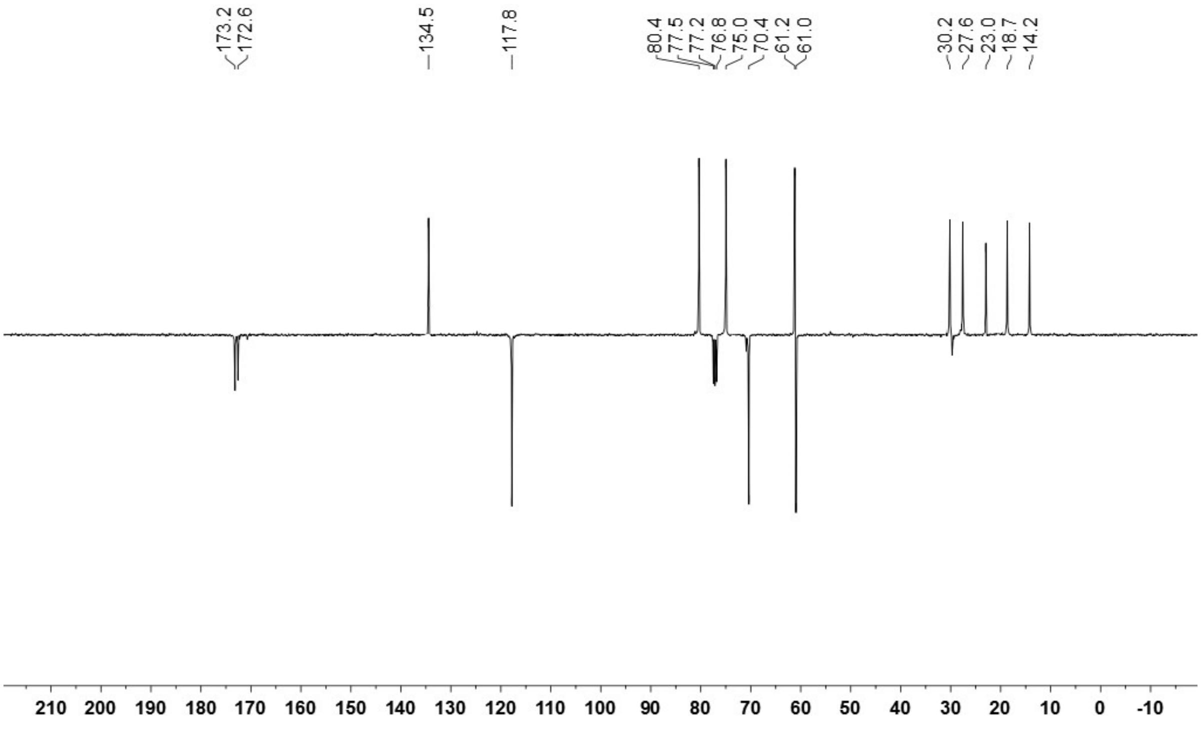

Compound 15g

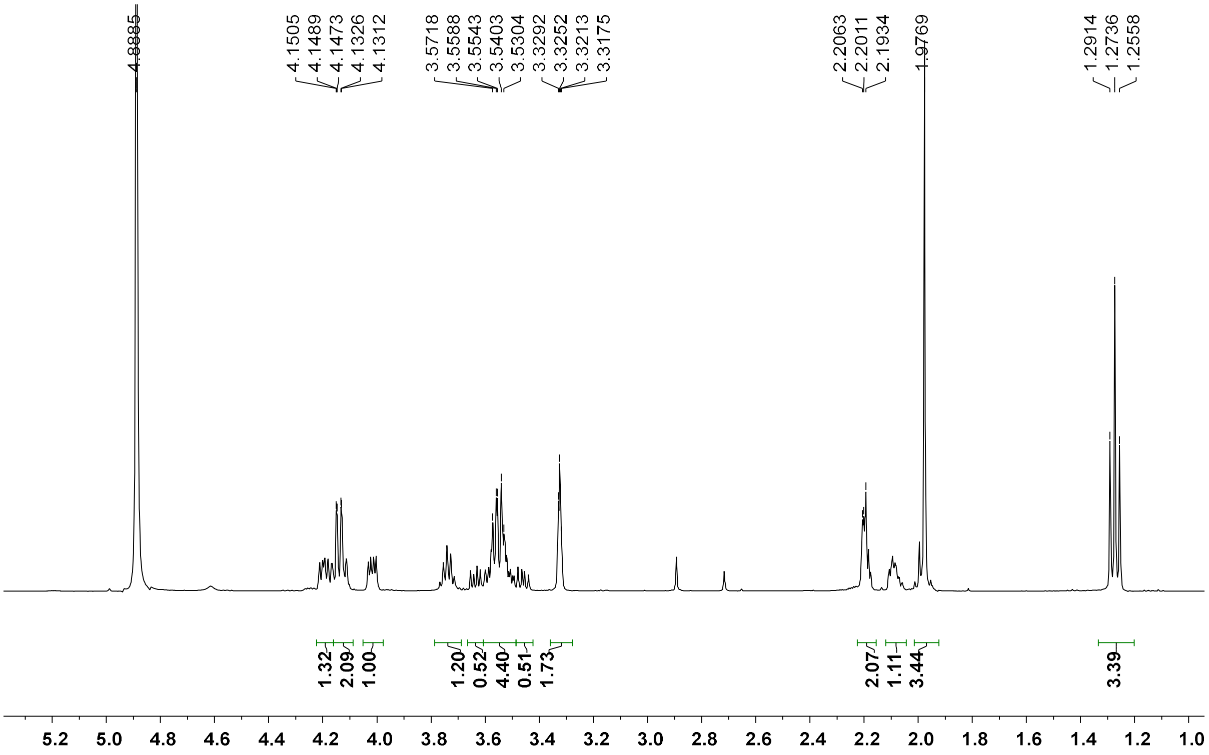

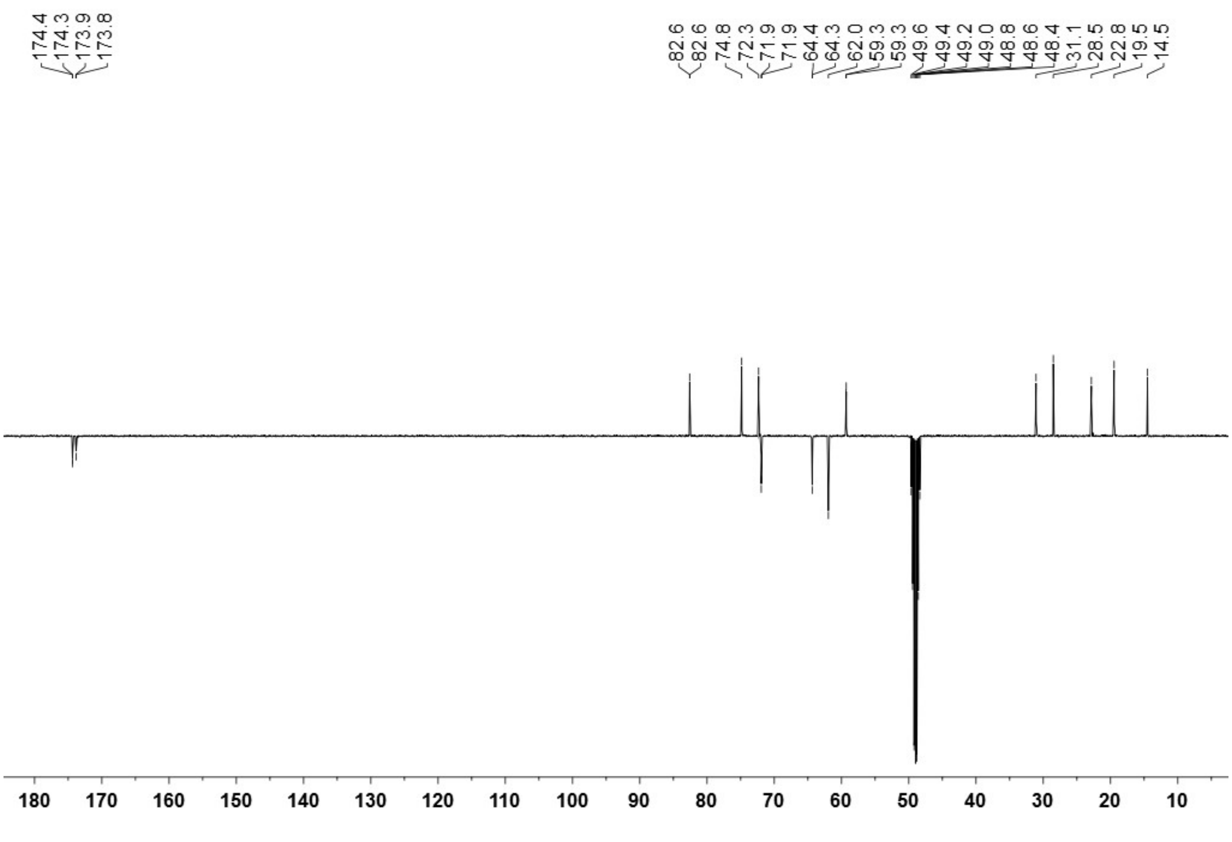

Compound 16a

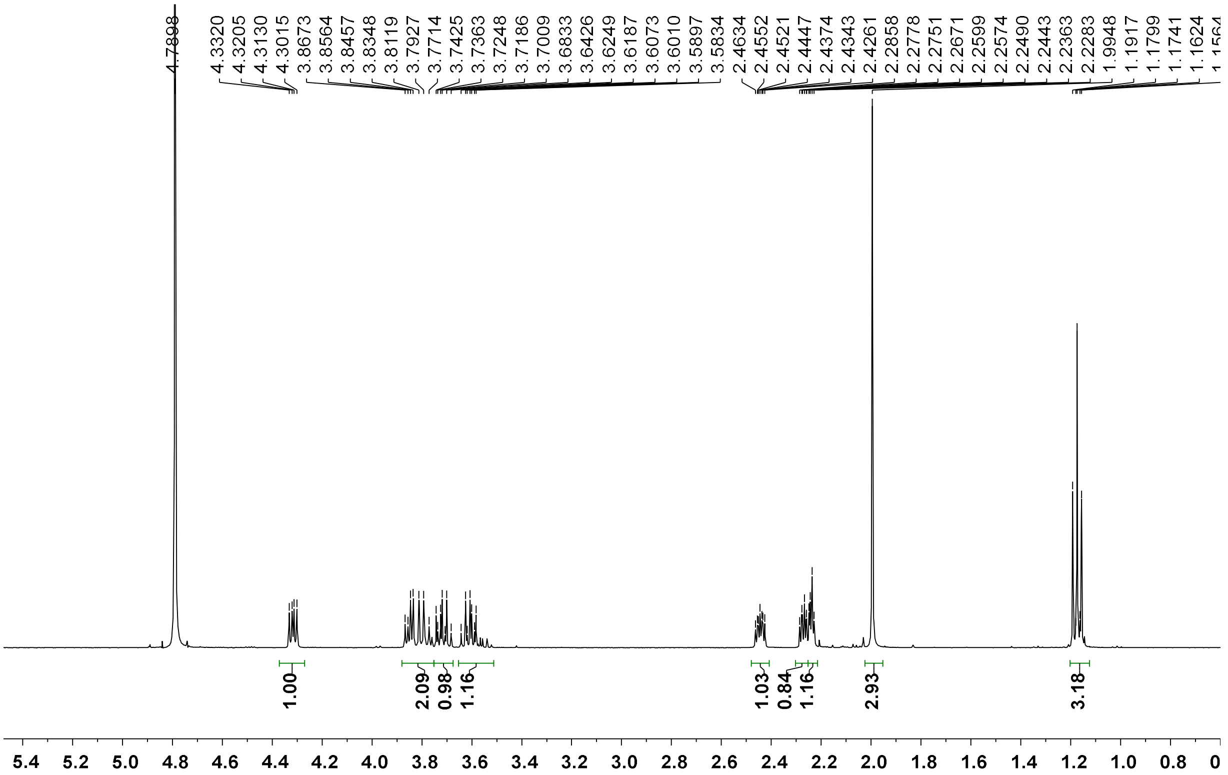

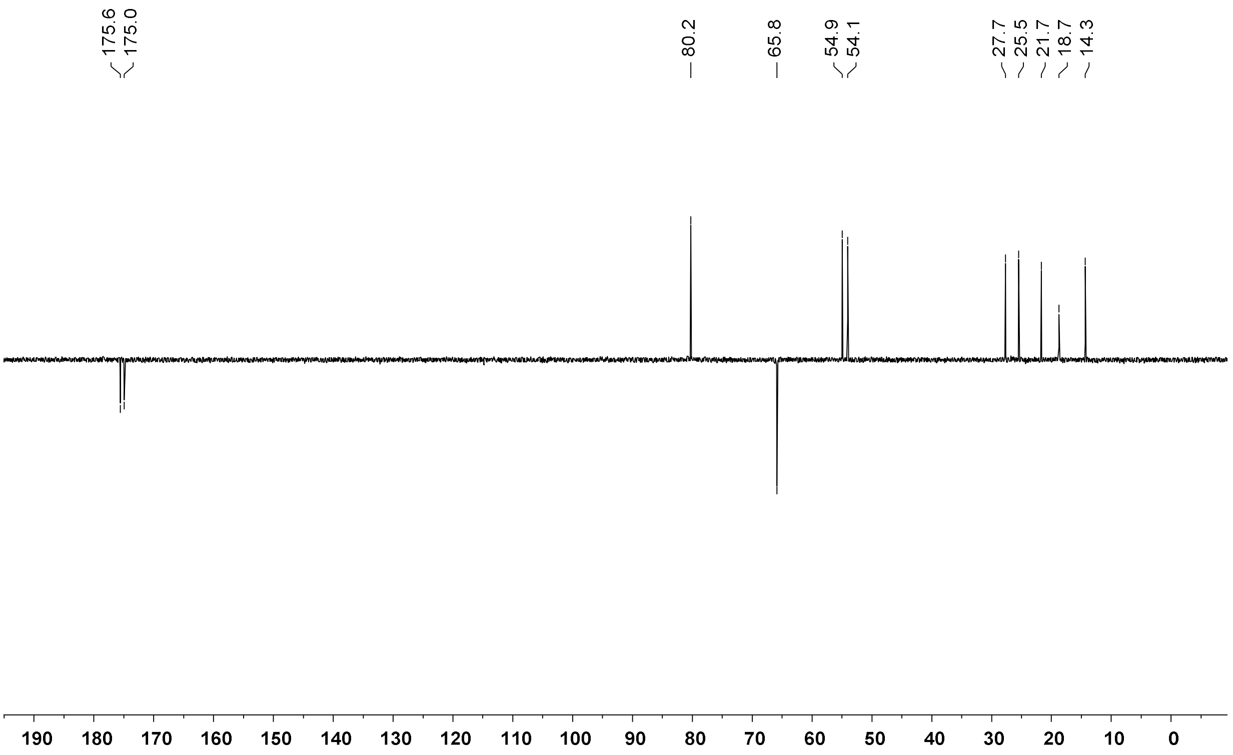

Compound 16b

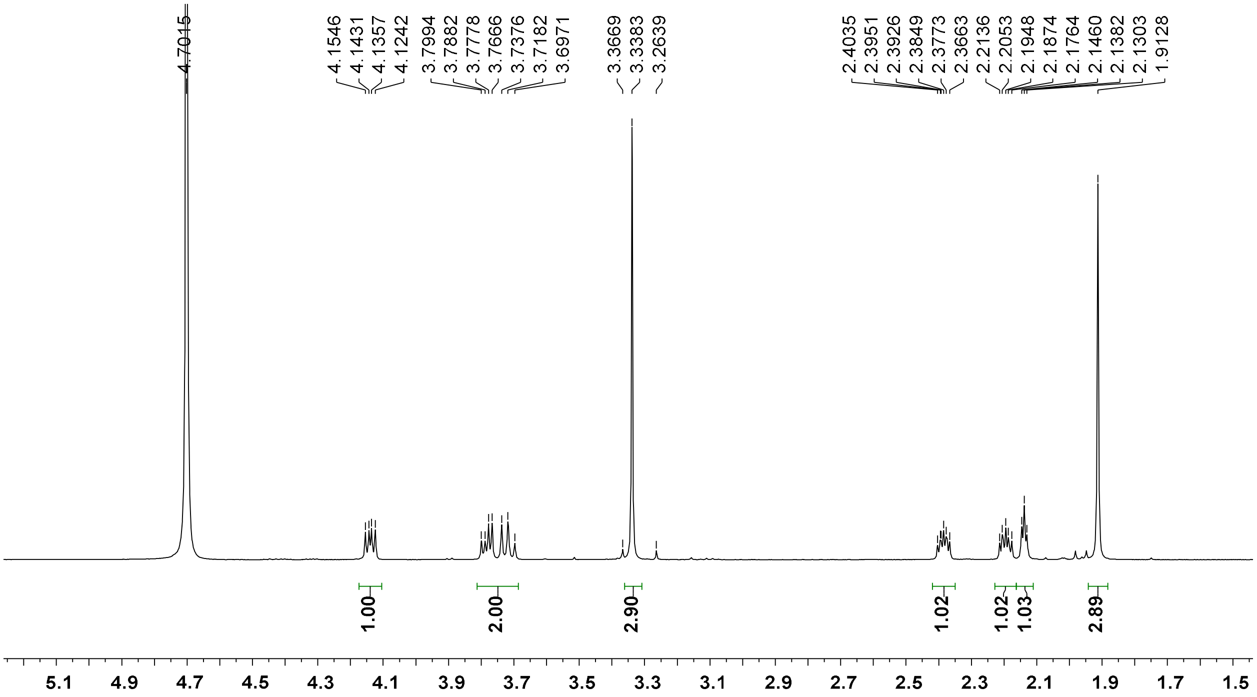

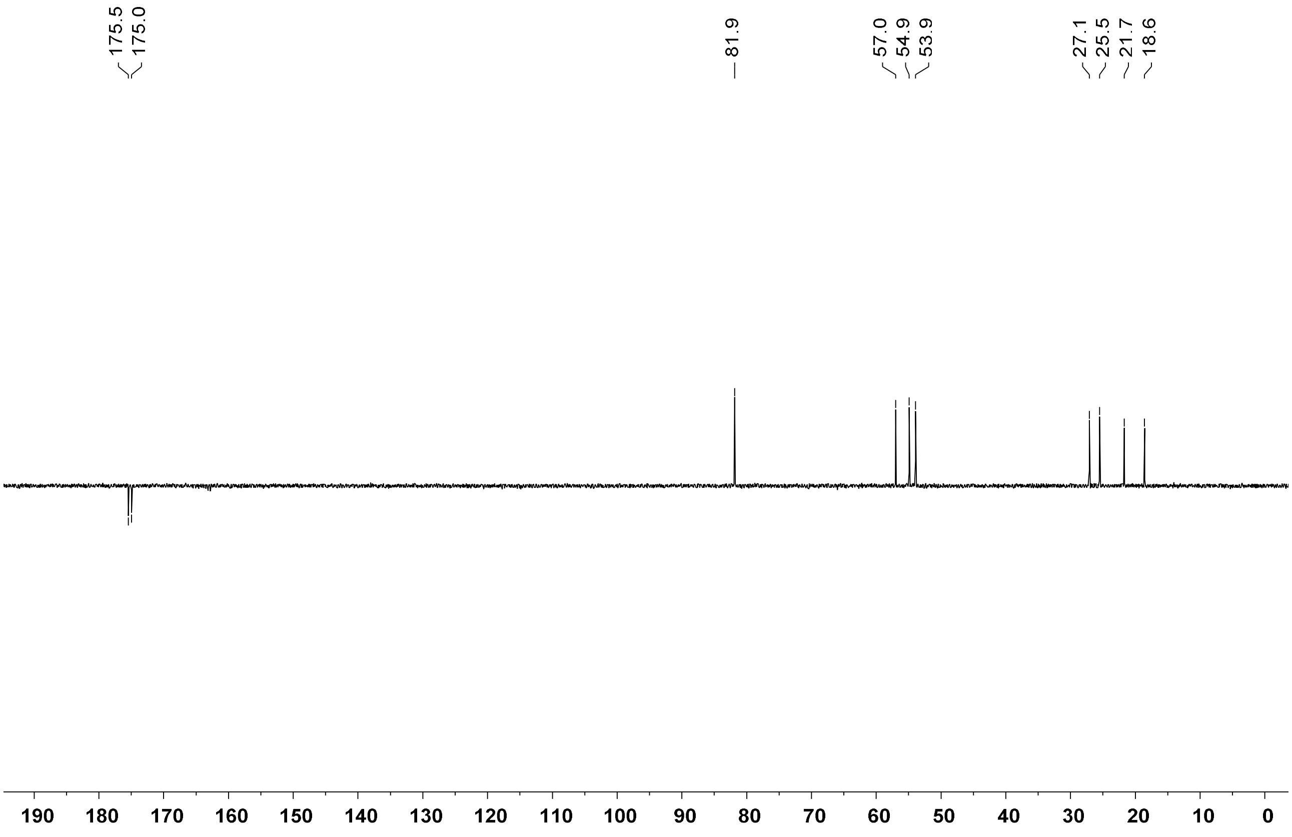

Compound 16c

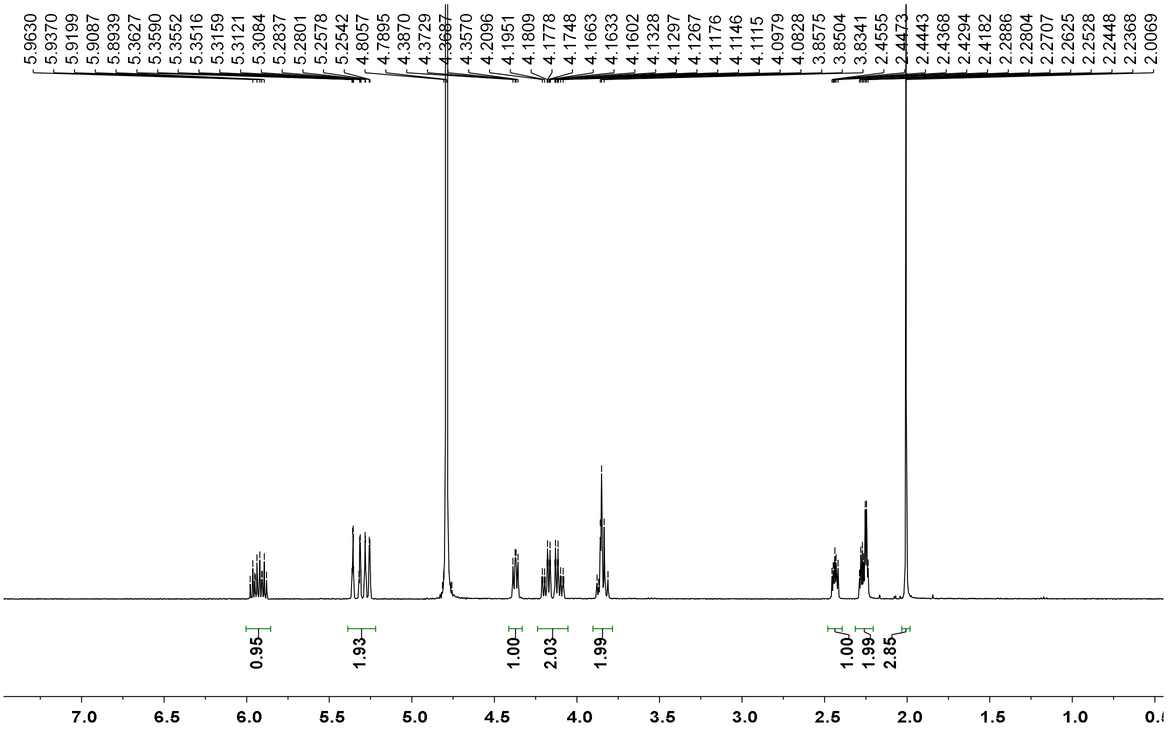

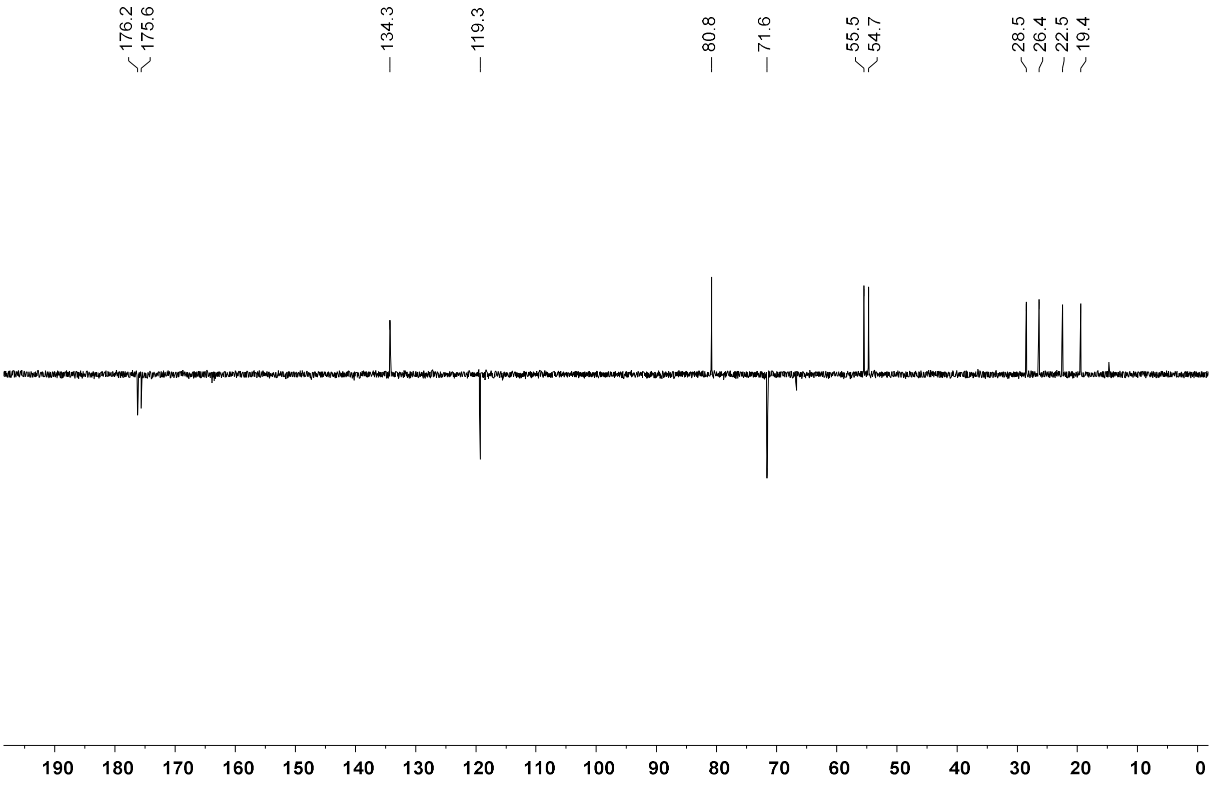

Compound 16d

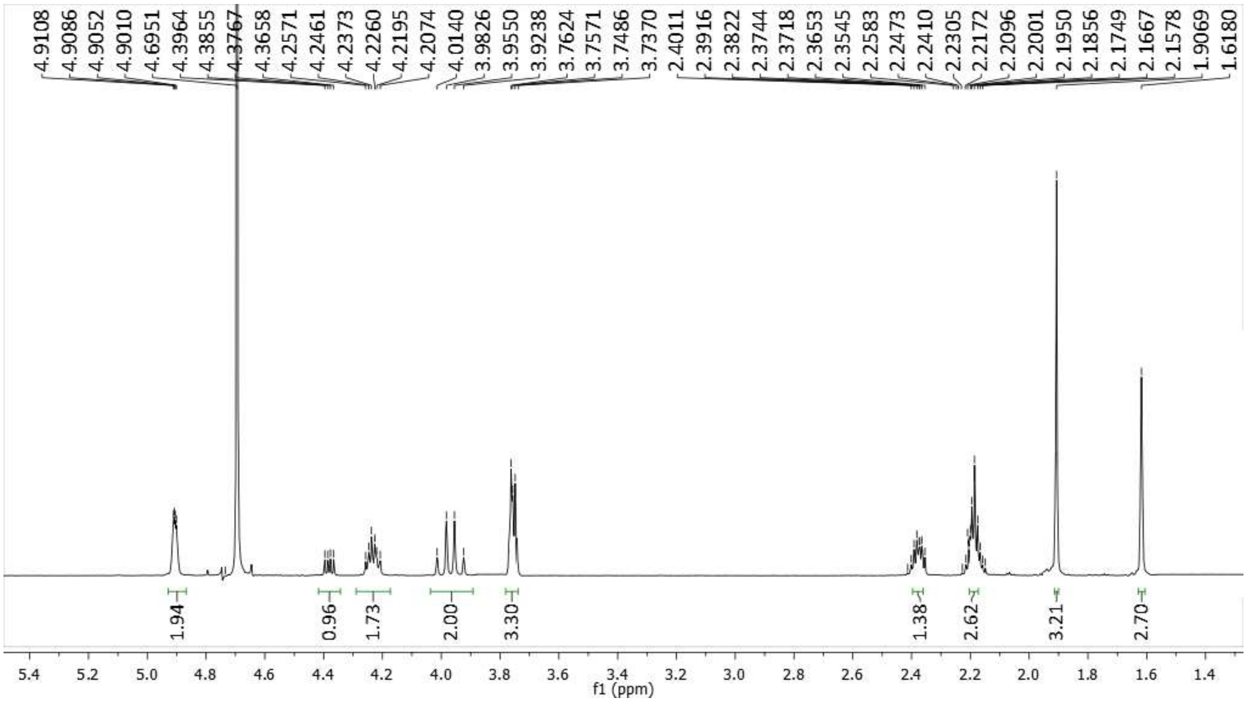

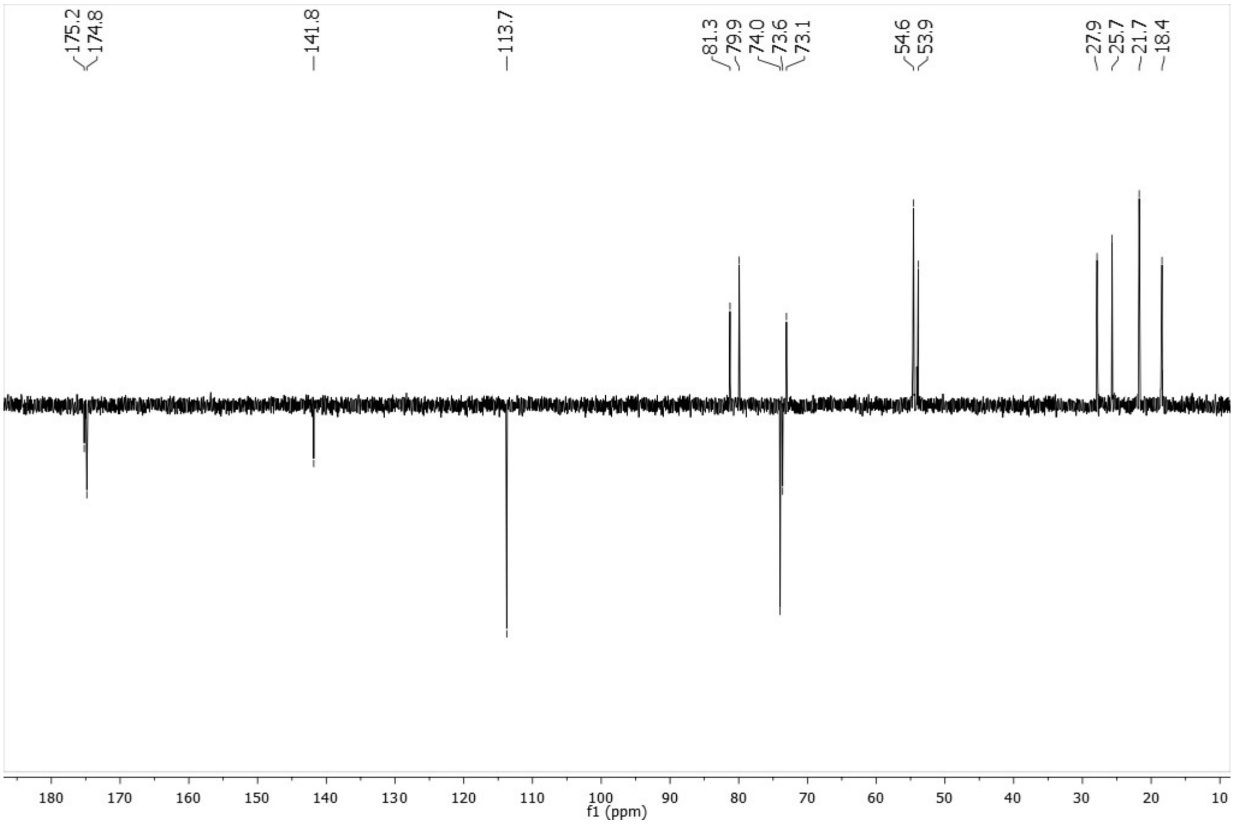

Compound 16e

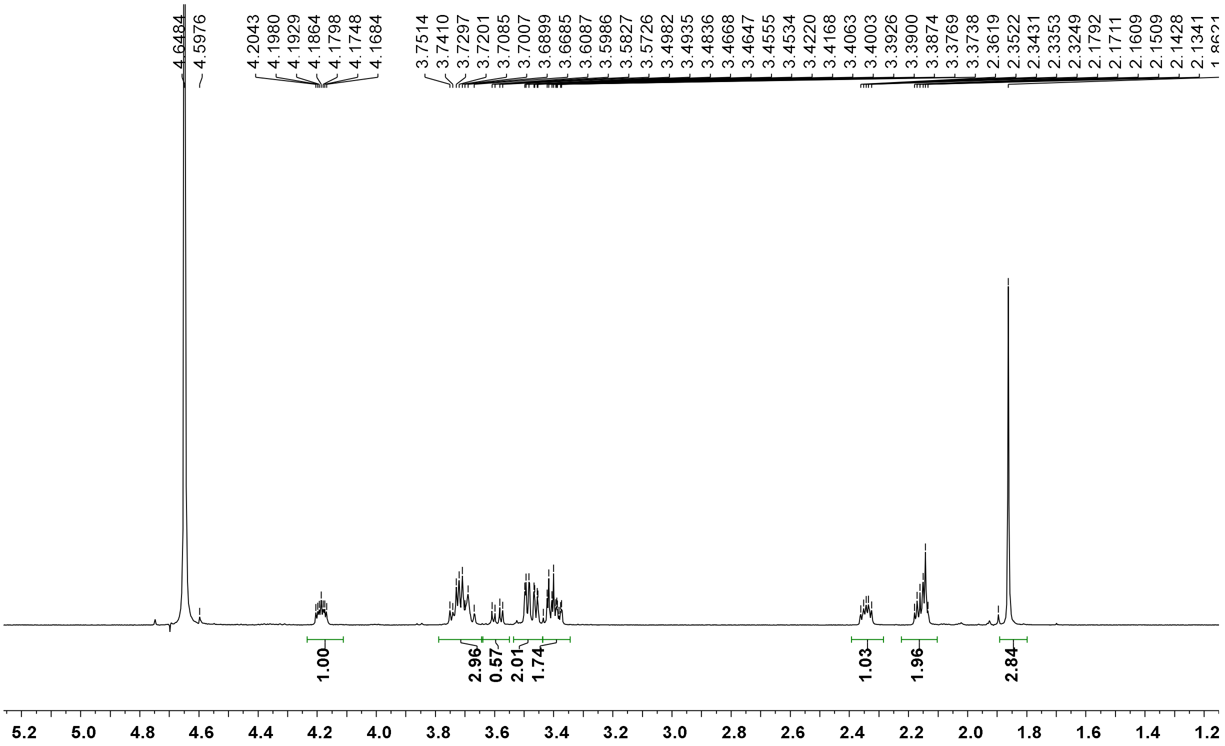

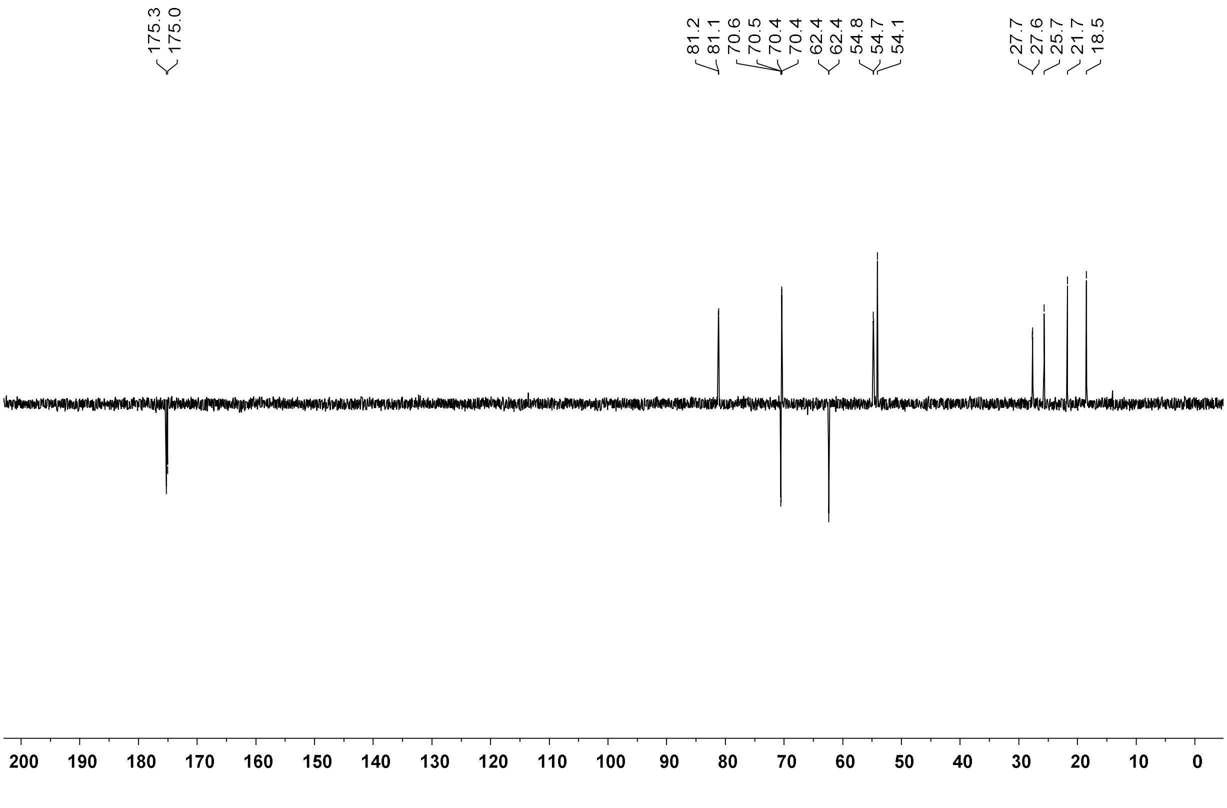

Compound 16f

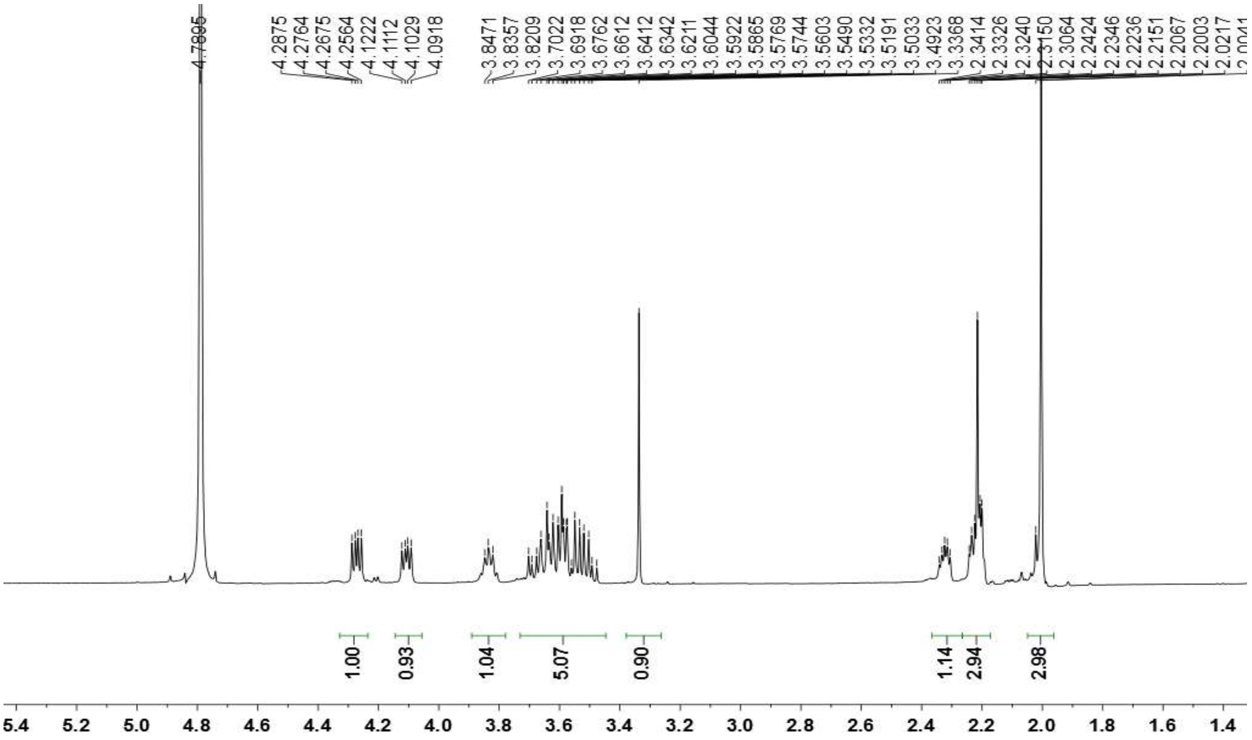

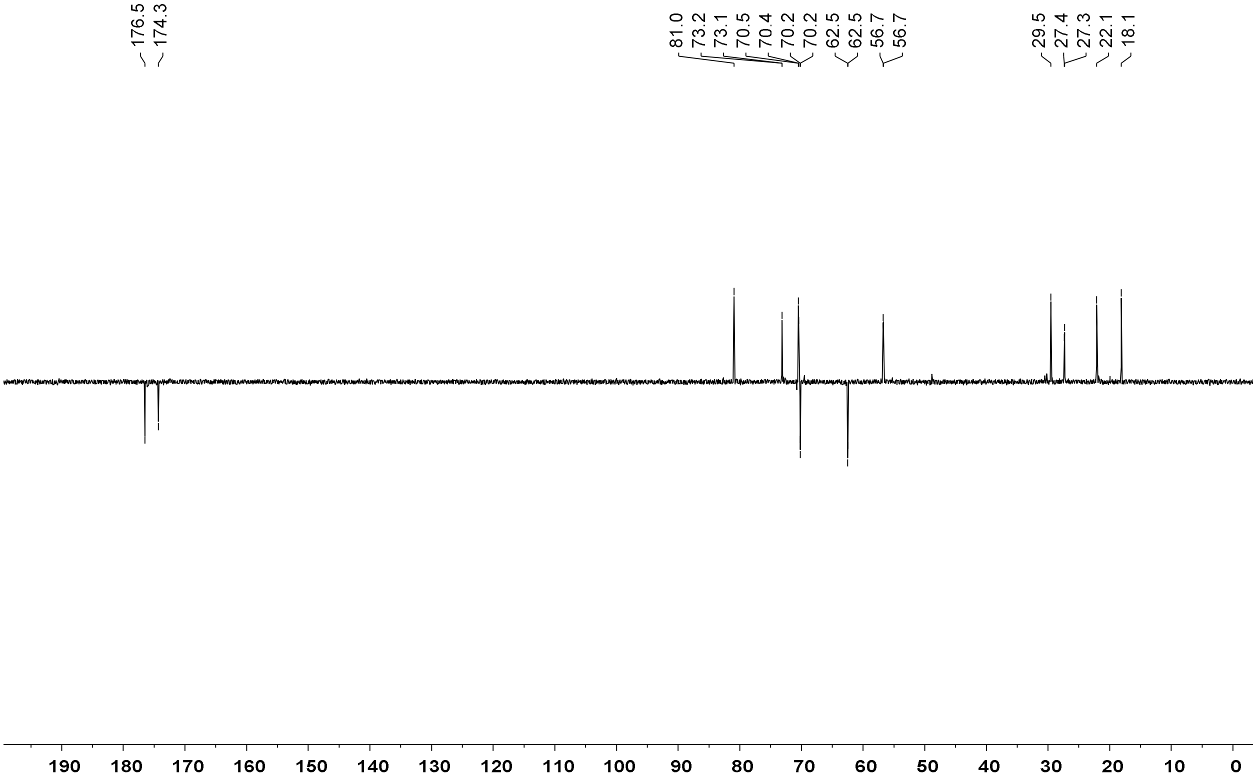

Compound 18a

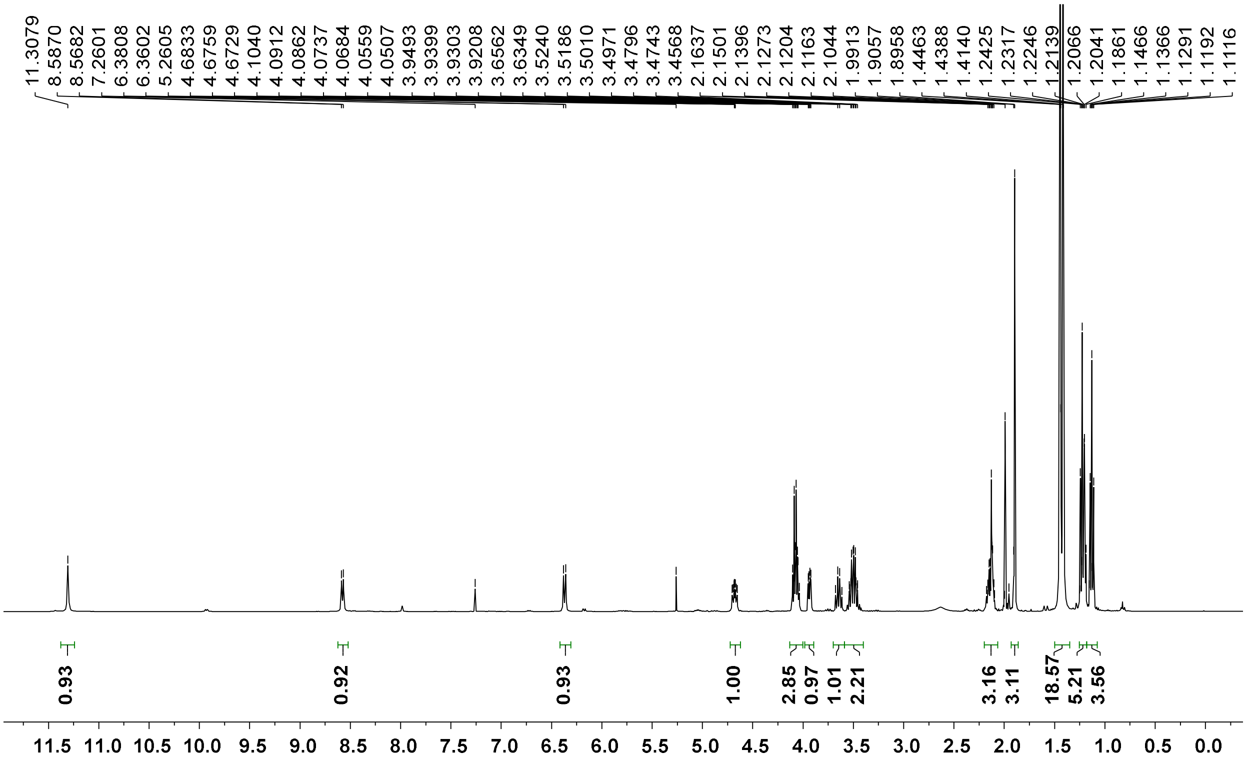

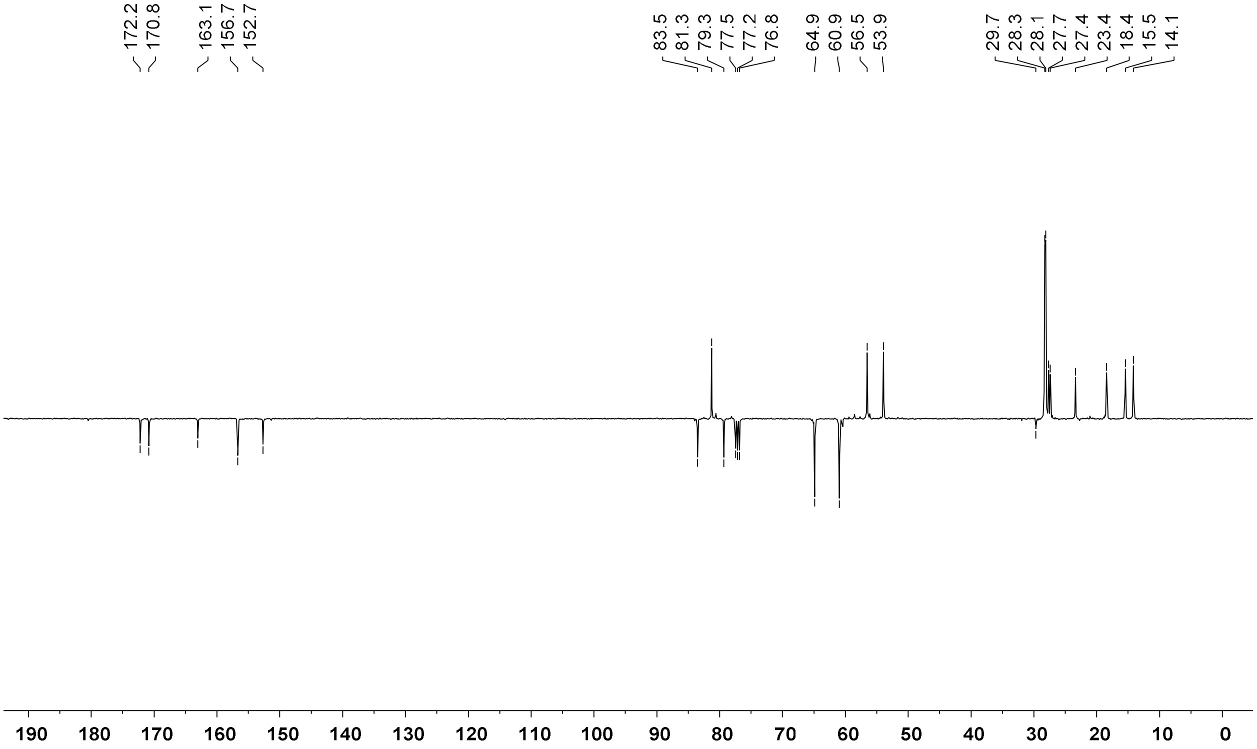

Compound 18b

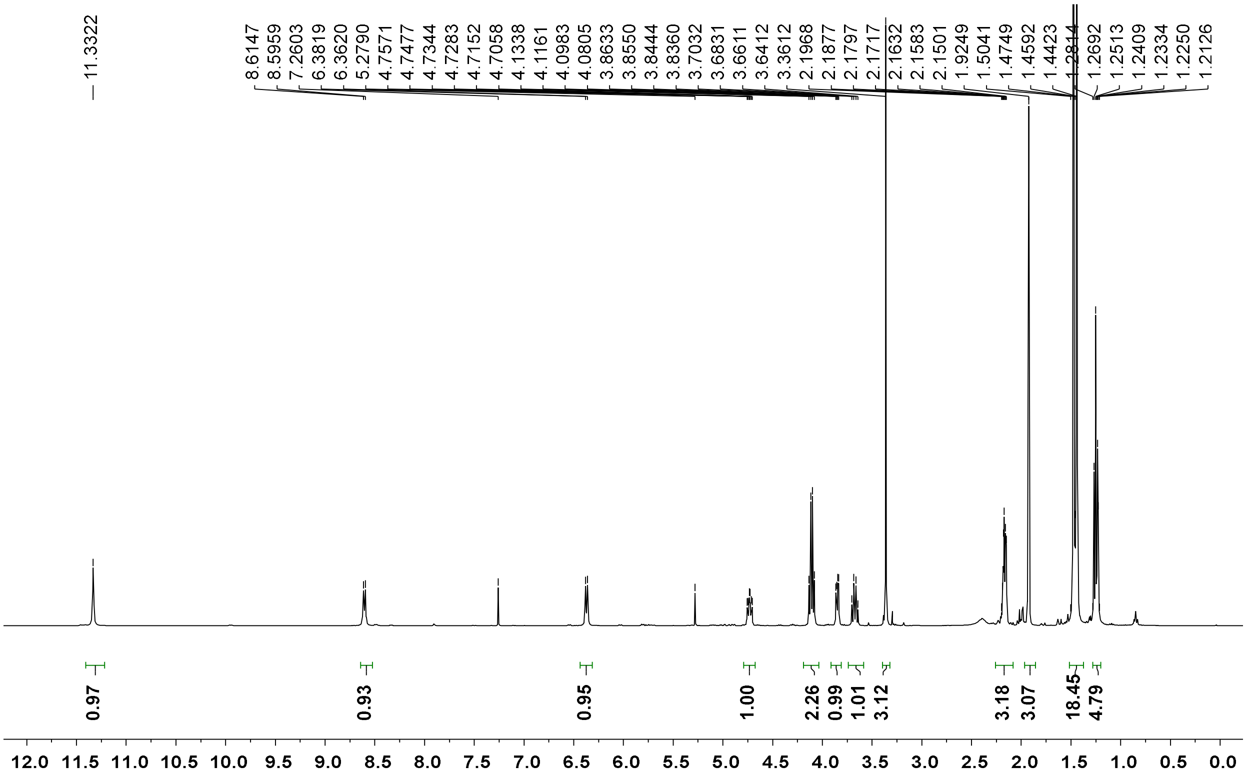

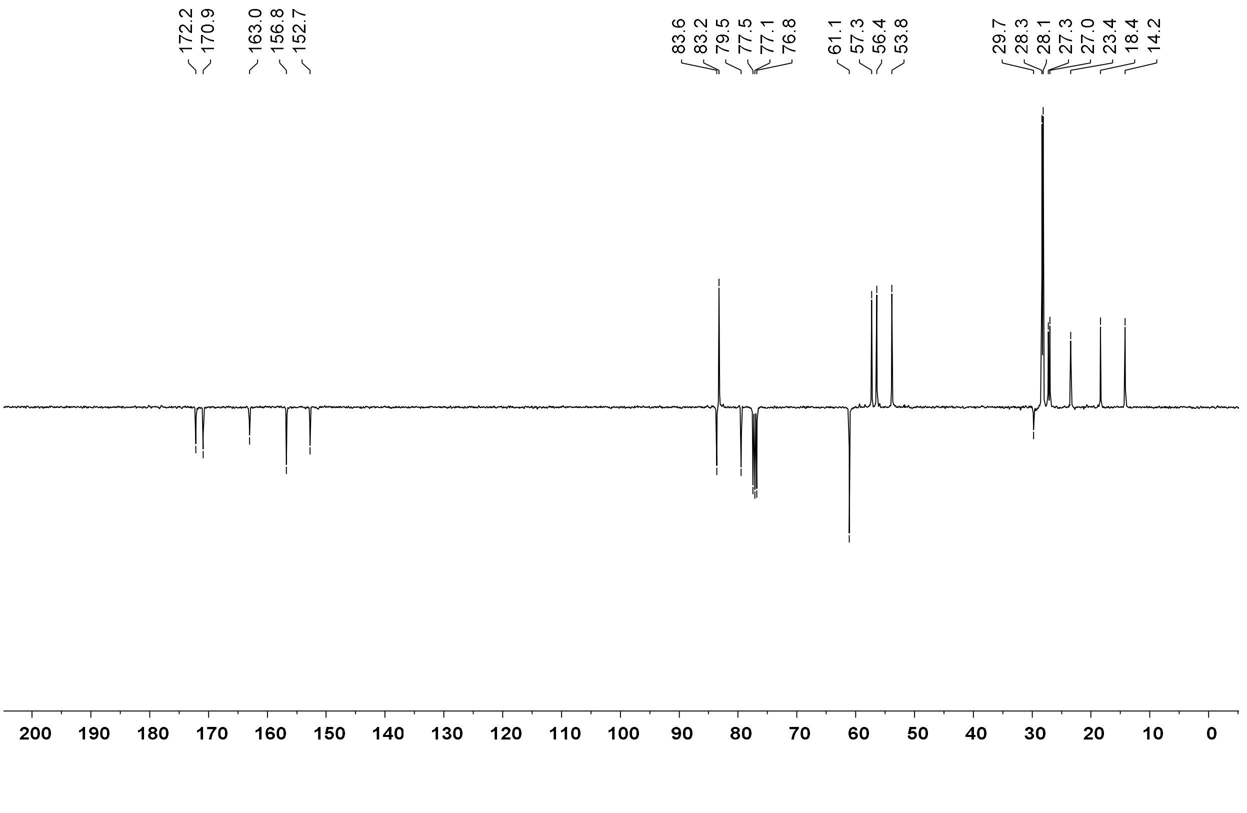

Compound 18c

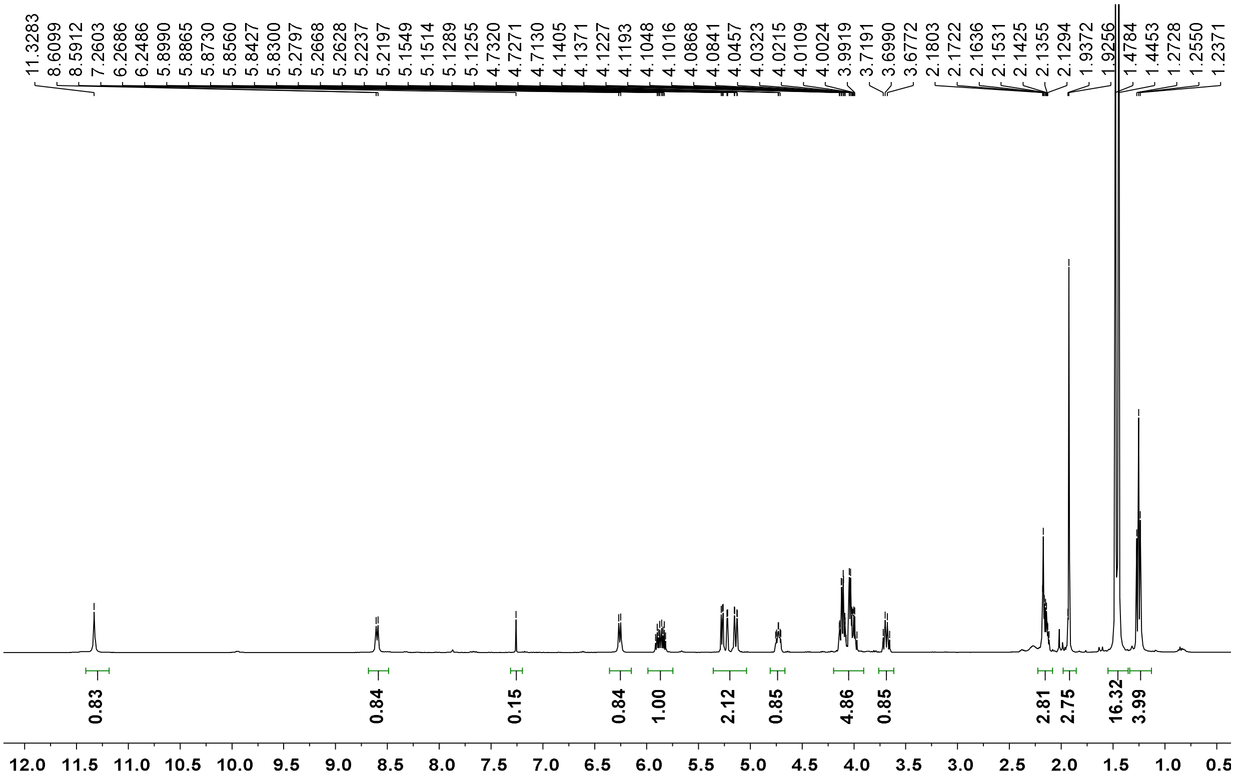

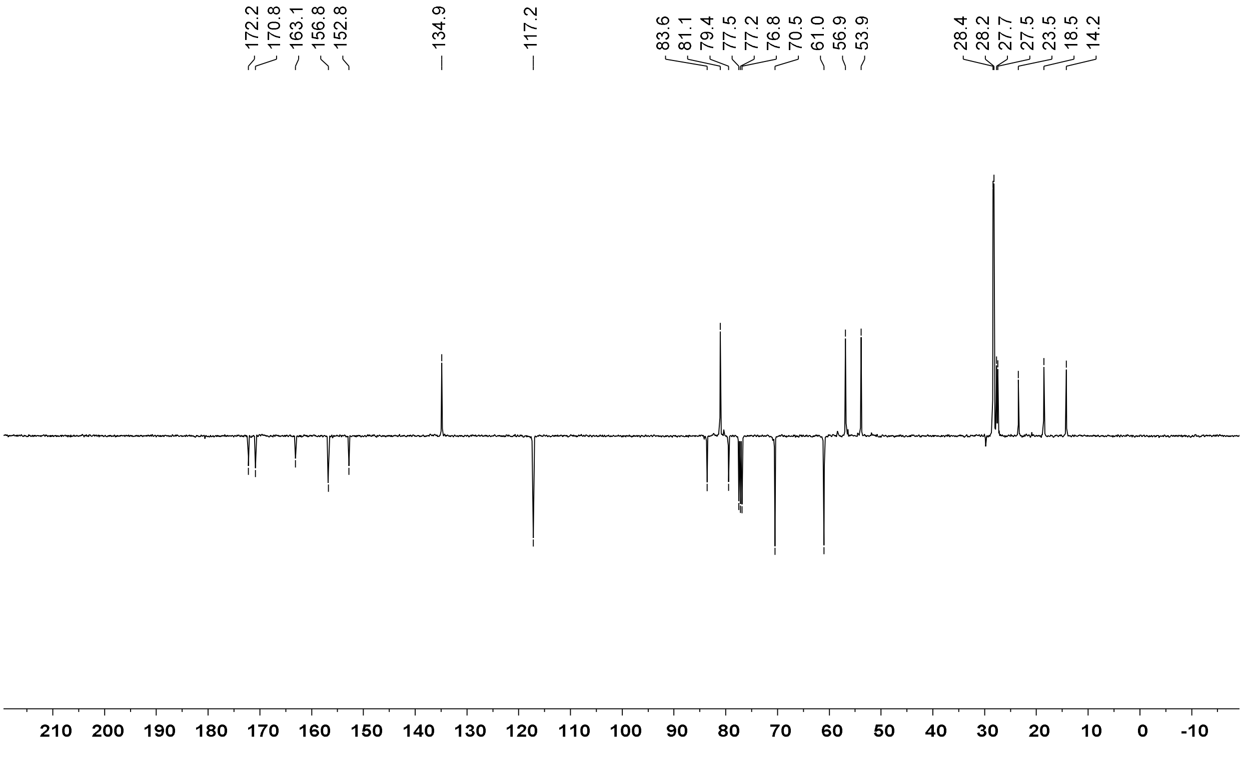

Compound 18d

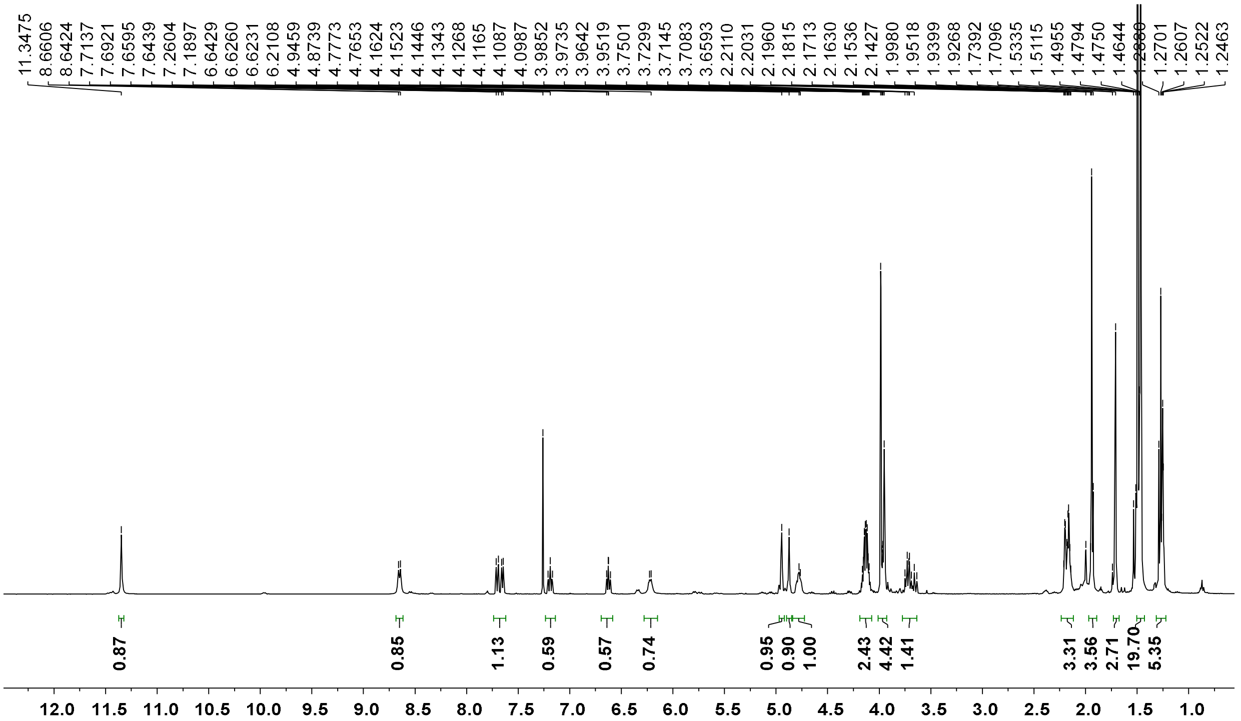

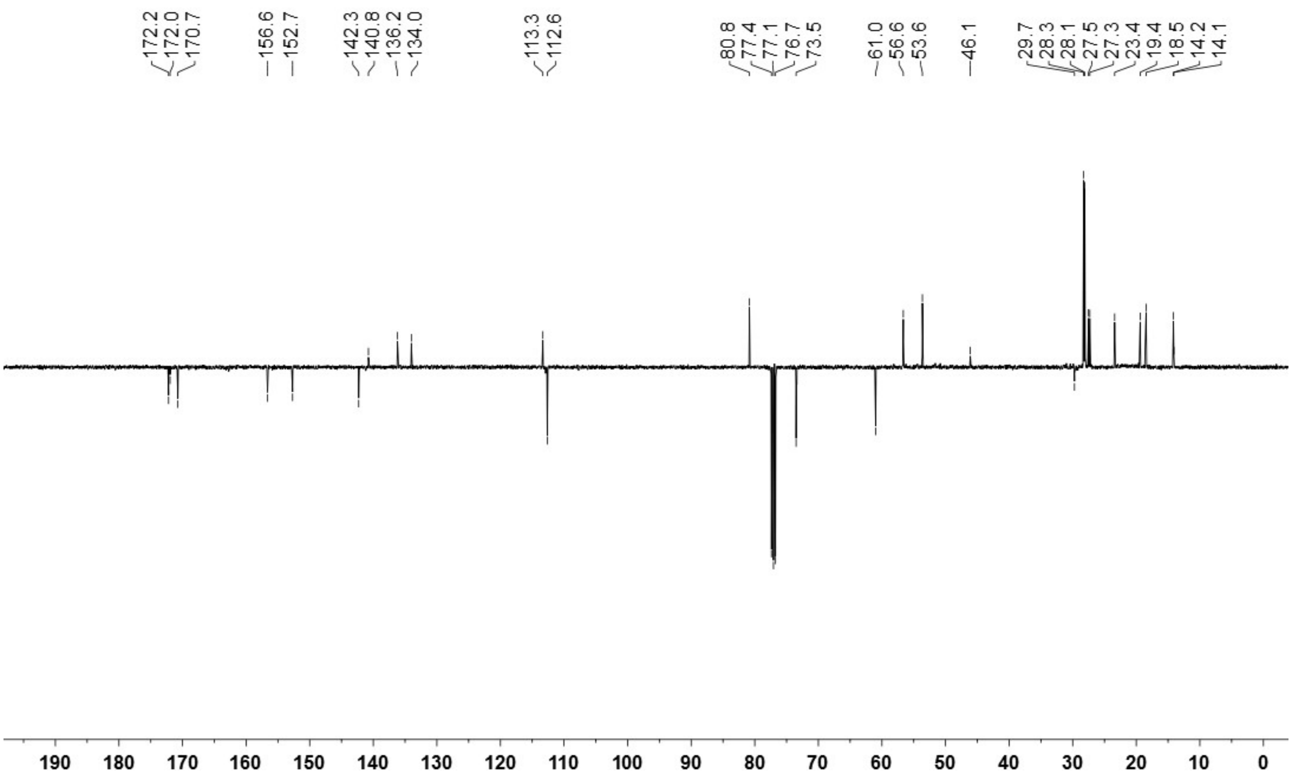

Compound 18e

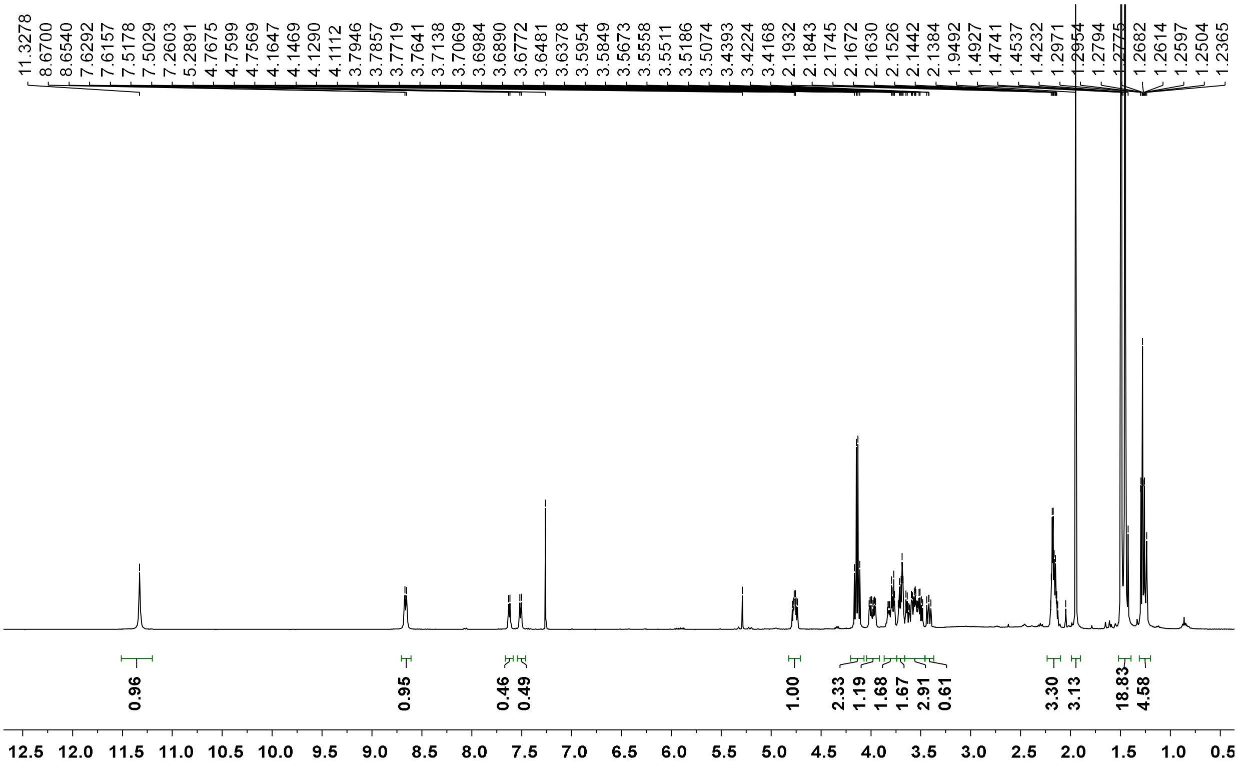

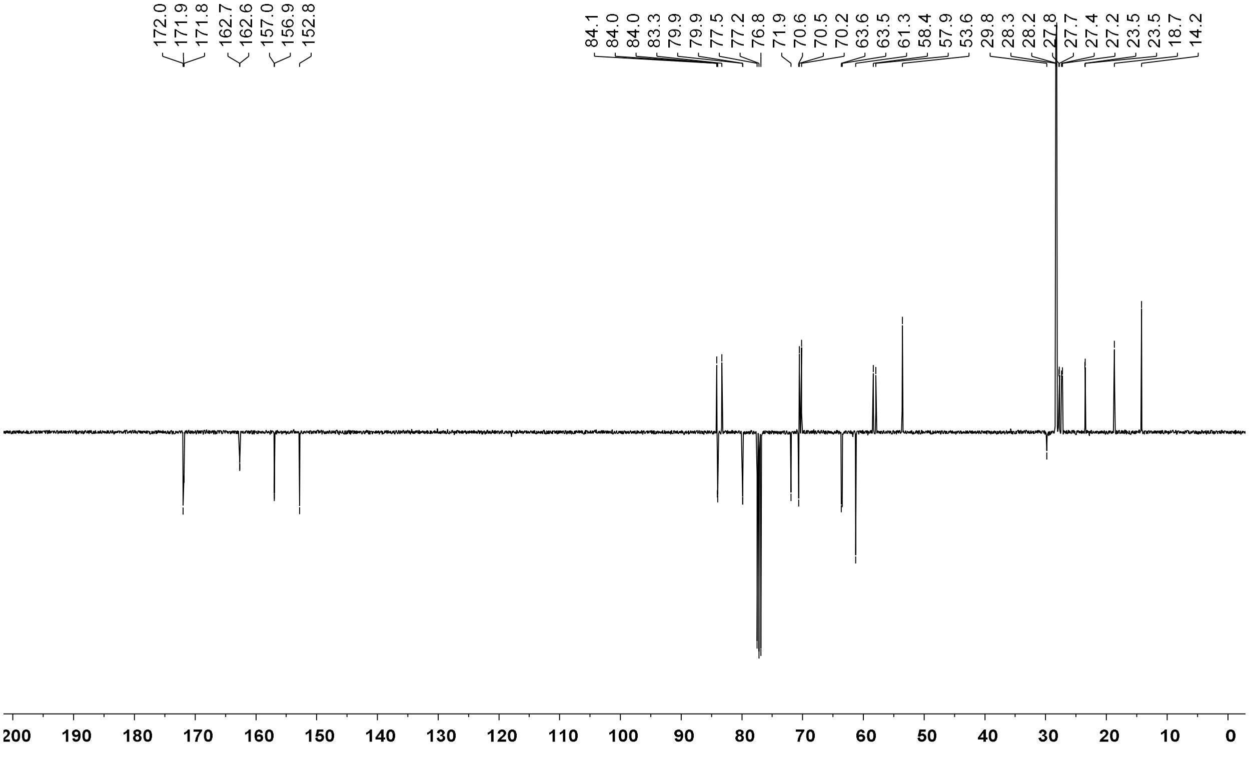

Compound 19a

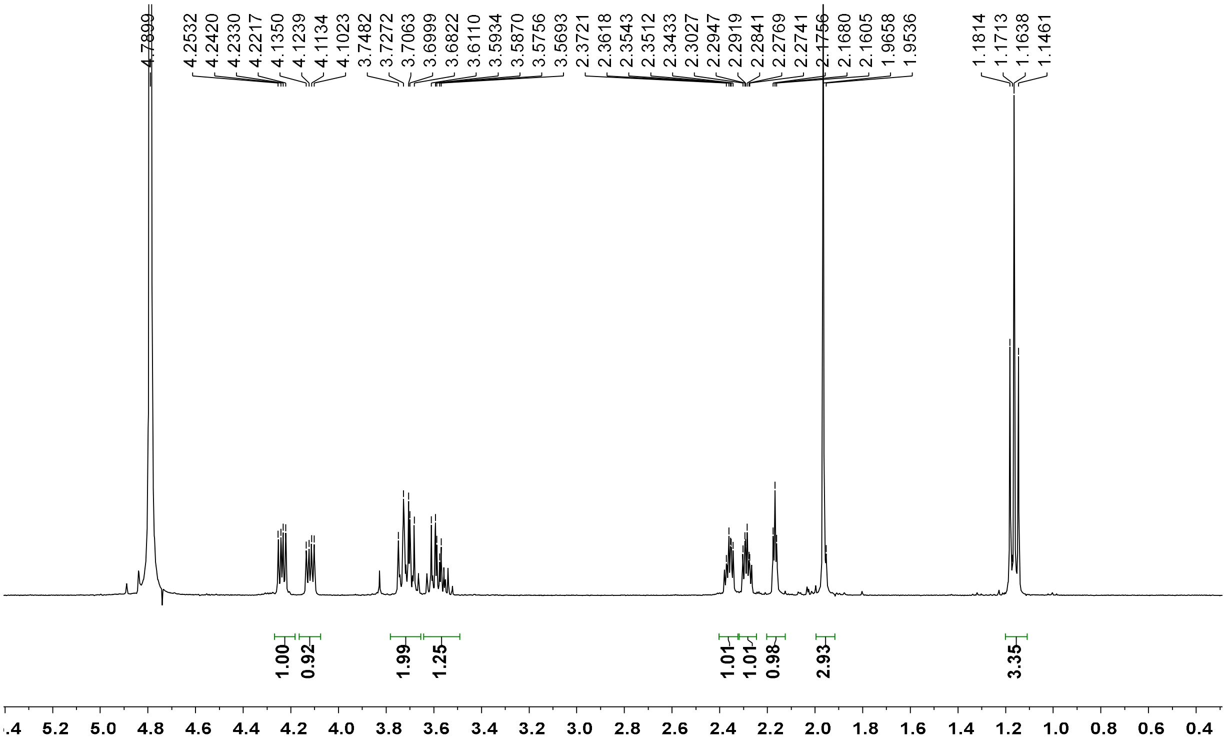

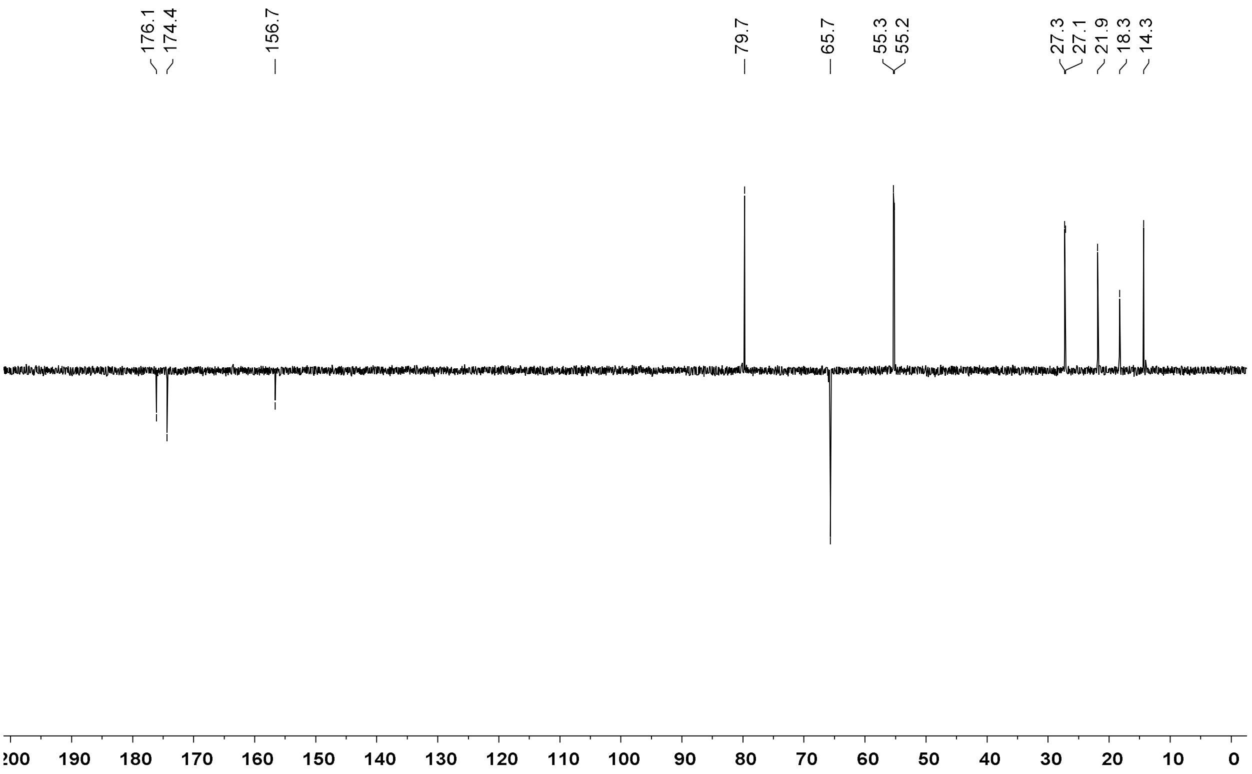

Compound 19b

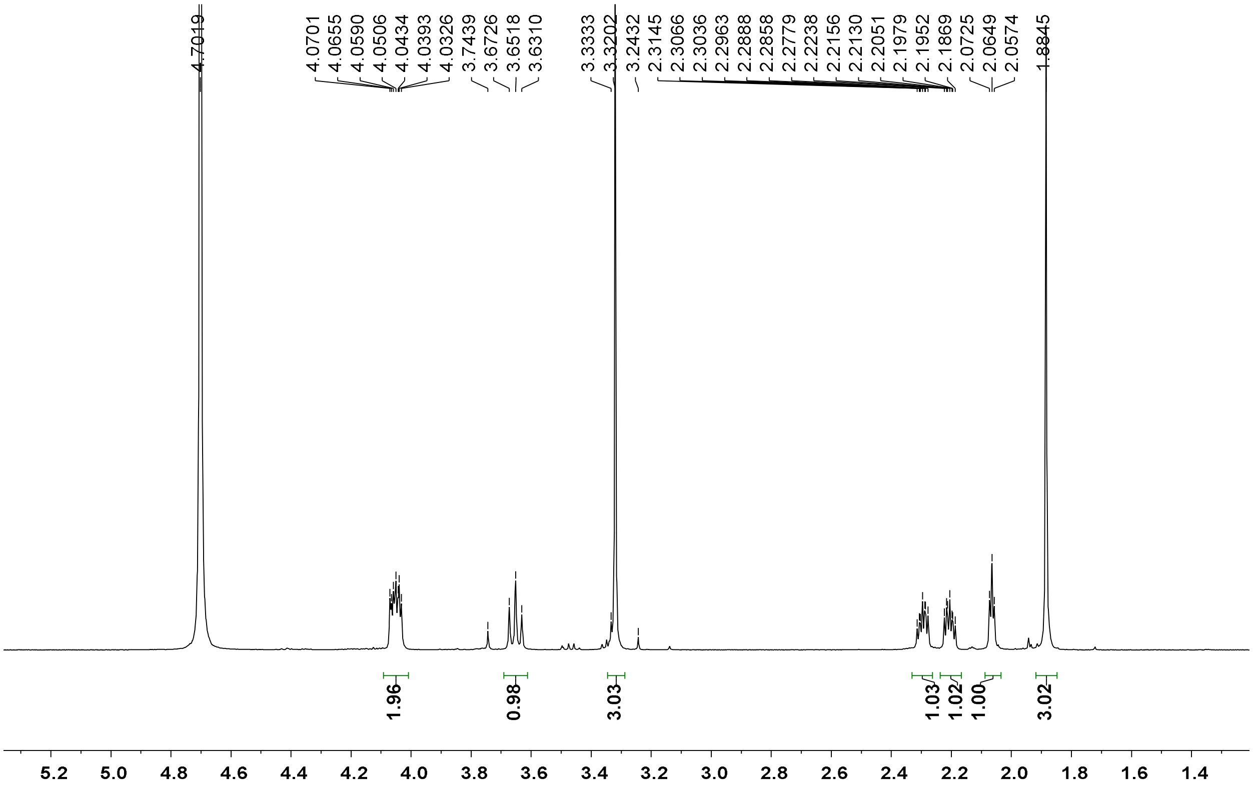

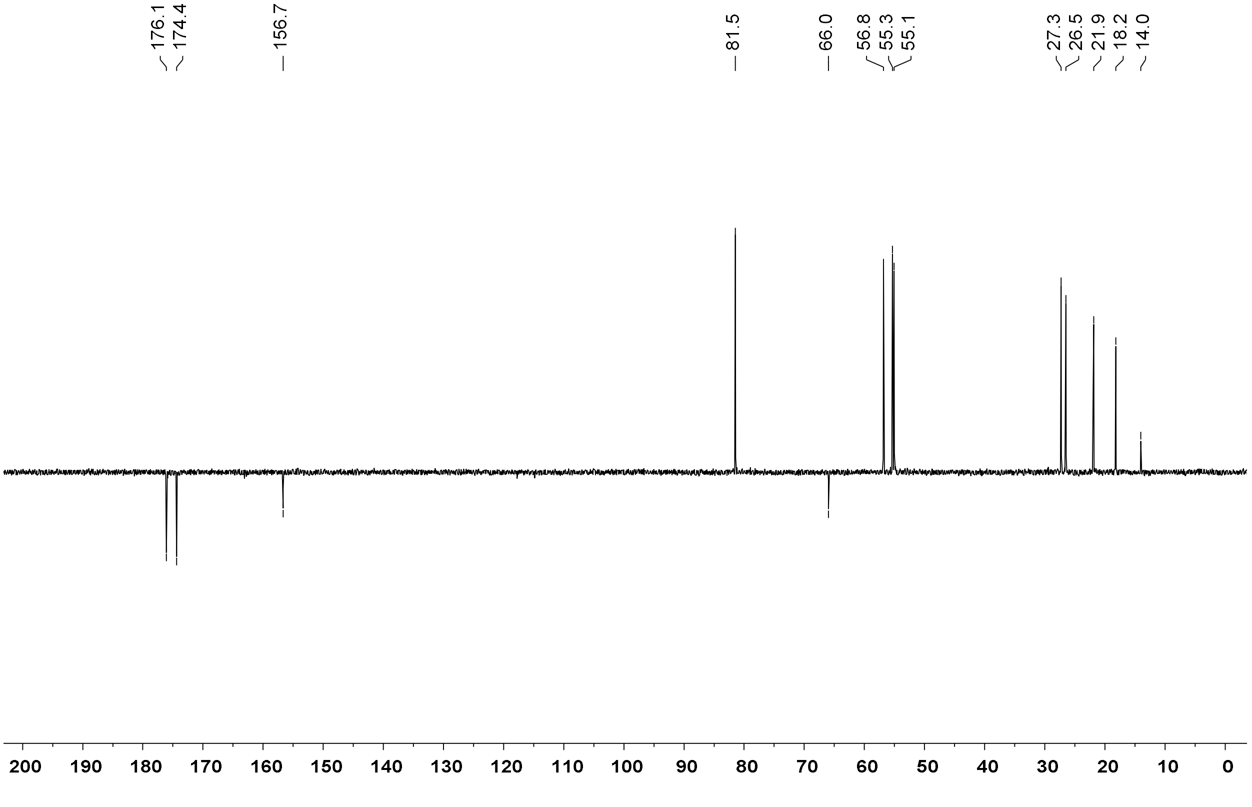

Compound 19c

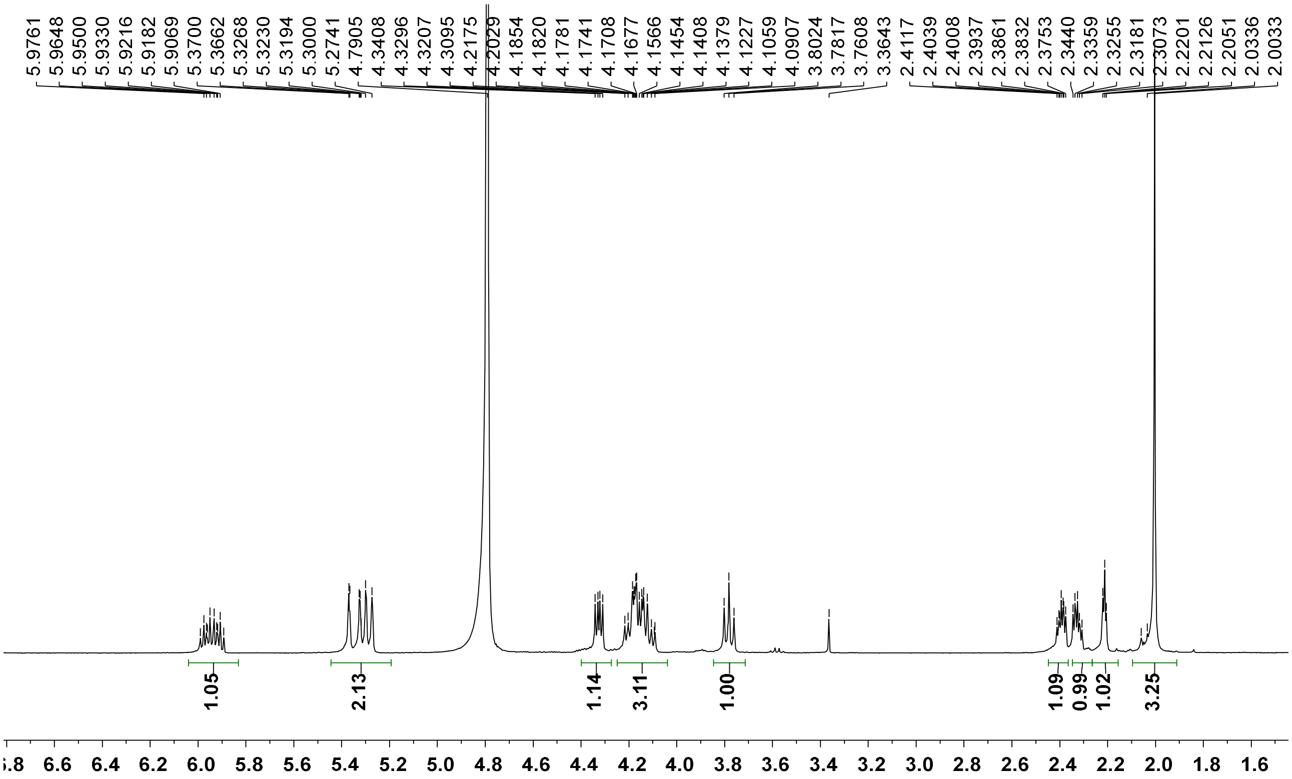

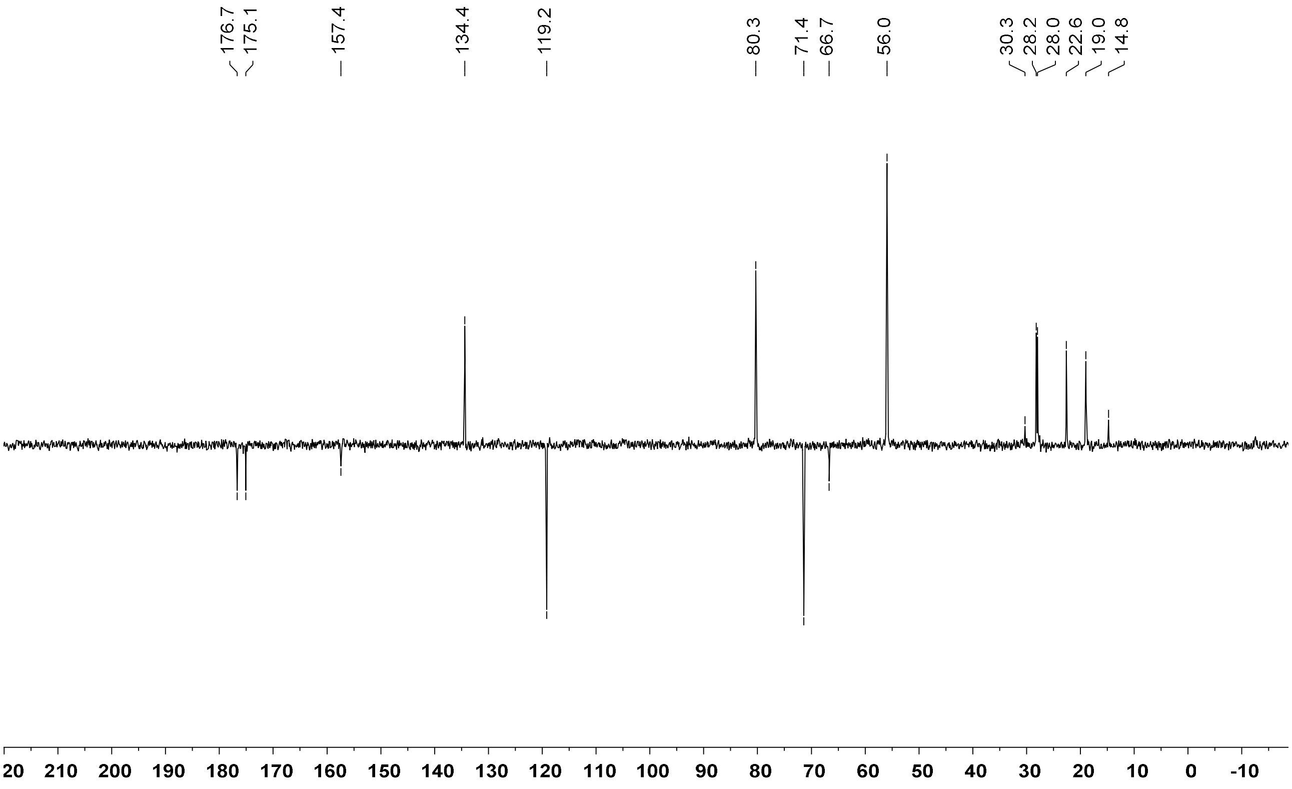

Compound 19d

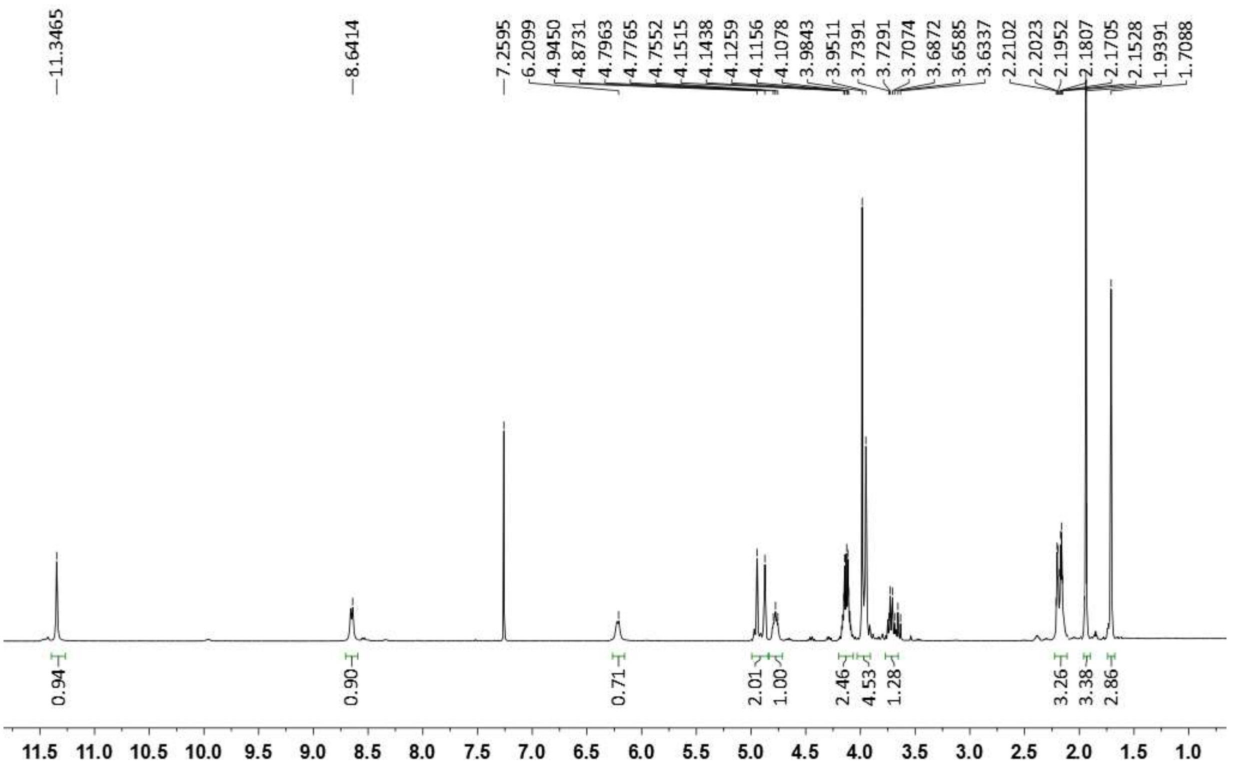

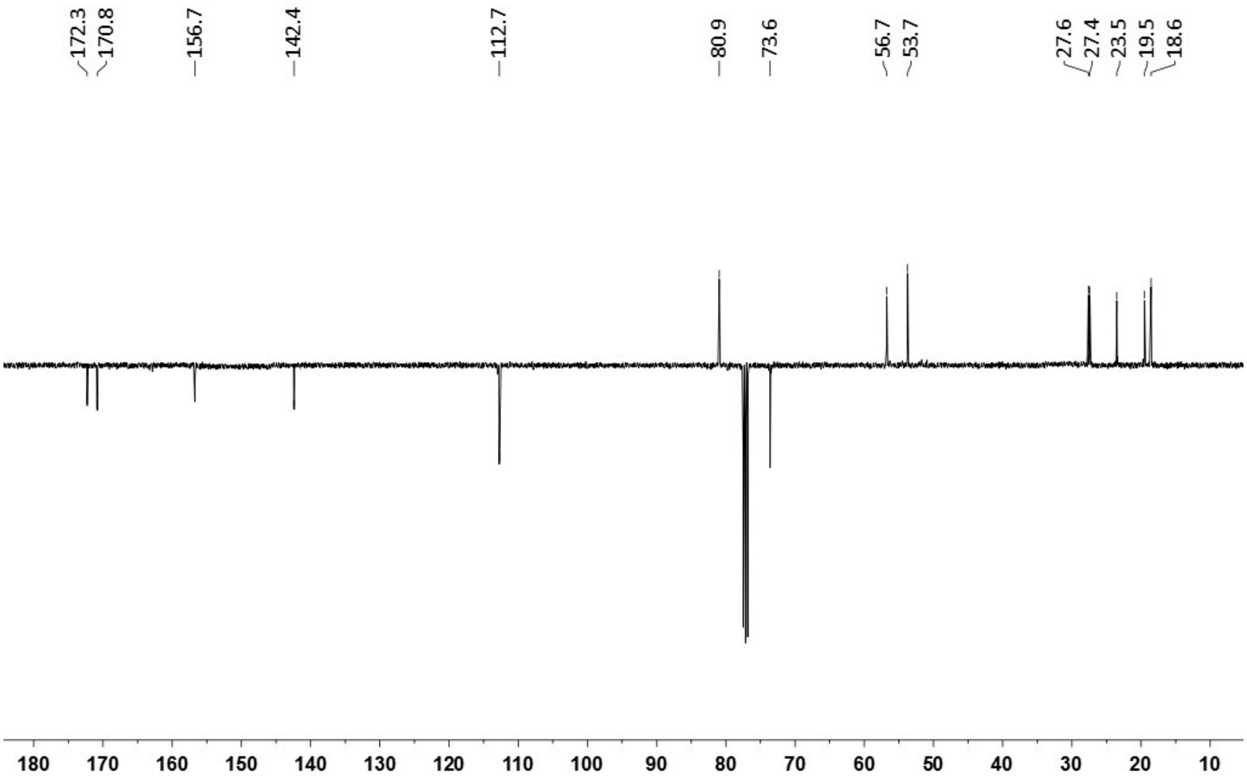

Compound 19e

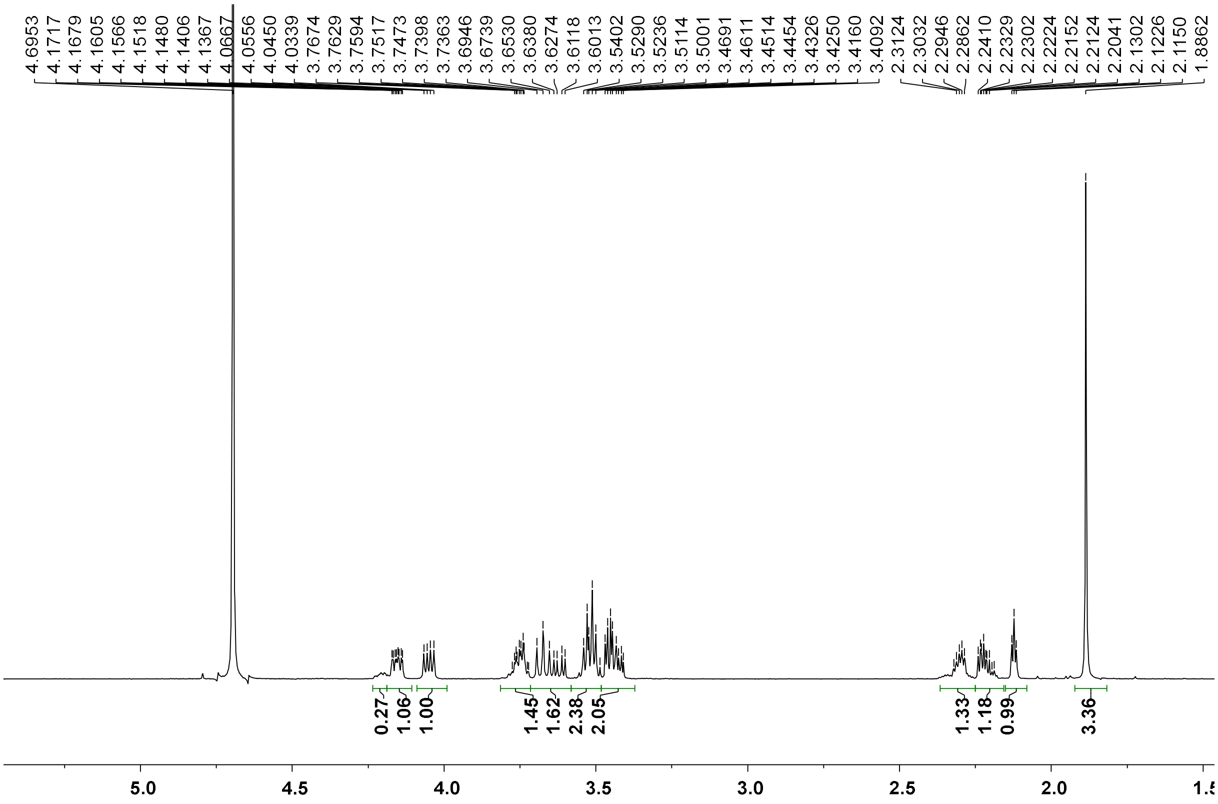

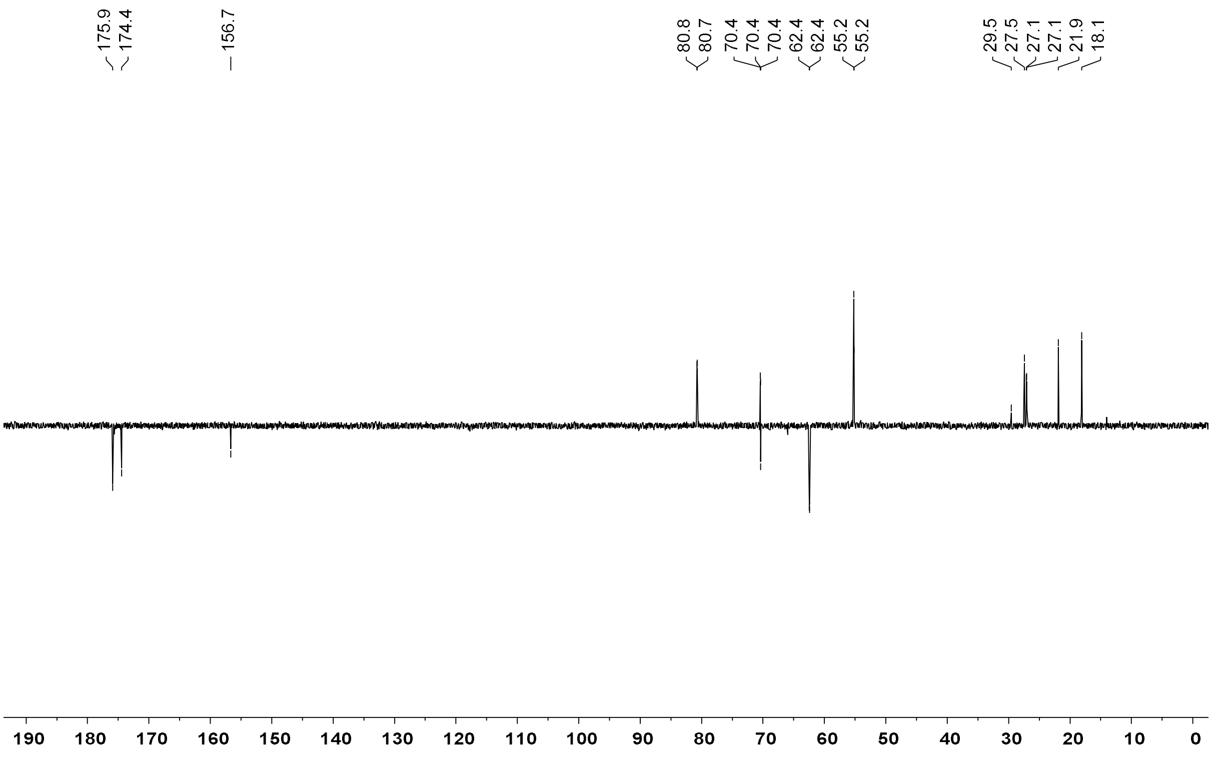

Supplement: S3 File — (PDF) [file pone.0193623.s003.pdf]
